# An aerosolized dual-action autotaxin inhibitor-PPARγ agonist for the treatment of pulmonary fibrosis

**DOI:** 10.1016/j.xcrm.2026.102778

**Published:** 2026-04-30

**Authors:** Alexios N. Matralis, Elli-Anna Stylianaki, Eleni M. Ladopoulou, Paraskevi Kanellopoulou, Stefanos Smyrniotis, Christiana Magkrioti, Konstantinos D. Papavasileiou, Sabine Willems, Juan Pablo Rincon Pabon, Dimitris Nastos, Alexandros Galaras, Céline Moro, Skarlatos G. Dedos, Eleanna Kaffe, Pantelis Hatzis, Hanan Osman-Ponchet, Daniel Merk, Argyris Politis, Antreas Afantitis, Ioulia Tseti, Katerina M. Antoniou, Athol U. Wells, Vassilis Aidinis

**Affiliations:** 1 Institute for Bioinnovation, Biomedical Sciences Research Center “Alexander Fleming”, 16672 Athens, Greece; 2 Department of Pharmacy, University of Patras, 26500 Patras, Greece; 3 Institute for Fundamental Biomedical Research, Biomedical Sciences Research Center “Alexander Fleming”, 16672 Athens, Greece; 4 Department of Biology, National & Kapodistrian University of Athens, 15772 Athens, Greece; 5 Department of ChemoInformatics, Novamechanics Ltd., 1070 Nicosia, Cyprus; 6 Department of Pharmacy, Ludwig-Maximilians-Universität München, 81377 Munich, Germany; 7 Faculty of Biology, Medicine and Health, University of Manchester, Manchester M13 9PT, UK; 8 Manchester Institute of Biotechnology, University of Manchester, Manchester M1 7DN, UK; 9 Department of Biochemistry and Biotechnology, University of Thessaly, 41334 Larisa, Greece; 10 PKDERM, 80 Route des Lucioles, 06560 Valbonne, France; 11 Perelman School of Medicine, University of Pennsylvania, Philadelphia, PA 19104, USA; 12 Department of Pharmacy, Frederick University, 1036 Nicosia, Cyprus; 13 Uni-Pharma S.A., 14564 Athens, Greece; 14 Department of Respiratory Medicine, School of Medicine, University of Crete, 70013 Heraklion, Greece; 15 Interstitial Lung Disease Unit, Royal Brompton Hospital, London SW3 6NP, UK; 16 National Heart and Lung Institute, Imperial College London, London SW3 6LY, UK; 17 Margaret Turner Warwick Centre for Fibrosing Lung Disease, Imperial College London, London, UK; 18 DrugTrek PC, Patras Science Park, 26504 Rio, Greece

**Keywords:** idiopathic pulmonary fibrosis, IPF, metabolic disorders, Autotaxin, ATX, peroxisome-proliferator-activated receptor γ, PPARγ, bleomycin, BLM, inhalation, chemoinformatics, drug repositioning, rational design

## Abstract

Idiopathic pulmonary fibrosis (IPF) is a fatal fibrotic interstitial lung disease (ILD) with limited therapeutic options. Autotaxin (ATX), an established drug target in IPF, is a secreted lysophospholipase D that catalyzes the extracellular production of lysophosphatidic acid (LPA), a growth-factor-like signaling phospholipid. The many pathologic effects of LPA in the lung include the co-suppression of peroxisome-proliferator-activated receptor γ (PPARγ), a therapeutic target in metabolic disorders. In this report, we introduce **EL244**, a dual ATX inhibitor and PPARγ agonist endowed with drug-like properties. Developed through repositioning, rational design, targeted synthesis, and pharmacological characterization, EL244 exhibited favorable efficacy and physicochemical profiles. Inhalation of **EL244**, which alleviates systemic toxicity concerns, attenuated bleomycin (BLM)-induced pulmonary fibrosis and restored respiratory functions; in translation, **EL244** attenuated fibrosis in human fibrotic precision-cut lung slices (PCLSs). Therefore, **EL244** emerges as a promising clinical candidate for the inhaled treatment of IPF and ILDs.

## Highlights

• *In silico* drug repurposing identifies troglitazone as an autotaxin inhibitor
• Rational design leads to **EL244**, a dual autotaxin inhibitor and PPARγ agonist
• **EL244** exhibits a favorable ADMET and PK/PD profile
• Inhaled **EL244** alleviates pulmonary fibrosis *in vivo* and prevents fibrosis in human PCLS

## Graphical abstract

**Figure:**
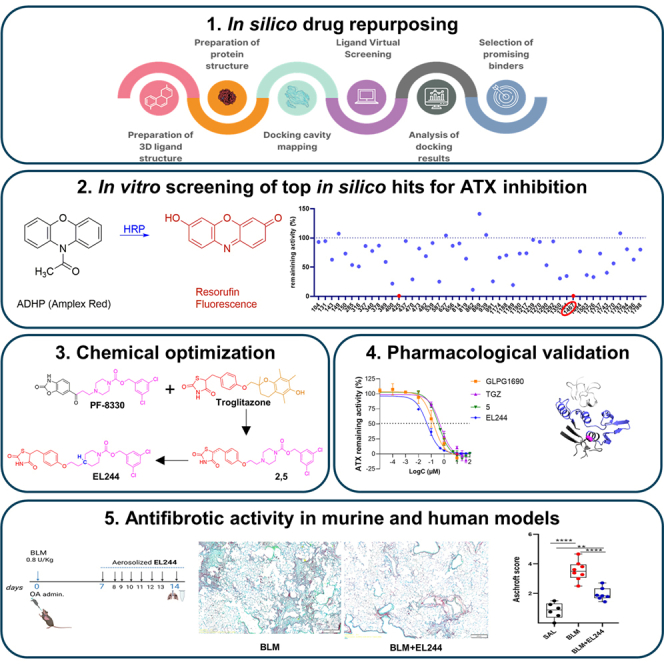

## Teaser

Matralis et al. present **EL244**, a dual ATX inhibitor/PPARγ agonist, developed through drug repositioning and rational design. Inhaled **EL244**, exhibiting a favorable physicochemical profile, attenuates bleomycin-induced pulmonary fibrosis and prevents fibrosis in human PCLS. Therefore, **EL244** emerges as a promising clinical candidate for the inhaled treatment of IPF and ILDs.

## Introduction

Fibrosis, the excessive deposition of collagen and other components of extracellular matrix, is a pathologic feature of many fibroproliferative diseases in all organs, including cancer, accounting for ∼45% of global deaths.^1^ Pulmonary fibroproliferative disorders, collectively referred to as interstitial lung diseases (ILDs), encompass a complex array with diverse prognoses and clinical behaviors, including multisystem conditions, such as systemic sclerosis (SSc-ILD) and rheumatoid arthritis (RA-ILD).^2^^,^^3^ Idiopathic pulmonary fibrosis (IPF), the most common and fatal ILD, is a chronic, progressive disease of unknown cause, with a prognosis worse than many types of cancer.^4^ While the current standard-of-care (SOC) treatments for IPF and some other ILDs, such as pirfenidone and nintedanib, slow disease progression and reduce mortality in patients who can tolerate them,^5^ they often cause troublesome side effects and have not been shown to improve quality of life in pivotal trials.^6^^,^^7^ Therefore, the treatment of IPF/ILD remains an unmet medical need.

Autotaxin (ATX) is a secreted lysophospholipase D that catalyzes the extracellular production of lysophosphatidic acid (LPA), a bioactive growth-factor-like phospholipid.^8^ Increased ATX and LPA levels have been detected in many fibroproliferative diseases,^8^ including IPF.^9^^,^^10^^,^^11^ Its genetic deletion from epithelial cells and macrophages attenuated the development of bleomycin (BLM)-induced pulmonary fibrosis in mice,^9^ indicating a pathologic role for ATX in pulmonary fibrosis and providing the proof of principle for pharmacologic targeting.^10^ Accordingly, pharmacologic ATX inhibition attenuated pulmonary fibrosis in animal models,^9^^,^^12^^,^^13^^,^^14^ thus establishing ATX as a therapeutic target in IPF and spurring the development of different ATX inhibitors.^15^^,^^16^ Although clinical trials with early ATX inhibitors have been discontinued,^17^^,^^18^ ongoing clinical trials continue to explore the therapeutic potential of ATX inhibition, using compounds with better target engagement and improved physicochemical properties.^19^^,^^20^

LPA activates its cognate GPCR receptors, widely expressed in most pulmonary cell types, to stimulate transforming growth factor beta (TGF-β) activation, vascular leak, and fibroblast accumulation,^21^^,^^22^ while many effects of LPA in different pulmonary cell types have been reported.^8^^,^^22^ Moreover, LPA has been suggested to inactivate and/or reduce the transcription of the nuclear receptor peroxisome-proliferator-activated receptor γ (PPARγ),^23^ which regulates the expression of genes involved in lipid metabolism and glucose homeostasis.^24^ PPARγ agonists (tro-, pio-, and rosiglitazones) have been used as a first-line medication for type 2 diabetes and dyslipidaemia, widespread comorbidities of IPF associated with an unfavorable prognosis.^25^ Genetic deletion of PPARγ exacerbated fibrotic responses in a murine model of sarcoidosis,^26^ and PPARγ agonists were shown to attenuate BLM-induced pulmonary fibrosis,^27^^,^^28^^,^^29^^,^^30^ suggesting a therapeutic benefit of PPARγ agonism in IPF.

In this report, *in silico* molecular docking was employed to virtually screen a library of chemical compounds of the Food and Drug Administration (FDA)-approved off-patent drugs. The identified candidates were screened using standardized *in vitro* and *ex vivo* ATX activity assays. Hit identification was followed by the rational design and synthesis of new analogs, in which the metabolically labile group of TGZ was replaced with moieties derived from the structure of known ATX inhibitors. Leads were tested as both ATX inhibitors and PPARγ agonists. The optimized lead was pharmacologically evaluated using *in vitro*, ADMET, PK/PD, and *ex vivo* assays, and its efficacy was tested in widely used animal models of type 2 diabetes and pulmonary fibrosis, as well as in human precision-cut lung slices (PCLSs). Target engagement and mode of action were investigated using hydrogen/deuterium exchange mass spectrometry (HDX/MS), tandem mass spectrometry (MS/MS), and RNA sequencing (RNA-seq).

## Results

### Repurposing TGZ as an ATX inhibitor

**Figure 1:**
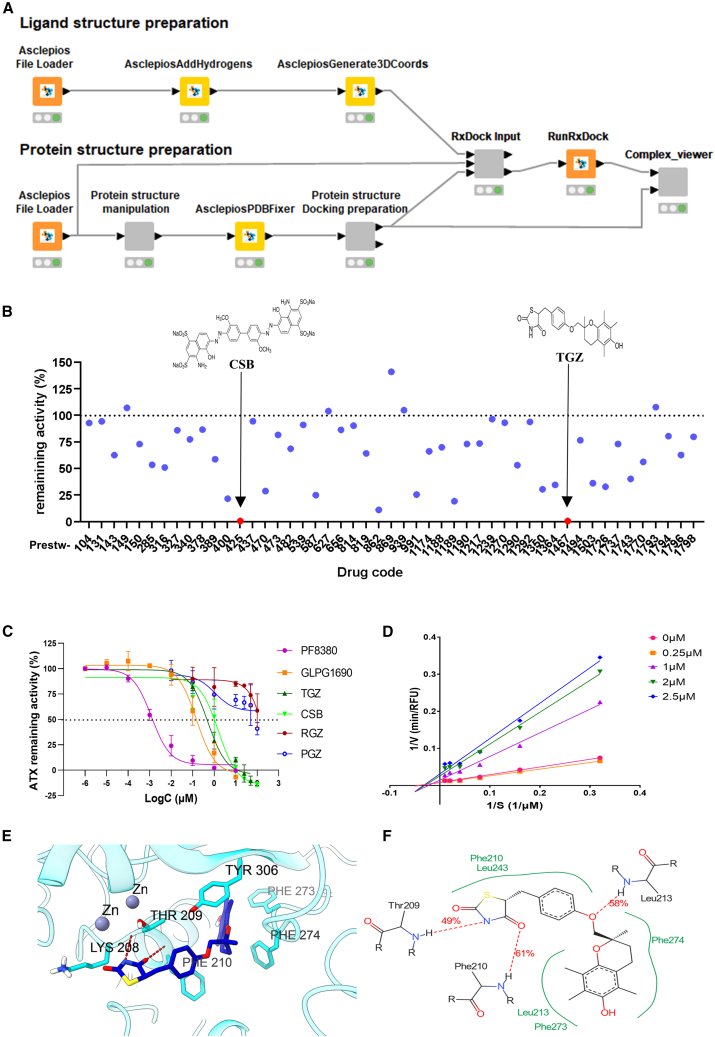
Repositioning troglitazone (TGZ) as an autotaxin (ATX) inhibitor (A) Virtual screening flow chart for the repurposing of FDA-approved drugs as potential ATX inhibitors. (B) *In vitro* screening of the *in silico* top-ranked compounds (at 100 μΜ) for their inhibitory activity against ATX. (C) Dose-response curves for the top “hits” identified, CSB and TGZ, in comparison with rosiglitazone (RGZ) and pioglitazone (PGZ), and the known ATX inhibitors PF-8380 and GLPG-1690. (D) Mode of ATX inhibition by TGZ. (E) Three- and (F) two-dimensional MD simulation representations of TGZ_1, the most favorable TGZ isomer, in complex with ATX. See also Figure S1. In (B–D), dots are indicative of three technical replicates; experiments have been conducted two times.

The repurposing of existing drugs for novel therapeutic indications has attracted substantial interest due to its capacity to expedite drug development timelines and mitigate associated costs.^31^ In this context, to potentially repurpose FDA-approved drugs as ATX inhibitors, molecular docking was employed to virtually screen the Prestwick chemical library, which comprises 1,520 off-patent drugs with known human bioavailability and safety profiles. The computational virtual screening was performed using the Enalos Asclepios KNIME drug discovery pipeline,^32^ with RxDock for high-throughput virtual screening (HTVS).^33^ HTVS (Figure 1A) was based on the docking score and the binding mode of each compound in the active site of ATX, including orientation, interactions, and size similarity with the crystallographic ATX inhibitor HA-155 (PDB ID: 2XRG), a boronic-acid-based potent ATX inhibitor.^34^ HTVS steps include the preparation of three-dimensional (3D) ligand and protein structures, docking-cavity mapping, ligand virtual screening, and selection of promising binders (Figure 1A).

The top-ranked 49 compounds (Table S1) were subsequently tested *in vitro* for their ATX inhibitory activity using the Amplex Red assay (Figure 1B),^32^^,^^35^^,^^36^ as recently published in detail.^37^ Surprisingly, the most potent ATX inhibitors were Chicago Sky Blue 6B (CSB, Prestw-425; Table S1), a diazo dye with reported anti-inflammatory properties,^38^ and TGZ (Prestw-1467; Table S1), a withdrawn first-line antidiabetic drug.^39^ Both compounds fully inhibited ATX activity at the maximum concentration tested (100 μΜ; Figure 1B; Table S1).

Dose-response curves confirmed the significant inhibitory effect against ATX (IC_50_ values of 1.60 and 0.53 μΜ for CSB and TGZ, respectively; Figure 1C), in comparison with PF-8380, the most potent *in vitro* ATX inhibitor reported,^40^ and GLPG1690, the first-in-class ATX inhibitor that entered clinical trials.^17^^,^^41^ Focusing on TGZ, mode of inhibition analysis with Lineweaver-Burk plots further revealed that TGZ is a non-competitive ATX inhibitor, suggesting that it does not bind to the catalytic site of the enzyme (Figure 1D). In agreement, molecular dynamics (MD) simulation root-mean-square deviation (RMSD) analysis (Figure S1) and molecular mechanics generalized Born and surface area continuum solvation (MM/GBSA; Table S2)^42^ suggested that TGZ binds within the deep hydrophobic pocket next to the catalytic site of ATX, in a conformation classified as a type II inhibitor. Binding free energy calculations were performed with *RR* (TGZ_1) and *SS* (TGZ_4) TGZ diastereoisomers, exhibiting the best (and very similar) calculated total binding energies to ATX (ΔG_*bind*_, Table S3). In the first case (*RR*, TGZ_1), the lipophilic 5,7,8-trimethyl-benzopyran-6-ol segment is buried close to hydrophobic residues Phe273, Phe274, and Leu213, whose backbone amino atoms form a hydrogen bond with the oxygen of anisole moiety, while the 2,4-thiazolidinedione group participates in the formation of hydrogen bonds with the amino backbone of Thr209 and Phe210 (Figures 1E and 1F), the latter of which is calculated to be the most favorable ATX amino acid toward binding (Table S2). In the other case, TGZ_4 (*SS*) positions its chromanol group near Phe274 (the most favorable residue toward binding; Table S2) and Leu213, forming a hydrogen bond. The oxygen of the anisole group is engaged in a hydrogen bond with the backbone amine of Trp275, while the 2,4-thiazolidinedione moiety forms a transient hydrogen bond with Arg244 (Figures S1B and S1C).

### Design and synthesis of dual ATX inhibitor/PPARγ agonists

**Figure 2:**
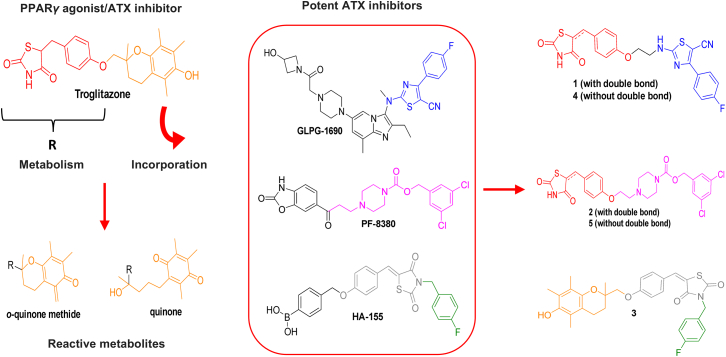
Design of TGZ derivatives 1–5 Incorporation of structural moieties of TGZ and three chemically diverse ATX inhibitors (GLPG-1690, PF-8380, and HA-155) in one structure. In **1**, **2**, **4,** and **5**, the metabolically labile chromane moiety of TGZ (in orange), which is considered responsible for TGZ-induced hepatotoxicity, was replaced by the lipophilic substituents of GLPG-1690 (highlighted in blue) and PF-8380 (highlighted in purple). The lipophilic moieties of the reference compounds highlighted in blue (GLPG-1690), purple (PF-8380), and green (HA-155) bind to the hydrophobic pocket of ATX according to X-ray crystallography studies. See also Figure S2.

TGZ is a synthetic ligand for PPARγ, the first in the thiazolidinedione (TZD) class of oral hypoglycaemic drugs.^24^ TGZ had been approved for the treatment of type 2 diabetes,^39^ but it was soon discontinued on account of hepatic toxicity.^43^ Several factors have been proposed for TGZ-induced hepatotoxicity, with the formation of reactive metabolites, particularly the oxidation of the chromane moiety of the drug’s side chain to o-quinone methide and quinone being the dominant mechanism (Figure 2).^44^ To limit hepatotoxicity, two other TZDs, rosiglitazone (RGZ) and pioglitazone (PGZ), were developed; both exhibit potent PPARγ agonism but lack hepatotoxicity.^45^ However, both RGZ and PGZ were found not to inhibit ATX enzymatic activity (Table 1; Figure 1C). The *in vitro* experimental findings, together with the cheminformatic results, which suggest the accommodation of the chromane substituent of TGZ in the hydrophobic pocket of ATX (Figures 1E, 1F, S1B, and S1C), highlight the key role of this lipophilic moiety in endowing TGZ with ATX inhibitory activity. Accordingly, the lack of potency by both RGZ and PGZ against ATX could be attributed to their polar, compared to TGZ, tail (2-methylpyridine and 5-ethylpyridine, respectively), rendering them non-tolerated in the hydrophobic pocket of ATX.

**Table 1:** *In vitro* and ADMET profile of compounds 1–5, EL244, and references

Compound	ATX IC_50_ (μΜ)	PPAR*γ* EC_50_ (μΜ) (fold activ.)/K_d_ (μΜ)	AMP (× 10^−6^) cm/s (10 μΜ)	Mouse clearance (μL/min/mg)/t_1/2_ (min) (C = 0.1 μΜ)	Cytotoxicity (HepG2) CC_50_ (μΜ)	*h*ERG IC_50_ (μΜ)
TGZ	0.53	1.30 (11 ± 1-fold)/*nd*	*nd*	*nd*	34.0	*nd*
PGZ	>100	1.00 (24 ± 3-fold)/1.5	*nd*	*nd*	*nd*	*nd*
RGZ	>100	*nd*	*nd*	*nd*	*nd*	*nd*
**1**	2.00	*nd*	*nd*	*nd*	*nd*	*nd*
**2**	2.00	*nd*	*nd*	*nd*	*nd*	*nd*
**3**	17.80	*nd*	*nd*	*nd*	*nd*	*nd*
**4**	0.94	1.00 (5.6 ± 0.5-fold)/*nd*	*nd*	*nd*	*nd*	*nd*
**5**	0.29	1.50 (9 ± 1-fold)/0.6	3.7	328.6/21	90.3	12.0
**EL244**	0.050	1.30 (17 ± 1-fold)/1.3	0.8	114.0/61	81.2	>25 (11.7% inhibition)
PF-8380	0.0012	*na*	*nd*	765.6/<10	>100	0.5
GLPG1690	0.15	*na*	*nd*	*nd*	18.5	15.0
Nintedanib	*na*	*na*	0.78	121.8/58	4.0	4.0

na, not applicable; nd, not determined.

To improve the ATX inhibitory activity of TGZ and its drug-like features, while maintaining its PPARγ agonism, which offers additional therapeutic benefits, three new molecules were initially designed (**1–3**; Figure 2) and synthesized by incorporating structural features of TGZ and known potent ATX inhibitors (GLPG1690, PF-8380, and HA-155) in one structure (Figure 2). Specifically, in compounds **1** and **2**, the metabolically labile 5,7,8-trimethyl-chroman-6-ol group of TGZ (in orange, Figure 2), which according to MD simulations appears to bind to the hydrophobic pocket of ATX (Figures 1E and 1F), was replaced by the respective lipophilic moieties of GLPG1690 (in blue, Figure 2) and PF-8380 (in purple, Figure 2), respectively, which follow a similar binding pattern.^12^^,^^41^ In contrast, compound **3** was derived by substituting the (4-(hydroxymethyl)phenyl) boronic acid group in HA-155,^46^^,^^47^ which interacts with the catalytic site of ATX, with 2-(hydroxymethyl)-2,5,7,8-tetramethylchroman-6-ol of TGZ (Figure 2). Furthermore, the reduced analogs of **1** and **2**, compounds **4** and **5**, respectively, were synthesized, aiming at exploring the impact of rigidity/flexibility on activity (Figure 2). The synthetic procedures followed for the synthesis of **1**–**5** are displayed in Figure S2 and described in detail in STAR Methods.

### Pharmacological characterization of dual ATX inhibitor/PPARγ agonist compounds

**Figure 3:**
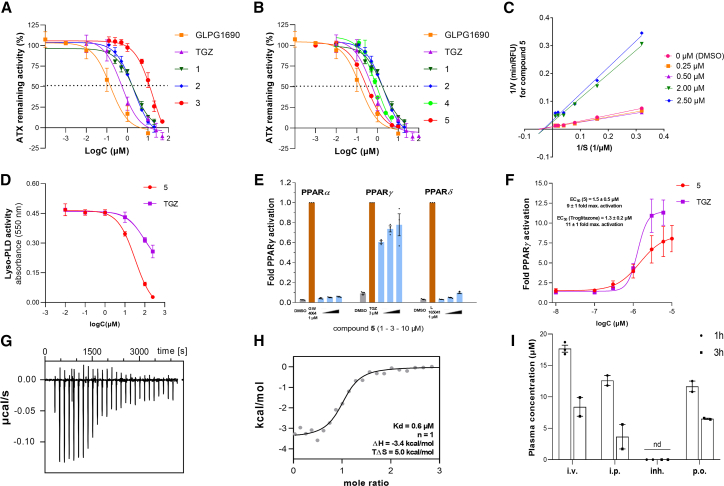
Compound 5 is a dual ATX inhibitor/PPARγ agonist (A) Dose-response curves for GLPG1690, TGZ, and compounds **1–3**. (B) Dose-response curves for compounds **1, 2, 4,** and **5**. GLPG1690 and TGZ were used as references. (C) Mode of ATX inhibition by compound **5**. (D) Dose-response curves of lyso-PLD inhibitory activity by compound **5** and TGZ in serum and in the presence of high (exogenous) LPC concentration (2 mM; TOOS assay). (E) Activity of compound **5** against PPARα, PPARγ, and PPARδ. (F) Dose-response curves for the activation of PPARγ by TGZ and compound **5**. (G) Binding of compound **5** to the PPARγ LBD was confirmed by ITC yielding a K_d_ value of 0.6 μM. Representative ITC panel showing the isotherm of the **5**-protein titration. (H) Representative panel showing fitting of the heat of binding. (I) Circulating plasma levels of compound **5** following different routes of administration (intravenous/i.v., intraperitoneal/*i.p.*, inhalation/*inh.*, and oral/*p.o.*) measured at 1 and 3 h post-administration (30 mg/kg for *i.v.*, *i.p.*, *p.o.*; 15 mg/kg for inhalation). nd, not detected, defined as plasma concentrations <50 nM. In (A–D), (F), and (H), dots are indicative of three technical replicates. Experiments were conducted two times. In (E), means of three biological replicates are shown. In (I), means of two biological replicates are shown. See also Figure S3.

Following synthesis, the ATX inhibitory activity of compounds **1–3** was tested *in vitro* (Table 1; Figure 3A). Both **1** and **2** exhibited the same activity against ATX (IC_50_ values of 2.00 μΜ), although 4-fold lower than that of TGZ (IC_50_ = 0.53 μΜ), while the inhibition offered by compound **3** was found to be very weak (IC_50_ = 17.80 μΜ). Of note, the reduction of the double bond of **1** and **2** afforded derivatives exhibiting an ATX inhibitory activity similar to TGZ (compound **4**, IC_50_ = 0.94 μΜ; Table 1) or equipotent to GLPG1690 (compound **5**, IC_50_ = 0.29 μΜ; Table 1; Figures 3B and S3A). Minimal interference of **4** and **5** on the 2nd and 3rd steps of the Amplex Red assay was observed (Figure S3B), further supporting their ATX inhibitory properties. Mode of inhibition analysis with Lineweaver-Burk plots revealed that compound **5** can bind to both the free enzyme and the enzyme-substrate complex (Figure 3C), acting as a non-competitive ATX inhibitor. Moreover, **5** was found, with the TOOS assay,^48^ to also inhibit ATX activity in serum, exhibiting a dose-dependent effect upon incubation with a high concentration of exogenous LPC (2 mM) (Figure 3D).

Compound **5** was also tested against PPARγ, as well as its variants PPARα/δ, using Gal4-hybrid receptor plasmids (pFA-CMV-hPPARα/γ/δ-LBD) transfected in HEK293 cells. The reporter system tests compound binding to the ligand-binding domain (LBD) of the canonical isoform of the respective human PPARs.^49^ Compound **5** was found to be a selective PPARγ agonist (Table 1; Figures 3E and 3F; EC_50_ 1.5 μΜ), being comparable to TGZ. It also exhibited a high binding affinity to the PPARγ LBD in isothermal titration calorimetry (ITC) (K_d_ = 0.6 μΜ; Table 1; Figures 3G and 3H), indicating that compound **5** retains its PPARγ agonist properties.

Compound **5**, exhibiting the most balanced ATX inhibitor/PPARγ agonist profile, was then evaluated *in vitro* in terms of representative ADMET properties potentially affecting the *in vivo* pharmacokinetic (PK) profile of a bioactive molecule (Table 1). It exhibited increased cell membrane permeability and a moderate metabolic clearance, without displaying significant cytotoxicity (EC_50_ = 90.3 μΜ; Table 1). In addition, a weak inhibitory activity against the cardiac potassium ion channel *h*ERG (*human* Ether-à-go-go-Related Gene) was exhibited (IC_50_ = 12 μΜ), which was much lower than that of PF-8380 (IC_50_ = 0.5 μΜ), was similar to that of GLPG1690 (IC_50_ = 15 μΜ), and was lower than that of the SOC nintedanib (IC_50_ = 4 μΜ) (Table 1).

Fast-track PK analysis of compound **5**, specifically its plasma exposure, was measured after different routes of administration (intravenous [*i.v.*], intraperitoneal [*i.p.*], inhalation, and *per os*). The plasma concentrations of **5** after *i.p.* and *per os* administration, and at 1 and 3 h post-dose, were comparable to the respective *i.v.* concentrations used as a reference (100% absorption) (Figure 3I), indicating favorable compound absorption into the circulation. Notably, the direct administration of **5** to the lung via inhalation resulted in prolonged exposure and retention in the tissue, with no detectable compound in the circulation even 3 hours post-dose (Figure 3I), suggesting targeted delivery of compound **5** to the respiratory tract.

Given the established role of ATX in the pathogenesis of pulmonary fibrosis^9^^,^^10^ and the suggested involvement of PPARγ,^27^^,^^50^ the efficacy of **5** to inhibit pulmonary fibrosis was then tested. Compound **5** was first tested on mouse PCLSs, living lung slices isolated from BLM-induced fibrotic mouse lung tissue, as recently published in detail.^51^ PCLSs, a bridge between traditional *in vitro* cell cultures and *in vivo* animal models, have emerged as a valuable, medium-throughput pre-clinical platform to test new pharmacological compounds.^52^ Compound **5**, at 30 μΜ and after 72 h of incubation, improved the lung architecture compared to the BLM group, with much fewer fibrotic lesions being observed (Figures S4A and S4B), decreasing at the same time the *m*RNA expression levels of the profibrotic gene expression markers collagen 1α1 (*Col1a1*) and fibronectin 1 (*Fn1*) (Figures S4C and S4D).

Compound **5** was then evaluated in the BLM-induced pulmonary fibrosis model, the most widely used animal model for pulmonary fibrosis.^53^^,^^54^ BLM (0.8 U/kg) was administered via oropharyngeal administration (OA) to littermate C57Bl/6 mice, as described previously,^54^^,^^55^ as analyzed in detail (protocols.io), and as summarized graphically (Figure S4E). Given the favorable PK profile upon inhalation (Figure 3I), the therapeutic potential of **5** was evaluated following its inhaled administration (15 mg/kg) twice daily (b.i.d.) in a therapeutic mode, starting the administration 7 days post-BLM (Figure S4E). The aerosolized delivery of **5** efficiently attenuated the BLM-induced impairment of respiratory functions, as indicated by mean static lung compliance (Cst), mean respiratory system compliance (Crs), and total mean lung capacity (A) values (Figures S4F–S4H). Accordingly, **5** reduced vascular leak and pulmonary edema as indicated by the decreased total protein levels in the bronchoalveolar lavage fluid (BALF) (Figure S4I) and decreased inflammatory cells in the BALF (Figure S4J). Although the decrease of BALF soluble collagen (Figure S4K) and lung tissue *Col1a1* mRNA levels (Figure S4L) did not reach statistical significance following compound **5** administration, histological analysis revealed fewer fibrotic regions and decreased collagen deposition as evaluated with H&E and fast green/Sirius red staining (Figures S4M and S4N). Therefore, the favorable PK profile and promising efficacy of **5** suggest further preclinical development and optimization.

### Development of EL244, the first-in-class dual ATX inhibitor/PPARγ agonist

**Figure 4:**
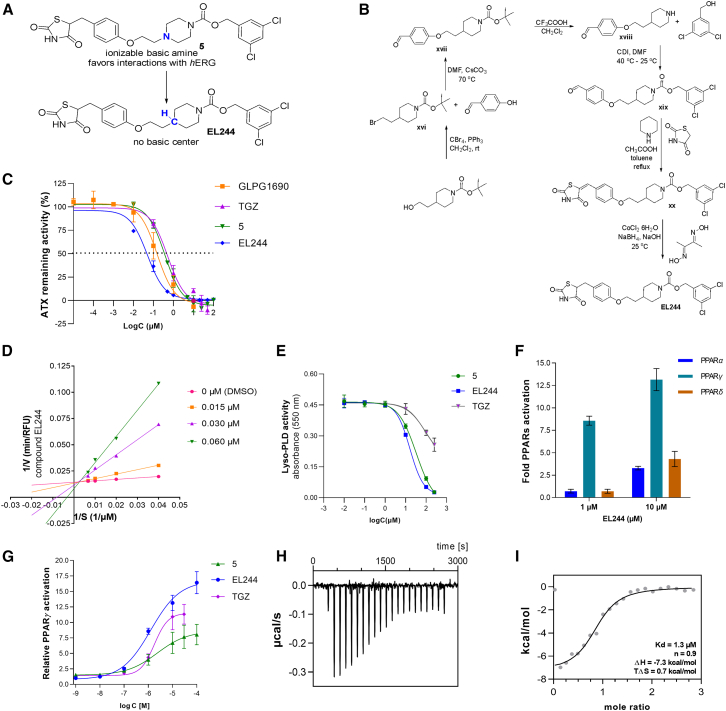
Synthesis and *in vitro* and *ex vivo* activity of **EL244** (A) Targeted design toward decreasing *h*ERG interactions. (B) Synthetic procedure followed for the synthesis of **EL244**. (C) Dose-response curves for GLPG1690, TGZ, **5,** and **EL244** against ATX *in vitro* (Amplex Red assay). (D) Mode of inhibition of ATX by compound **EL244**. (E) *Ex vivo* (lyso-PLD activity assay), in serum and in the presence of 2 mM exogenous LPC for TGZ, **5**, and **EL244**. (F) Selectivity of **EL244** to PPARγ. (G) Dose-response curves for the activation of PPARγ by TGZ, **5**, and **EL244**. (H) Binding of **EL244** to the PPARγ LBD was confirmed by ITC. Representative ITC panel showing the isotherm of the **ΕL244**-protein titration. (I) Representative panel showing fitting of the heat of binding. In (C), (D), (E), (G), and (I), dots are indicative of three technical replicates; experiments were conducted twice. In (F), means of three biological replicates are shown. See also Figure S4.

Based on the ADMET profile of **5** (Table 1), we focused on its *h*ERG inhibition, which, although moderate to weak, could lead to potential side effects related to cardiovascular toxicity. Importantly, the *h*ERG toxicity evaluation early in the preclinical setting is strongly recommended by FDA and European Medicines Agency (EMA), while cardiovascular assessment constitutes a pivotal point for obtaining investigational new drug (IND) status.^56^ Accordingly, to avoid *h*ERG interactions, the piperazine group of **5** was replaced by the piperidine one (Figure 4A), on the grounds that highly basic (ionizable) amine motifs are well recognized and accommodated in the negative electrostatic potential located in *h*ERG’s central cavity.^57^^,^^58^ It should be noted that the most potent ATX inhibitor currently available, PF-8380 (Figure 2), has been found to significantly inhibit *h*ERG (Table 1), an effect attributed to the basic piperazine group.^59^ Furthermore, the former clinical candidate GLPG1690 (Figure 2), exhibiting a moderate-to-weak inhibitory activity against *h*ERG (Table 1)^12^ originated from reducing the basicity of a precursor molecule of the same series, which exerts a much more potent *h*ERG inhibition.^12^^,^^41^

The synthetic route used to produce **EL244** is illustrated in Figure 4B and described in detail in the STAR Methods. Remarkably, this minor structural modification implemented on **5** proved advantageous in reducing its overall *h*ERG binding affinity, since **EL244** inhibited the cardiac potassium ion channel in a very weak fashion (approximately 12% at 25 μΜ; Table 1). Furthermore, the cytotoxicity of **EL244** in human HepG2 hepatocytes was very weak (EC_50_ = 81.2 μΜ; Table 1), similar to that of compound **5**, and much lower than those of the reference compounds TGZ, GLPG1690, and nintedanib (Table 1), indicating low risk for liver toxicity. Notably, **EL244** exhibited a much lower metabolic clearance rate compared to that of **5** (Table 1), thus identifying the tertiary amine of the piperazinyl group of compound **5** as a major metabolic “soft spot.”

Importantly, this modification conferred a 5-, 10-, and 3-fold better ATX inhibitory effect *in vitro* compared to that of **5**, TGZ, and GLPG1690, respectively (Table 1; Figure 4C). Mode of inhibition analysis (Figure 4D) indicated a competitive inhibition for **EL244**, providing evidence that the compound binds only to the free enzyme and in such a way as to compete with LPC binding to ATX. Improved lyso-PLD inhibition *ex vivo* for **EL244** was also observed (Figure 4E), further confirming **EL244’s** potency in inhibiting ATX.

**EL244** activated PPARγ selectively (Figure 4F) and more efficiently than its precursor compound **5** and TGZ (Figure 4G) and similar to PGZ at the same EC_50_ concentration (Table 1). Of note, it can be inferred by the ITC studies (Figures 4H and 4I) that although the binding of both **5** and **EL244** to the LBD of PPARγ is exothermic, with their ΔG and Kd values being close, each compound exhibits different thermodynamics. In particular, the protonated piperazinyl-carbamate side chain in **5** seems to favor an entropy-driven binding (Figures 3G and 3H), while **EL244**, having the neutral piperidinyl-carbamate side chain, binds in an enthalpy-driven manner to the PPARγ-LBD (Figures 4H and 4I).

### EL244 is a type IV ATX inhibitor

**Figure 5:**
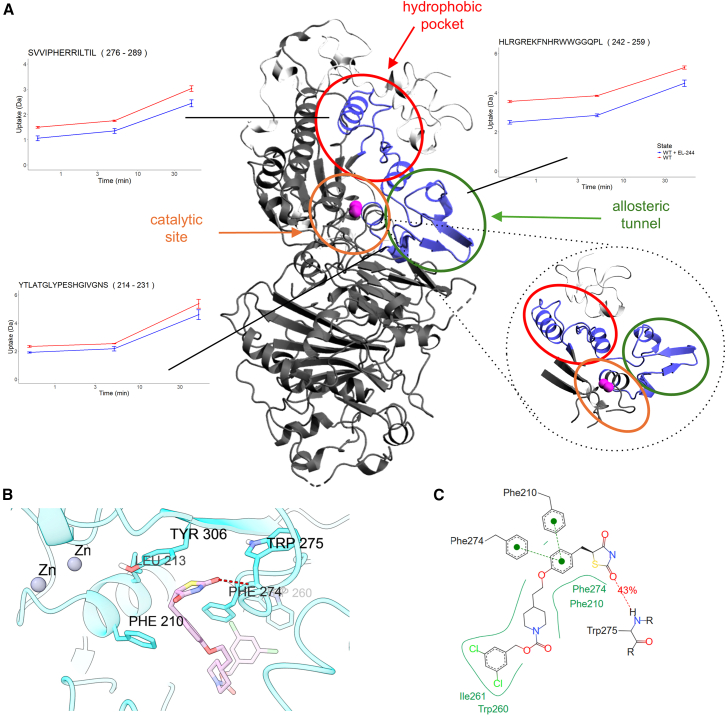
HDX-MS and MD simulations identify **EL244** as a type IV ATX inhibitor (A) HDX-MS significant differences after binding with **EL244** mapped onto the ATX crystal structure (2XR9) and representative uptake plots of the differences. Blue represents regions with increased protection upon binding, gray regions indicate no differences, and white indicates regions with no coverage. Purple spheres correspond to zinc ions in the crystal structure (catalytic cofactors). Close-up region shows the proposed binding region identified by HDX-MS. The three binding sites of ATX are displayed with circles, and the region protected by both analogues is displayed with blue. (B) Three- and (C) two-dimensional MD simulation representations of the *S*-isomer of **EL244** (EL244_2) in complex with ATX. See also Figure S5.

To gain structural insights into the binding mode of **EL244** to ATX, we employed HDX/MS in conjunction with MD simulations. Optimized digestion conditions yielded 310 peptides that covered 84.5% of the ATX sequence, with a redundancy of 5.62 (Table S4 and Data S1; Figure S5A). Statistical analysis (α = 0.01) using a hybrid significance test of the differential HDX-MS data^60^ (Figure S5B) identified several overlapping peptides (242–259, 276–289, and 214–231) as protected at different time points (0.5, 5, and 50 min) when bound to **EL244** (Figure S5C), covering the region 214–289. Mapping **EL244-**protected ATX fragments onto the crystal structure of ATX (2XR9)^61^ (Figure 5A) revealed that **EL244** binds to the hydrophobic pocket and the allosteric tunnel of ATX.^16^

In alignment with the HDX-MS results, MD simulations with R- (EL244_1) and *S*-enantiomers (EL244_2) of **EL244** indicated that only the binding of *S*-enantiomer is compatible with a type IV inhibition (Table S3; Figures 5B and 5C). Accordingly, **EL244**_2 maintains a type-IV-inhibitor-binding mode, with its thiazolidine-2,4-dione moiety shifted toward the allosteric tunnel, forming a hydrogen bond with the backbone amino group of Trp275 (Figures 5B and 5C), mediated by water for approximately 53% of the simulation (Table S5). The anisole fragment is oriented toward Phe274, calculated as the most favorable residue (Table S2), and Phe210, while the dichlorobenzene group extends further inside the allosteric hydrophobic tunnel, toward Trp260 and Ile261 (Figures 5B and 5C). Therefore, HDX/MS and MD analysis classifies **EL244** as a type IV ATX inhibitor.

### EL244 is a potent and physiologically relevant PPARγ agonist

Extending reporter and ITC assays, MD analysis of **EL244** isomers with PPARγ indicated that all complexes are structurally stable (Figure S6A; Table S6) and that Cys285 is central in the binding of **EL244** isomers to the LBD of PPARγ (Figures S6B–S6E). The *S*-enantiomer of **EL244** (EL244_2; Table S3) is characterized by favorable interactions and a hydrogen bond network formed between the 2,4-thiazolidinedione pharmacophore and Ser289, His323, His449, and Tyr473 residues (Figures S6B and S6C; Table S6), contrary to its *R*-counterpart **EL244**_1 (Figures S6D and S6E; Tables S3, S5, and S6), thus highlighting the importance of chirality in ligand recognition and protein function.^62^ It is worth mentioning that the latter hydrogen bond network is totally preserved in the active conformations (*S*-enantiomers) of the reference compounds (PGZ_2, RGZ_2, and TGZ_3; Table S3) within PPARγ-LBD (Figures S6F–S6Κ; Table S6), thus representing the archetypal engagement of effective PPARγ agonists, which is preserved in **EL244**, further supporting efficient PPARγ agonism by **EL244**.

**Figure 6:**
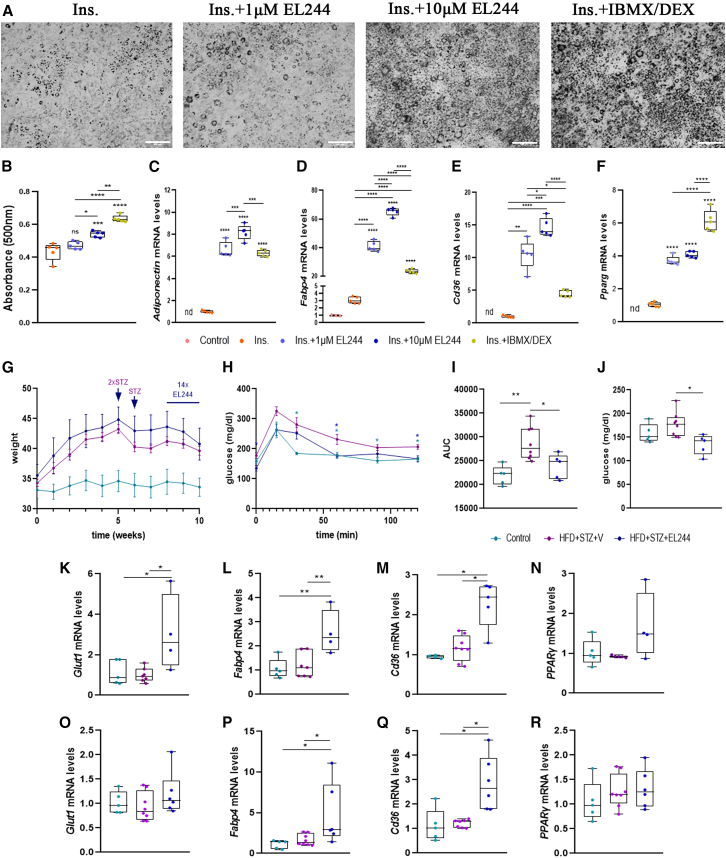
**EL244** is a potent PPARγ agonist with therapeutic potential In (A–F), **EL244** ability to differentiate 3T3-L1 fibroblasts to adipocytes in the presence of insulin was tested. (A) Oil red O staining of lipid droplets indicative of adipocyte differentiation. The cocktail containing insulin (Ins.), dexamethazone (DEX), and 3-isobutyl-1-methylxanthine (IBMX) was used as a positive control. Scale bars, 100 μm. (B) Absorbance of oil red O staining in cells (*n* = 5, 5, 5, 5). (C–E) mRNA levels of PPARγ target genes *Adiponectin* (C), *Fabp4* (D), and *Cd36* (E) in 3T3-L1 or the differentiated adipocytes. mRNA expression was interrogated with RT-qPCR; values were normalized to the expression of *B2m* and presented as fold change over control (*n* = 3, 5, 5, 5, 5). (F) mRNA levels of *PPARγ* in 3T3-L1 or the differentiated adipocytes. mRNA expression was interrogated with RT-qPCR; values were normalized to the expression of *B2m* and presented as fold change over control (*n* = 3, 5, 5, 5, 5). In (G–R), C57bl/6 male mice were treated with high-fat diet (HFD) for 10 weeks and three injections of streptozotocin (STZ, 40mg/kg). In the last 2 weeks of the HFD treatment, a group of mice was treated daily with **EL244** (50 mg/kg). (G) Weight curves of the different groups of mice during the HFD, STZ, and **EL244** administration. (H) Oral glucose tolerance test (oGTT) after oral administration of a 10% glucose solution (1 g/kg). (I) Insulin resistance as assessed by the area under curve (AUC) of oGTT (*n* = 5, 8, 5). (J) Glucose levels in the beginning of the oGTT (*n* = 5, 8, 5). (K–R) mRNA levels of *PPARγ* and PPARγ target genes in the adipose tissue (K–N) and liver (O–R) from mice subjected to HFD, STZ, and **EL244** treatment. mRNA expression was interrogated with RT-qPCR; values were normalized to the expression of *B2m* and presented as fold change over control. (K) *Glut1* (*n* = 5, 7, 4), (L) *Fabp4* (*n* = 5, 7, 4), (M) *Cd36* (*n* = 4, 8, 5), and (N) *PPARγ* (*n* = 5, 5, 4) mRNA levels in adipose tissue. (O) *Glut1* (*n* = 5, 8, 6), (P) *Fabp4* (*n* = 5, 8, 6), (Q) *Cd36* (*n* = 5, 7, 6), and (R) *PPARγ* (*n* = 5, 8, 6) mRNA levels in liver. Data in box and whiskers include the median (line), interquartile range (box), and minimum and maximum range (tails). Each dot represents a biological replicate. Following normality testing, statistical significance was assessed with one-way ANOVA and Tukey’s post-hoc test (B–D), (F), (I–L), (N), (O), (P), and (R) or Welch ANOVA and Games-Howell’s post-hoc test (E), (M), and (Q). ∗*p* < 0.05, ∗∗*p* < 0.01, ∗∗∗*p* < 0.001, ∗∗∗∗*p* < 0.0001, respectively. See also Figure S6.

To further prove **EL244**’s potent PPARγ agonism functionally, its ability to differentiate 3T3-L1 fibroblasts to adipocytes in the presence of insulin was then tested, in comparison to dexamethasone (DEX) and 3-isobutyl-1-methylxanthine (IBMX), in a widely used adipocyte differentiation assay.^63^ **EL244** promoted adipogenesis dose responsively, as compared with IBMX/DEX, as evident by the formation of oil-red-O-detected lipid droplets (Figures 6A and 6B). **EL244** induced the expression of several PPARγ target genes (Figures 6C–6E), such as adiponectin (*Adipoq*), fatty acid binding protein 4 (*Fabp4*), and *CD36*, as well as *PPARγ* itself (Figure 6F), in a more efficient manner than IBMX/DEX.

Since PPARγ agonists are known antidiabetic drugs, we then examined **EL244**’s efficacy in a widely used type 2 diabetes model.^64^^,^^65^^,^^66^ One-year-old male mice were fed a high-fat diet (HFD) for 10 weeks and were injected with streptozotocin (STZ) at predetermined time points (Figure 6G). Following an oral glucose tolerance test (oGTT) at 8 weeks indicating prediabetes, a subset of prediabetic mice was injected intraperitoneally with **EL244** (50 mg/kg) daily for the final 2 weeks, thereby allowing the full development of diabetes in control mice (Figure 6G). In oGTT analysis, the main readout of the diabetic model, mice treated with **EL244** showed decreased insulin resistance (Figures 6H and 6I) and lower glucose levels (Figure 6J), as previously observed with other clinically used PPARγ agonists, e.g., TGZ.^67^^,^^68^ **EL244** stimulated the expression of several PPARγ target genes, tissue-specifically, in both adipose and liver tissue (Figure 6K–6R). Although further studies will be needed to examine the therapeutic potential of **EL244** in metabolic disorders, these results establish **EL244** as an *in vivo* efficacious potent PPARγ agonist.

### EL244 attenuates pulmonary fibrosis

**Figure 7:**
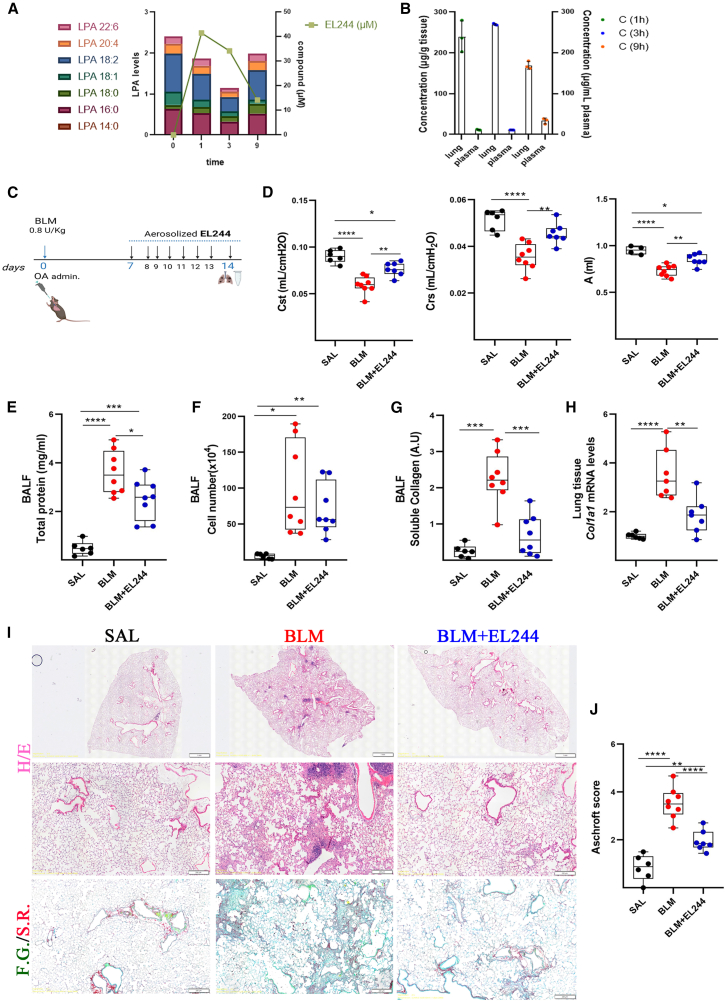
Inhaled therapeutic **EL244** administration attenuates BLM-induced pulmonary fibrosis (A and B) PK/PD analysis of **EL244**. (A) **EL244** concentration and total LPA levels in the plasma of WT mice at 0, 1, 3, and 9 h after its *i.p.* administration (30 mg/kg). (B) **EL244** concentrations in lung tissue and plasma, at 1, 3 and 9 h after its inhaled administration (15 mg/kg). (C–J) **EL244** was administered via inhalation at 15 mg/Kg, once daily, for 8 consecutive days (from 7th to 14th day) post-BLM administration. The compound was dissolved in 15% Kolliphor in saline. The vehicle (15% Kolliphor in saline) was administered to both the SAL and BLM control groups. (C) Schematic representation of drug administration. (D) Respiratory mechanics assessed using the FlexiVent system (Cst, Crs *n* = 6, 8, 7; A *n* = 4, 8, 7). Parameters include mean respiratory system compliance (Crs), mean total lung capacity (A), and mean static lung compliance (Cst). (E) Total protein concentration in BALFs, as determined with the Bradford assay (*n* = 6, 8, 8). (F) Inflammatory cell numbers in BALFs, as counted with a hematocytometer (*n* = 6, 8, 8). (G) Soluble collagen levels in the BALFs were detected with the direct red assay (*n* = 6, 8, 8). (H) *Col1a1* mRNA expression was interrogated with RT-qPCR (*n* = 6, 8, 8); values were normalized to the expression of *B2m* and presented as fold change over control. (I) Representative images from lung sections of murine lungs of the indicated treatment groups, stained with H&E and fast green/Sirius red (F.G/S.R; green/red). Scale bars: 1 mm and 200 μm. (J) Quantification of fibrosis severity in H&E-stained lung sections via Ashcroft scoring (*n* = 6, 8, 7). Data in box and whiskers include the median (line), interquartile range (box), and minimum and maximum range (tails). In (B), data are presented as means ± SEM. Each dot represents a biological replicate. Following normality testing, statistical significance was assessed with one-way ANOVA and Tukey’s post-hoc test (D, E, G, and H) or with Welch ANOVA and the Games-Howell post-hoc test (F and J); ∗*p* < 0.05, ∗∗*p* < 0.01, ∗∗∗*p* < 0.001, ∗∗∗∗*p* < 0.0001 respectively. See also Figure S7.

Fast-track PK analysis of **EL244** upon *i.p.* administration (30 mg/Kg) indicated that the compound reaches a 40 μM concentration in the plasma in 1 hour, and its plasma levels are retained up to 3 h (∼35 μΜ), while it can still be detected at lower concentrations (15 μM) even 9 h post-dose (Figure 7A). Pharmacodynamic (PD) analysis of **EL244**, as measured by the reduction of plasma LPA levels using MS/MS, revealed a maximal effect at 3 h post-administration (Figure 7A). Interestingly, PK analysis of **EL244** after inhaled administration (15 mg/kg) indicated that the compound is highly retained in the lung, while its clearance rate from the lung and its absorption in the systemic circulation were found to be low (Figure 7B), suggesting targeted delivery of **EL244** directly to the lung. Therefore, considering the therapeutic benefits of inhaled administration, the efficacy of **EL244** was evaluated upon its inhaled administration in the BLM-induced model of pulmonary fibrosis,^53^^,^^54^^,^^69^ in prophylactic and therapeutic modes (15 mg/kg; once daily [o.d.]).

In the prophylactic mode, **EL244** was administered for 15 consecutive days, starting one day before BLM administration (Figure S7A). **EL244** significantly improved respiratory functions (Figures S7B–S7D) and markedly reduced BLM-induced pulmonary edema and inflammation (Figures S7E and S7F). Soluble collagen levels in BALF and *Col1a1* mRNA expression in lung tissue were also significantly reduced (Figures S7G and S7H). Histological analysis confirmed attenuated collagen deposition and reduced fibrotic regions (Figures S7I and S7J). The 2 weeks of inhaled administration of **EL244** did not cause any appreciable toxicity in the liver, as indicated by the ALT/AST levels (Figure S7K), and as expected by the limited absorption of **EL244** in the systemic circulation (Figure 7B).

In the therapeutic mode, **EL244** was administered o.d. for seven consecutive days, starting on day 7 post-BLM administration, when inflammation begins to diminish and fibrotic regions appear (Figure 7C).^54^^,^^70^ **EL244** significantly restored all assessed respiratory functions (Figure 7D) and reduced vascular permeability (Figure 7E). The **EL244** effects on inflammation did not reach statistical significance (Figure 7F), as opposed to compound **5**, which, however, was administered twice daily (bid; Figure S7F), and as opposed to the prophylactic delivery, where **EL244** was administered before the onset of pulmonary inflammation.^54^^,^^70^ Importantly, **EL244** demonstrated strong anti-fibrotic effects, significantly reducing soluble collagen in BALF and *Col1a1* expression in lung tissue (Figures 7G and 7H). Histopathological analysis revealed reduced collagen deposition and fewer and smaller fibrotic regions (Figures 7I and 7J), establishing **EL244** as a potent anti-fibrotic compound.

To translate findings to humans and assess applicability to the human disease, TGF-β-induced human lung fibroblasts were treated with non-toxic concentrations of **EL244**, as determined by the MTT assay (Figure S8A). As expected for a PPARγ agonist,^71^ **EL244** reduced TGF-β-induced mitochondrial activation, as shown by the mitotracker assay (Figure S8B). However, the effects of ATX/LPA and PPARγ, and their interplay, on mitochondrial homeostasis in both fibroblasts and macrophages remain unexplored. Moreover, **EL244** suppressed the expression of *Col1a1* and *Acta2* (aSMA) at both the mRNA and protein levels (Figures S8C–S8G), while simultaneously stimulating the expression of PPARγ target genes (Figures S8H–S8I) and PPARγ itself (Figure S8J) in the same samples.

More importantly, **EL244** was tested in human PCLS^72^ prepared from tumor-free lung tissue obtained during surgical resection of a 71-year-old male patient with pulmonary adenocarcinoma; tissue/cell viability was assessed using the MTT assay (Figure S8K). Fibrosis was induced by a pro-fibrotic cocktail, following a well-established protocol.^73^ **EL244** prevented and/or attenuated the development of fibrosis, as shown with histology (Figure S8L) and the downregulation of the mRNA expression of *Col1a1* and *Acta2* (Figures S8M and S8N). **EL244** treatment upregulated the expression of PPARγ target genes (Figures S8O and S8P), suggesting that the efficacy of **EL244** may be partly attributable to PPARγ agonism. Therefore, **EL244** is a promising anti-fibrotic compound for the treatment of pulmonary fibrosis.

### EL244 mode of action

To verify ATX engagement by **EL244** and the diminished production of LPA, the enzymatic product of ATX that mediates its pathological effects, LPA levels were measured using MS/MS in the plasma and BALF of mice after BLM administration and inhaled administration of **EL244**. All LPA species, except for 18:0, were found to be reduced in the BALF, including the most abundant species 16:0 and 18:2 (Figure S9A). As a result, total LPA content in the BALFs was found reduced (Figure S9B). Consistent with the high absorption of **EL244** in the lung (Figure 7B), no modulation of plasma LPA levels was detected (Figures S9C and S9D), indicating that the effects of **EL244** are localized in the lung.

To examine PPARγ engagement by **EL244** in the lung, we performed RT-qPCR in the lung tissue on the same PPARγ target genes that were found to be induced by **EL244** in 3T3-L1 fibroblasts (Figures 6C–6F) and diabetic adipose tissue (Figures 6O–6R) and observed marginal increases (Figure S9E); PPARγ was found to be downregulated upon fibrosis, as previously indicated for IPF macrophages.^74^ However, PPARγ effects in fibroblasts and macrophages may be masked in whole-lung tissue. In agreement, **EL244** promoted the expression of PPARγ target genes in TGF-β-induced human normal lung fibroblasts (Figures S8H–S8J), as previously reported for other PPARγ agonists.^27^ The same genes were also found to be upregulated upon **EL244** treatment of hPCLS (Figures S8O and S8P), further supporting that the anti-fibrotic efficacy of **EL244** can be partly attributable to PPARγ agonism.

To further dissect the therapeutic effects of **EL244**, the gene expression profiles of healthy lung tissue (wt SAL), fibrotic tissue post-BLM (wt BLM), and post-BLM upon **EL244** administration were analyzed using RNA sequencing; multidimensional scaling (MDS) of samples indicated proper sample clustering (Figure S9F). BLM-induced pulmonary fibrosis was found to deregulate the expression of 3,086 genes in the lungs (differentially expressed genes [DEGs]) in comparison with saline-treated mice (SAL) (Figure S9G; Data S2), which include many well-known pro-fibrotic DEGs (such as, most notably *Col1a1*, *Tnc*, *Timp1*, *Arg1*, *Cthrc1*, *Eln*, *Fn1*, *Spp1*, and *Trem2*; Figure S9G). Inhaled administration of **EL244** in BLM-treated mice resulted in the deregulation of only 741 genes in comparison with the BLM-treated mice, including the downregulation of many of the identified pro-fibrotic BLM-DEGs (Figure S9H; Data S3). Remarkably, most **EL244**-deregulated DEGs (646) are a subset of BLM-induced DEGs (Figure S9I). Among these, 363 BLM-induced DEGs were suppressed by **EL244** treatment (Q3) (Data S4 and S5; Figure S9J); the detected expression changes of four selected DEGs were confirmed with RT-qPCR (Figure S9K).

Gene Ontology (GO) analysis of the Biological Processes (BP) of Q3 DEGs indicated that **EL244** administration downregulated genes involved in the cell cycle and mitosis (Figure S9L), consistent with the well-known mitogenic properties of LPA,^8^^,^^22^ while PPARγ agonism has been suggested to induce cell-cycle arrest via Cyclin D^27^ and suppress cell proliferation.^27^^,^^75^ Interestingly, three of the BLM-induced genes (Figure S9G; Data S2) that were suppressed by **EL244** (Figure S9H; Data S3) have been reported to be regulated by PPARγ: TIMP-1, crucial for extracellular matrix (ECM) homeostasis and highly implicated in IPF pathogenesis, has been suggested to be modulated by PPARγ, depending on the cellular and tissue context. Arginase-1 (Arg-1), increasingly implicated as a metabolic driver of collagen production^76^ and a classic marker and effector enzyme of interleukin (IL)-4-/IL-13-driven alternatively activated (M2) macrophages in mice,^77^ has been shown to be regulated by PPARγ.^78^^,^^79^ Secreted phosphoprotein 1 (Spp1; Osteopontin), emerging as a central pro-fibrotic mediator produced mainly by a distinct SPP1^+^ pathologic macrophage subpopulation,^80^^,^^81^ has also been proposed to be regulated by PPARγ.^80^^,^^82^ Triggering receptor expressed on myeloid cells 2 (Trem 2) marks and functionally sustains a pro-fibrotic monocyte-derived lipid alveolar macrophage subset in IPF,^83^^,^^84^ suggesting Trem2 as a major macrophage sensor of lipids,^84^ whose expression is thought to be regulated by PPARγ.^71^ Respectively, the most statistically enriched molecular function (MF) of these downregulated DEGs concerned the ECM (Figure S9M), in agreement with the observed attenuation of BLM-induced pulmonary fibrosis by **EL244**.

## Discussion

The establishment of ATX as a therapeutic target in IPF has led to the ISABELA clinical trials with GLPG1690, among the largest IPF clinical studies of the last decade. However, the trials were discontinued due to a low risk-to-benefit ratio.^17^^,^^18^ Moreover, the COVID-19 pandemic affected the participation and compliance of patients and unexpectedly stimulated mortality in the GLPG1690 group.^17^ However, the complex effects of ATX/LPA on immune regulation,^85^ especially amid the cytokine storm in COVID-19,^86^ remain unresolved. More importantly, the inhaled administration of ATX inhibitors, as suggested here for **EL244**, would alleviate systemic effects, including potential impacts on T cell homeostasis. Moreover, inhaled administration of **EL244**, restricting its effects in the lung, is predicted to avoid drug-drug interactions with the SOC compounds, as previously shown with GLPG1690 and nintedanib.^87^

LPA mediates most of the pathologic properties of ATX, stimulating TGF-β activation, endothelial permeability, and fibroblast accumulation.^21^^,^^22^ Many effects of LPA in different pulmonary cell types, including immune cell types, have been reported.^8^^,^^22^ Inhaled **EL244** decreased LPA levels in the BALF but not in the plasma, indicating that the **EL244**-mediated effects were confined to the lung. Moreover, **EL244** decreased all LPA species and total LPA levels, much like the genetic deletion of ATX,^88^ while increased levels of most LPA species (16:0, 16:1, 18:1, 18:2, and 20:4) have been reported in patients suffering from IPF.^11^ Therefore, **EL244** exhibits efficient and physiologically relevant engagement of ATX, an established therapeutic target.

Beyond its ATX inhibitory properties, **EL244** is also a potent and selective PPARγ agonist, as shown here by well-established reporter assays, ITC, and MD analyses. More importantly, **EL244** was shown to stimulate fibroblast transdifferentiation into adipocytes, consistent with the established role of PPARγ as a master regulator of adipogenesis, and to lower glucose levels in diabetic mice, thereby establishing **EL244** as a physiologically relevant, potent PPARγ agonist. In the mouse lung, where a role for PPARγ in the metabolic regulation of fibroblasts and macrophages has been suggested, **EL244**’s effect on PPARγ target gene expression was minimal, possibly masked by contributions from other cell types. However, the attenuation of fibrosis and fibrotic gene expression in human fibrotic PCLS by **EL244** was accompanied by induction of PPARγ target gene expression, possibly due to the lower cell complexity and the higher exposure.

TGF-β, the main pro-fibrotic factor, has been shown to suppress PPARγ; conversely, PPARγ activation has been demonstrated to suppress TGFβ-induced mitochondrial activation.^89^ Interestingly, it has been more recently suggested that the pathogenesis of pulmonary fibrosis involves TGF-β-induced differentiation of lipofibroblasts, a novel subset of pulmonary fibroblasts, into myofibroblasts and that activation of PPARγ inhibits this differentiation, suppressing the development of pulmonary fibrosis.^71^ Moreover, PPARγ activation in macrophages, cells central to pulmonary immunometabolism, has been suggested to directly regulate several macrophage functions, including differentiation from monocytes and polarization.^90^ Intriguingly, LPA has been suggested to suppress PPARγ,^23^ although the underlying mechanisms, possibly including the Wnt pathway,^91^ remain understudied, so does the involvement of PPARγ in pulmonary fibrosis. However, PPARγ agonists have been reported to attenuate BLM-induced pulmonary fibrosis,^27^ suggesting that a part of the therapeutic efficacy of **EL244** can be attributed to PPARγ agonism.

**EL244**, in addition to exhibiting dual potent ATX inhibitory activity and PPARγ agonism, and efficacy in attenuating pulmonary fibrosis, also showed a favorable basic ADMET profile (intrinsic clearance, hepatotoxicity, and cardiotoxicity), outperforming reference compounds (Table 1). **EL244** is further differentiated from other suggested anti-fibrotic compounds by its route of administration. Inhaled therapies have long been a cornerstone in the management of obstructive lung diseases due to their ability to deliver medication directly to the active disease site, thereby increasing drug tissue concentration and efficacy while reducing the dosage.^92^ Moreover, inhalation bypasses the intracellular and extracellular drug-metabolizing enzymes in the liver and the gastrointestinal tract, while minimizing potential systemic side effects.^92^ Despite these clear advantages, their application in IPF has been relatively underexplored due to higher costs and longer development times. However, inhaled delivery of SOC compounds is currently being explored in ongoing clinical trials,^93^^,^^94^ while several other agents targeting various factors are also delivered by inhalation in ongoing clinical trials.^95^ The viability and advantages of inhaled drug delivery were recently exemplified in the TETON IPF trials with Treprostinil,^96^^,^^97^ a synthetic prostacyclin delivered by inhalation, which met its primary endpoint, as announced in 2025. Therefore, **EL244** emerges as a promising clinical candidate for the inhaled treatment of IPF and ILDs.

### Limitations of the study

The ATX/LPA axis is well established to promote the activation of several kinases that are targeted by the current IPF/ILD SOC treatment, while several kinases are thought to regulate PPARγ transcription and activity. Therefore, inhaled **EL244** should be co-administered with oral SOC compounds, a standard practice in most IPF clinical trials, to examine additive or toxic effects. Moreover, dose-finding studies should be conducted to further elucidate the engagement thresholds for the two targets, explore potential synergy, and identify the minimum effective concentration. Most importantly, inhaled toxicity studies will be essential to establish a safe use for **EL244**.

## Resource availability

### Lead contact

Further information and requests for resources and reagents should be directed to and will be fulfilled by the lead contact, Vassilis Aidinis (v.aidinis@fleming.gr).

### Materials availability

Reasonable amounts of **EL244** for research purposes can be obtained by the corresponding authors.

### Data and code availability

• All data are available upon reasonable request to the lead contact. RNA sequencing data have been deposited to Gene Expression Omnibus (GEO) (#GSE297484). In accordance with the HDX-MS community guidelines, Table S4 and Data S1 present the HDX summary and data table, respectively. Original western blot images have been deposited at Mendeley Data and are publicly available (DOI: https://doi.org/10.17632/g2wwfpfmby.1).
• All code of the RNA sequencing analysis is available at the GitHub repository: https://github.com/alex-galaras/matralis_et.al.2025.git.
• Any additional information required to reanalyze the data reported in this work paper is available from the lead contact upon request.

## Acknowledgments

We would like to thank A. Katsouda and A. Papapetropoulos for the 3T3-L1 cell line, the adipocyte differentiation reagents, and the associated protocol and expertise; B. Crestani for the NHLF clone; and the Biobanque CRB-Tumorothèque de Nice (CHU de Nice) for providing human lung tissue, as well as for their technical expertise and support. This research was co-financed by the European Union and Greek national funds through the Operational Program Competitiveness, Entrepreneurship and Innovation and the European Regional Development Fund, via the 10.13039/501100003448General Secretariat for Research and Innovation (GSRI) grant (T1EDK-0049 to V.A.). It was further partly supported by several 10.13039/501100013209Hellenic Foundation for Research and Innovation (HFRI) grants (#3565 to V.A., #01144 to C.M., #7337 to A.N.M., #3780 to P.H., and #5691 to A.G.), by the 10.13039/501100000780European Commission (EC) (#101037509 to A.A.) and the Cyclone supercomputer of The Cyprus Institute (#pro24a01). A.P. was supported by an EPSRC Research Fellowship (EP/V0117151/1) and BBSRC grants BB/Y004981/1 and BB/X018326/1. The authors acknowledge the MRC Equipment Grant MR/X013030/1. A.P. acknowledges support by the European Union via the Horizon Europe ERA Chair “MASSTRUCT” Project, number id 101183630. The funders had no role in the study’s design, data collection, analysis, or interpretation, nor in the writing of the manuscript or the decision to publish the results.

## Author contributions

K.D.P. and A.A. performed all HVTS computations and MD analyses. E.M.L. and A.N.M. synthesized all compounds. E.M.L., C.M., S.G.D., E.K., I.T., and A.N.M. performed PK/PD analyses. E.-A.S., E.K., and C.M. tested ATX inhibition of compounds, while S.W. and D.M. tested PPARγ agonism. P.K., D.N., E.-A.S., S.S., and C.M. performed animal studies and all related readout assays. C. Moro, S.S., and H.O.-P. performed hPCLS analysis. J.P.R.P. and A.P. performed and analyzed HDX-MS. K.M.A. and A.U.W. supervised the clinical applicability and translatability of findings in the context of the current state of the art. A.G. and P.H. performed and analyzed RNA sequencing. The manuscript was written by A.N.M. with the assistance of K.D.P., J.P.R.P., P.K., and C.M. It was edited by V.A. and critically reviewed by all authors.

## Declaration of interests

A.N.M. and V.A. are the inventors of a series of Greek National patents regarding **EL244** and the corresponding PCT application (WO2024134227A1) that has entered the National phases. V.A. is the founder and major shareholder of DrugTrek PC, a spinoff company of Fleming and UniPharma S.A. I.T. is the CEO and managing director of Uni-Pharma S.A. C. Moro and H.O.-P. are PKDERM employees. K.M.A. has obtained research grants from Boehringer Ingelheim, F. Hoffmann-La Roche, Chiesi, and Menarini; consulting fees from Boehringer Ingelheim, Hoffmann-La Roche, and GlaxoSmithKline; and support for attending meetings from Chiesi. She is on the speakers’ bureau of AstraZeneca, Boehringer Ingelheim, Chiesi, Hoffmann-La Roche, GlaxoSmithKline, Menarini, Guidotti, and Pfizer. She has attended advisory board meetings and scientific consultancies for Boehringer Ingelheim, Hoffmann-La Roche, AbbVie, Avalyn, Vicore, and GlaxoSmithKline. AUW reports consultancy fees from Boehringer Ingelheim, Roche, Veracyte, Chiesi, CSL Behring, Avalyn, and Foresee and payment or honoraria for lectures, presentations, manuscript writing, or educational events from Boehringer Ingelheim.

## STAR★Methods

### Key resources table

**Table:** 

REAGENT or RESOURCE	SOURCE	IDENTIFIER
**Antibodies**		

Anti-Collagen1a1	Thermo Fisher Scientific	Cat# PA5-29569; RRID:AB_2547045
Anti-alpha smooth muscle Actin	Abcam	Cat# ab5694; RRID:AB_2223021
Anti-beta Actin	Abcam	Cat# ab8227; RRID:AB_2305186
Goat anti-rabbit Ig, human ads-HRP	Southern Biotech	Cat# 4010-05; RRID:AB_2632593
Goat anti-Rabbit IgG (H + L) cross-absorbed secondary antibody, Alexa Fluor™ 488	Thermo Fisher Scientific	Cat# A-11008; RRID:AB_143165

**Biological samples**		

Human PCLS	Biobanque CRB-Tumorothèque de Nice, Biobanque CHU de Nice	N/A
Human lung fibroblasts	Department of Pulmonology, Bichat-Claude Bernard Hospital, Paris/France	N/A

**Chemicals, peptides, and recombinant proteins**		

*N*-Boc-ethanolamine	Merck	Cat#382027
4-hydroxybenzaldehyde	Merck	Cat#144088
triphenyl phosphine	Merck	Cat#T84409
*di*-isopropyl azodicarboxylate	Merck	Cat#225541
2,4-thiazolidinedione	Merck	Cat#375004
4-fluorobenzoylacetonitrile	Merck	Cat#681822
thiourea	Merck	Cat#T8656
iodine	Merck	Cat#207772
Copper chloride	Merck	Cat#8.18247
*tert*-butoxy nitrite	Merck	Cat#235385
1,2-dibromoethane	Merck	Cat#D40752
CDI	Merck	Cat#115533
1-boc-piperazine	Merck	Cat#343536
Cobalt chloride hexahydrate	Merck	Cat#255599
dimethylglyoxime	Merck	Cat#03858
Sodium borohydride	Merck	Cat#452882
TOOS	Merck	Cat#E8631
4-aminoantipyrene	Acros	Cat#103150250
Choline chloride	Merck	Cat#26978
Dimethyl sulfoxide	Merck	Cat#589569
Calcium dichloride	Merck	Cat#C5670
Hydrochloric acid 37.2%	Merck	Cat#258148
Sodium phosphate dibasic heptahydrate	Merck	Cat#S9390
Sodium phosphate monobasic monohydrate	Merck	Cat#S9638
Chloroform	Merck	Cat#102447
Methanol	Merck	Cat#1026002
Amplex red reagent	Thermo Fisher Scientific	Cat#A12222
16:0 lysophosphatidylcholine	Merck	Cat#855675C
Tris-base	Merck	Cat#10708976001
GLPG1690	MedChemExpress	Cat#HY-101772
Human autotaxin	Sino Biological	Cat#11308-H07H
Horseradish peroxidase	Merck	Cat#P8125
Choline oxidase from *Alcaligenes* sp.	Merck	Cat#C5896
Streptozotocin	Enzo Life Sciences	Cat#ALX-380-010
Glucose	Merck	Cat#G7021
Recombinant PPARγ LBD protein	Hinnah et al.^98^	N/A
Lipofectamine™ LTX Reagent	Invitrogen	Cat#15338500
Human recombinant TGF-β1	Peprotech	Cat#100-21
Human recombinant TNF-α	Peprotech	Cat#300-01A
Human plateled-derived growth factor-AB	Peprotech	Cat#100-AB
18:1 Lysophosphatidic acid (sodium salt)	Merck	Cat#857130P
Gentamicyne	Biowest	Cat#L0012-100
Antibiotic-antimycotic 100X	Biowest	Cat#L-0010-100
DMEM/F12	VWR	Cat#392-0411
Fetal bovine serum	VWR	Cat#S1810-500
ITS (Insulin Transferrin, Selenium) 100X	Merck	Cat#13146
Low melting agarose	Promega	Cat#V2111
Hank’s balanced salt solution	Merck	Cat#55037C
Thiazolyl Blue (MTT)	MedChemExpress	Cat#HY-15924
Drug chemical library	Prestwick	Cat#PCLCP50
Oil red O	Merck	Cat# O0625
10× Phosphate-buffered saline (PBS)	Gibco	Cat# 70011-044
1x DMEM high glucose	Gibco	Cat#41966-029
Amphotericin B	Gibco	Cat#15290-018
TRIzol™ Reagent	Thermo Fischer	Cat#15596026
Bleomycin hydrogen chloride (BLM)	Nippon Kayaku Co.	N/A
Nintedanib	Merck	Cat#SML2848
Propan-2-ol	Fisher Chemical	Cat#P/7500/17
M-MLV reverse transcriptase	Invitrogen	Cat#28025-013
SYBR green universal master mix	Invitrogen	Cat#4309155
dNTP set	Thermo Fisher Scientific	Cat#R0181
WESTAR-ONE PLUS	Cyanagen	Cat# XLSU178
2-Mercaptoethanol	Merck	Cat# M3148
Acrylamide	Merck	Cat# A8887
N,N′-Methylenebisacrylamide	Merck	Cat# M7279
Bromophenol Blue sodium salt	Merck	Cat# B8026
Glycerol	Merck	Cat#G5516
N,N,N′,N′-Tetramethylethylenediamine	Merck	Cat# T9281
Ammonium persulfate	Merck	Cat# A3678
2-Mercaptoethanol	Merck	Cat# M3148
Ethanol	VWR	Cat#20821.365
Paraformaldehyde (PFA)	Merck	Cat#P6148
Fast Green FCF	Glentham Life Sciences	Cat#GT-3407
Picric acid	Merck	Cat#197378
Direct Red 80	Merck	Cat#365548
Formaldehyde	PanReac Applichem	Cat#A0877, 0250
Acetic acid	Merck	Cat#33209
Thiazolyl blue tetrazolium bromide	Thermo Fisher Scientific	Cat#158990010
Optimem	Gibco	Cat#11058021
Eosin G	Roth	Cat#CI45380
Sodium Chloride	Fisher Scientific	Cat# 11984051
Deuterium oxide	Merck	Cat#613444
Glycine hydrochloride	Fisher Scientific	Cat# 10156800
Urea	Fisher Scientific	Cat# 15845488
TCEP	Merck	Cat# C4706
Guanidine Hydrochloride	Merck	Cat# G4505
0.1% formic acid in water	Fisher Scientific	Cat# 11947199
0.1% formic acid in acetonitrile	Fisher Scientific	Cat# 10118464

**Critical commercial assays**		

Dual-Glo® Luciferase Assay System	Promega	Cat#E2940

**Deposited data**		

RNA sequencing data	N/A	#GSE297484

**Experimental models: Cell lines**		

Human: HEK293T cells	DSMZ	ACC 635
3T3-L1 cell line	ATCC	N/A

**Experimental models: Organisms/strains**		

C57BL/6 mice	BSRC Al. Fleming	N/A

**Oligonucleotides**		

**Human**		
Primer: h*Acta2* Forward: TGAAGAGCATCCCACCCT	Εurofins	N/A
Primer: h*Acta2* Reverse: ACGAAGGAATAGCCACGC	Εurofins	N/A
Primer: h*Cd36* Forward: CGGCTGCAGGTCAACCTATT	Εurofins	N/A
Primer: h*Cd36* Reverse: CACCAATGGTCCCAGTCTCA	Εurofins	N/A
Primer: h*Col1a1* Forward: CGAAGACATCCCACCAATCAC	Εurofins	N/A
Primer: h*Col1a1* Reverse: CATCGCACAACACCTTGCC	Εurofins	N/A
Primer: h*Fabp4* Forward: AAACTGGTGGTGGAATGCGT	Εurofins	N/A
Primer: h*Fabp4* Reverse: GCGAACTTCAGTCCAGGTCA	Εurofins	N/A
Primer: h*Pparγ* Forward: AGAGCCTTCCAACTCCCTCA	Εurofins	N/A
Primer: h*Pparγ* Reverse: TCTCCGGAAGAAACCCTTGC	Εurofins	N/A
Primer: h*Rplp0* Forward: AATCTCCAGGGGCACCATTG	Εurofins	N/A
Primer: h*Rplp0* Reverse: CAGGGTTGTAGATGCTGCCA	Εurofins	N/A
Primer: h*Hprt1* Forward: CCTGGCGTCGTGATTAGTGAT	Εurofins	N/A
Primer: h*Hprt1* Reverse: AGACGTTCAGTCCTGTCCATAA	Εurofins	N/A
**Mouse**	–	–
Primer: m*Arg1* Forward: GTAGACCCTGGGGAACACTAT	Εurofins	N/A
Primer: m*Arg1* Reverse: ATCACCTTGCCAATCCCCAG	Εurofins	N/A
Primer: m*Cd36* Forward: ATTAATGGCACAGACGCAGC	Εurofins	N/A
Primer: m*Cd36* Reverse: TTCAGATCCGAACACAGCGT	Εurofins	N/A
Primer: m*Col1a1* Forward: CTACTACCGGGCCGATGATG	Εurofins	N/A
Primer: m*Col1a1* Reverse: CGATCCAGTACTCTCCGCTC	Εurofins	N/A
Primer: m*Fn1* Forward: GGCCACCATTACTGGTCTGG	Εurofins	N/A
Primer: m*Fn1* Reverse: GGAAGGGTAACCAGTTGGGG	Εurofins	N/A
Primer: m*Glut1* Forward: TCAACACGGCCTGCACTG	Εurofins	N/A
Primer: m*Glut1* Reverse: CACGATGCTCAGATAGGACATC	Εurofins	N/A
Primer: m*Fabp4* Forward: TGAAATCACCGCAGACGACAGG	Εurofins	N/A
Primer: m*Fabp4* Reverse: GCTTGTCACCATCTCGTTTTCTC	Εurofins	N/A
Primer: m*Pparγ* Forward: GCTCGCAGATCAGCAGACTCT	Εurofins	N/A
Primer: m*Pparγ* Reverse: GAGAAGCTGTTGGCGGAGAT	Εurofins	N/A
Primer: m*Tnc* Forward: TTCACAGTTTCCTGGACGGC	Εurofins	N/A
Primer: m*Tnc* Reverse: ACTTCCGGTTCAGCTTCTGTAAT	Εurofins	N/A
Primer: m*Timp1* Forward: CTTCTGCAACTCGGACCTGG	Εurofins	N/A
Primer: m*Timp1* Reverse: CATGACTGGGGTGTAGGCGT	Εurofins	N/A
OligodT	New England Biolabs	Cat#S1316S

**Recombinant DNA**		

Plasmid: pFA-CMV-hPPARα-LBD	Rau et al.^99^	N/A
Plasmid: pFA-CMV-hPPARγ-LBD	Rau et al.^99^	N/A
Plasmid: pFA-CMV-hPPARδ-LBD	Rau et al.^99^	N/A
Plasmid: pFR-Luc	Stratagene	Cat#219050
Plasmid: pRL-SV40	Promega	Cat#E2231

**Software and algorithms**		

GraphPad Prism version 7.00	GraphPad Software	GraphPad
Waters data analysis software	Waters	–
rDock molecular docking software	Vernalis and the University of York	rDock
NanoAnalyze version 3.7.5	TA Instruments	TA instruments
OpenMM 7.5	Stanford University, Memorial Sloan Kettering Cancer Center, and Rutgers University	OpenMM 7.5
Gaussian 09 (version D.01)	Gaussian, Inc.	Gaussian
DBSCAN	University of Munich	DBSCAN
PoseEdit	Universität Hamburg, ZBH-Center for Bioinformatics	PoseEdit
ChimeraX	University of California San Francisco	ChimeraX
Gypsum-DL	University of Pittsburgh, Pittsburgh, PA, 15260, USA	Gypsum-DL
xTB (v6.6.0)	University of Bonn	xTB
Asclepios KNIME	NovaMechanics	–
Enalos Asclepios KNIME	NovaMechanics	Enalos Asclepios
AmberTools21	Rutgers University, Michigan State University, University of California, Irvine University of Utah, Stony Brook University	AmberTools21
ProteinLynxGlobal Server (PLGS, v.3.0.3)	Waters Corporation	ProteinLynxGlobal Server
DynamX v. 3.0.0	Waters Corporation	DynamX
Deuteros v. 2.0	Lau, A.M. et al.,. Bioinformatics, 2021. **37**(2): p. 270–272.	Deuteros v. 2.0

**Other**		

Gel Imaging System	Biorad	Chemidoc XRS+
Saccharometer	Ascensia	Contour Care
Glucose strips	Ascensia	Contour Care
Standard Diet	Mucedola	Cat#4RF21
High Fat Diet	Research Diets	Cat#D12492
Control Diet	Research Diets	Cat#D12450Ji
Fluorimeter	Tecan	Infinite M200
Spectrophotometer	Molecular Devices	Optimax
Black 96-well plate, flat bottom with lid	Corning	Cat#3916
Glass screw cap vials (N8)	Isolab	Cat#095.00.001
Caps & septa for N8 vials without slit, PTFE	Isolab	Cat#096.00.001
Amersham™ Protran® nitrocellulose	Merck	Cat# GE10600002
Parafilm	Merck	Cat#P7543
pH meter	Adwa	AD1020
Analytical balance	Radwag	AS 62.R2
Non-CO_2_ incubator	HYBAID	Shake ‘n’ Stack
6-well plate	Thermo Fisher Scientific	Cat#140685
12-well plate	Thermo Fisher Scientific	Cat#150628
24-well plate	Thermo Fisher Scientific	Cat#142485
96-well plate	Thermo Fisher Scientific	Cat#167008
50mL tubes	Sarstedt	Cat#62547254
1.5mL centrifuge tubes	ABDOS	Cat#P10202
7mL polystyrene bijou containers	Thermo Fisher Scientific	Cat#129B
Paraffin Histoplast IM	Erpedia	Cat#8331
Embedding cassettes	Isolab	Cat#S.074.03.001.500
Sponges for embedding cassettes	Roth	Cat#TT56.1
Microscope slides	VWR	Cat#631-0108
Microscope cover glasses	VWR	Cat#631-1574
DPX mountant	Merck	Cat#06522
Molecular biology grade water	Cytiva	Cat#SH30538.02
Homogenizer T 25 digital ULTRA-TURRAX	IKA	Cat#0003725000
CFX96 real-time system	Bio-Rad	C1000 Touch
NanoDrop spectrophotometer	Thermo Fisher Scientific	Nanodrop 1000
Fresco 17 Microcentrifuge	Thermo Fisher Scientific	75002402
OPTImax microplate photometer	Molecular Devises	N/A
Vibratome	Leica	VT1200S
slide scanner	Olympus	VS200
Gemini 5U C18 column	Phenomenex	Cat# 00F-4435-E0
Waters-QQQ mass spectrometer	Waters	Waters-QQQ
Gemini guard column (4 × 3 mm)	Phenomenex	Cat#AJ0-7597
LPA-d9 internal standard mixture	Cayman	Cat#33479
Waters cIMS QTOF	Waters	Waters SELECT SERIES cIMS QTOF
HDX robot	Trajan scientific	Dual head parallel Trajan LEAP HDX robot
Bluestar prestained protein marker	Nippon Genetics	Cat#MWP03

### Experimental model and study participants details

#### Cell culture

**HEK293T cells** (German Collection of Microorganisms and Cell Culture GmbH, DSMZ) were cultured in Dulbecco’s modified Eagle’s medium (DMEM), high glucose supplemented with 10% fetal calf serum (FCS), sodium pyruvate (1 mM), penicillin (100 U/mL), and streptomycin (100 μg/mL) at 37°C and 5% CO2. The **3T3-L1** preadipocyte cell line (a kind gift from A. Katsouda and A. Papapetropoulos) was maintained in DMEM supplemented with 10% FBS, 1% penicillin/streptomycin, and 0.1% amphotericin B. Cells were cultured at 37^o^C, 5% CO2 and ≥95% relative humidity. Commercially available cell lines had been originally tested by the provider. Cell line doubling times and microscopy were used to verify the continuous absence of mycoplasma.

**Murine precision cut lung slices (PCLS)**.^51^^,^^55^ Briefly, wild type C57BL/6 mice received BLM (1U/kg) via oropharyngeal aspiration. Fourteen days after administration, mice were euthanized and lungs were perfused with PBS. Lungs were subsequently inflated through the trachea with pre-warmed (40-42^o^C) low melting-point agarose. Following agarose solidification, the left lateral lobe was excised and sectioned into 300μm-thick slices using a vibratome machine. Lung slices were placed in 12- or 24- well culture plates containing complete DMEM supplemented with 1% penicillin/streptomycin, and 0.1% amphotericin B, and maintained at 37^o^C, 5% CO2 and ≥95% relative humidity.

#### Animals

Wild type C57BL/6 mice were housed and bred under specific pathogen-free (SPF) conditions at 20°C–22°C with 55% ± 5% humidity, a 12-h light/dark cycle, and unrestricted access to food and water. Experimental cohorts were randomly assigned and consisted of sex- and age-matched littermates. In all experiments mice of both sexes and of 6–8 weeks of age were used, apart from the diabetes experiment where one-year old male mice were used, because female mice are thought to be less sensitive to the b-islet toxin streptozotocin. Daily health checks ensured proper animal welfare, and no unanticipated mortality occurred. At designated time points, euthanasia was carried out in a CO_2_ chamber using a gradual fill method to ensure humane treatment. All ARRIVE-compliant procedures received approval from the Protocol Evaluation Committee (PEC) of the Biomedical Sciences Research Center “Alexander Fleming” and were licensed by the Veterinary Authority of the Attica region, Greece (#927781, 2023 #983536, 2025). The institution’s Animal Welfare Body (AWB) oversaw compliance with animal welfare regulations.

#### Human samples

**Normal human lung fibroblasts (NHLFs)**, clone 832, were isolated from the adjacent healthy tissue of a patient undergoing open lung surgery for cancer at the Department of Pulmonology, Bichat-Claude Bernard Hospital, Paris/France; studies were approved by the Committee for Personal Protection (CPP)—Ile de France 1 (#0911932). The patient provided written consent to the use of his/her sample for research purposes; no compensation was provided. NHLFs were cultured in DMEM supplemented with 10% FBS, 1% penicillin/streptomycin, and 0.1% amphotericin B at 37^o^C, 5% CO2 and ≥95% relative humidity.

#### Human precision-cut lung slices (hPCLS)

hPCLS were prepared from tumour-free lung tissue obtained during surgical resection from a 71-year-old male patient undergoing surgery for pulmonary adenocarcinoma (Subject ID: LB26-0020). The biopsy was provided by the Biobanque CRB-Tumorothèque de Nice, Biobanque CHU de Nice (BB-0033-00025; approved protocol # CSE 2022-EV34), collected after written informed consent, in accordance with the Declaration of Helsinki and applicable French regulations for human biological materials.

The lung biopsy was rinsed thoroughly with Hank’s balanced salt solution (HBSS, Sigma-Aldrich) supplemented with antibiotics. The tissue was then infused with a 2.5% low-melting agarose solution prepared in DMEM/F-10 containing antibiotics, maintained at 40°C, and rapidly cooled to solidify the agarose. 8 mm tissue punches were then excised and sectioned into 300 μm-thick slices using the Compresstome VF-300-0Z (Precisionary Instruments). Each hPCLS was transferred into an individual well of a 24-well plate containing 500 μL of culture medium supplemented with 1× antibiotic–antimycotic solution (penicillin, streptomycin, amphotericin B), 50 μg/mL gentamicin, 1× insulin-transferrin-selenium (ITS) supplement, and 0.1% FBS. Slices were incubated at 37°C in a humidified atmosphere with 5% CO_2_. The culture medium was replaced after several hours.

### Method details

#### Virtual screening (VS)

Virtual screening (VS) of the Prestwick Chemical Library was performed by means of the rDock molecular docking software.^33^ Initially, the crystal structure of the ATX protein (PDB ID: 2XRG)^61^ was retrieved from the Protein DataBank and pre-processed using PDBFixer^100^ and pdb4amber from AmberTools21^101^ via the “Protein Structure Preparation” branch of the Enalos Asclepios KNIME pipeline (Figure S1).^102^ Preparation steps included addition of missing heavy and hydrogen atoms, reconstruction of non-terminal loops, conversion of non-standard residues to their standard counterparts, resolution of alternate atomic positions, and removal of heteroatoms. Simultaneously, the Prestwick compounds were prepared using their SMILES strings via the “Ligand Structure Preparation” workflow branch in the Asclepios KNIME pipeline. Hydrogens were added using OpenBabel^103^ through the AsclepiosAddHydrogens node, adjusting the protonation state to pH 7.4. The AsclepiosGenerate3DCoords node then converted the 2D structures to 3D conformers through energy minimization. VS calculations were carried out using the RunRxDock node with the RxDock engine.^33^ The binding site was defined based on the co-crystallized inhibitor using the reference ligand method, with a cavity radius of 6.0 Å and a small sphere radius of 1.5 Å. Cavity mapping employed a 0.5 Å resolution grid and the RbtCavityGridSF scoring function. Parameters were set to accept a maximum of one cavity with a minimum volume of 100 Å^3^. A cavity restrain function (weight = 1) was applied to prevent ligand escape during docking. Each compound was subjected to 50 independent docking runs. The resulting poses were ranked based on their intermolecular score, which reflects protein-ligand binding energy. Although total docking scores typically include intramolecular energy contributions, this term was excluded due to its known reduction in predictive reliability.^104^^,^^105^ Conformations were further evaluated based on root-mean-square deviation (RMSD) relative to the reference pose, prioritizing top-scoring ligands for *in vitro* testing on the basis of both docking score and key interactions with the Zn^2+^ ions, catalytic residue Thr209, and the hydrophobic pocket.

#### Molecular docking

Molecular docking at ATX and PPARγ was performed using NovaMechanics Asclepios.^102^^,^^106^ 3D structures and physiologically relevant protonation states (pH 7.4) were generated via Gypsum-DL,^107^ which converts SMILES or SDF files into 3D models with alternate ionization, tautomeric, chiral, and conformational states. Dimorphite-DL^108^ was used for empirical protonation state prediction, based on substructure searches and a curated ionizable compound database. To refine results, pKa values of key functional groups were predicted with MolGpka,^109^ which uses a graph-convolutional neural network trained on chemical patterns. Only the most probable protonation variant per compound, aligned with physiological pΗ, was retained. Initial geometry optimization used UFF via Gypsum-DL,^110^ followed by refinement with xTB (v6.6.0)^111^ using the GFN2-xTB method^112^ and ALPB solvation.^113^ Docking cavities were defined via the reference ligand method; 100 RxDock runs per ligand were performed. The PPARγ ligand-binding domain (PDB ID: 5YCP) complexed with RGZ was used as the structural model.^114^ Only crystallographically known stereoisomers of RGZ, TGZ, and PGZ were considered, in line with prior studies.^115^ Docking was executed using RxDock, a fork of rDock,^33^ and poses were ranked by intermolecular scores reflecting protein-ligand binding free energy.^104^

#### Molecular dynamics simulations

The top-scoring docking poses for each ligand were used as initial conformations in Molecular Dynamics (MD) simulations with OpenMM 7.5,^100^ implemented via the Asclepios KNIME workflow. Protonation states of ATX and PPARγ residues were assigned using the AsclepiosPDBFixer node, which integrates PDBFixer and pdb4amber^116^ to add missing residues/heavy atoms, remove heteroatoms, and standardize residue names. Disulfide bonds were incorporated using pdb4amber and prepareforleap,^116^ as applicable. The AMBER14SB force field^117^ was used for both proteins. Given its functional relevance, only the N-glycan linked to ATX Asn524^118^ was retained, modeled using the glycan from PDB 5MHP^119^ and parameterized with GLYCAM06 24.^120^ Ligand geometry optimization was performed at the B3LYP/6-31G∗ level,^121^^,^^122^^,^^123^ followed by HF/6-31G∗ electrostatic potential (ESP) calculations using Gaussian 09 (version D.01), via PyRED on the R.E.D. Server.^124^^,^^125^^,^^126^^,^^127^ RESP charges were derived using the standard two-stage fitting,^126^ and GAFF2.1^128^^,^^129^^,^^130^ parameters were assigned. TIP3P water molecules^131^ were added with a 10 Å buffer, using rectangular periodic boundary conditions. Neutralizing Na^+^/Cl^−^ ions were introduced. Energy minimization (20,000 steps) applied positional restraints on protein-ligand atoms, gradually reduced every 5,000 steps (100 → 0 kcal mol^−1^Å^−2^). Systems were then equilibrated (1 fs time step): heating (200 ps, NVT) from 0→300 K in 3 ps intervals using a Langevin thermostat,^132^ with 20 kcal mol^−1^Å^−2^ restraints on non-hydrogen atoms. For ATX, additional Zn^2+^-centered restraints (100 kcal mol^−1^Å^−2^) were applied on coordinating atoms^118^ during both equilibration and production. Pressure was gradually ramped from 0.1 to 1 atm (20 ps intervals, NPT) using a Monte Carlo barostat,^133^^,^^134^ with 2 kcal mol^−1^Å^−2^ restraints, later lifted over 1 ns, followed by 1 ns unrestrained NPT. Long-range electrostatics were handled via Particle Mesh Ewald,^135^ with 10 Å cutoffs for electrostatics and van der Waals interactions. Production MD (200 ns) used 2-fs time steps, constraining bonds involving H atoms. A total of 10,000 frames were saved and analyzed using CPPTRAJ from AmberTools21.^101^ RMSDs (mass-weighted) were computed for Cα atoms (proteins) and heavy atoms (ligands). Hydrogen bonds were evaluated over the final 5,000 frames, with a 3.5 Å donor–acceptor distance and 150° angle cutoff. Clustering was performed using DBSCAN (epsilon = 3 Å, minPts = 25, sieve = 10), and centroid structures were visualized using PoseEdit^136^ and ChimeraX.^137^^,^^138^^,^^139^ MM-GBSA binding free energies were calculated using the AmberTools21 MMPBSA.py implementation in the Enalos Asclepios workflow,^101^ based on gas-phase interaction and solvation energies (GB model).^140^ Enthalpic contributions were averaged over the final 5,000 frames, with per-residue decomposition. Standard error of the mean (SEM) was used to report statistical uncertainties: σ/√N (*N* = 5000).

#### Amplex red assay

ATX inhibitory activity *in vitro* of examined compounds was assessed with the Amplex Red assay,^32^^,^^35^^,^^36^ as recently published in detail.^37^ The assay was done in Amplex Red Assay buffer (Tris 50 mM, CaCl2 5 mM, pH 8). Briefly, 50 μL of increasing concentrations of each compound were placed in separate wells of a black 96-well plate. 50 μL of working solution of ATX (6.4 nM) were added to the wells and incubated for 15 min at 37°C to allow the compounds to interact with the enzyme. 50 μL of the LPC-containing assay buffer (200 μM) were added to the wells and incubated for 30 min at 37°C to allow the enzyme to initially interact with its substrate. 50 μL of the detection mix (Amplex Red 200 μM, choline oxidase 0.4 U/mL, horseradish peroxidase 4 U/mL) were added to the plate wells. Fluorescence was measured in a Tecan M200 Fluorimeter using excitation at 530 nm and reading at 590 nm, every 5 min for 30 min. The velocity of the reaction and the remaining ATX activity were calculated at the linear phase of the reaction. Dose-response inhibition graphs were plotted with GraphPad Software. For each compound, IC50 value was determined as the compound concentration that results in a 50% inhibition of the enzyme activity.

To determine the mode of inhibition of ATX, various concentrations of TGZ, compound **5** and **EL244** were tested against various LPC concentrations. Specifically, TGZ was tested against 6.25, 12.5, 25, 50 and 100 μM LPC, compound **5** was tested against 3.125, 6.25, 12.5, 25, 50 and 100 μM LPC, while **EL244** was tested against 25, 50, 100 and 150 μM LPC. Lineweaver–Burk graphs depicting the reciprocal of velocity (1/V) against the reciprocal of substrate concentration (1/S) were plotted with GraphPad Software.

#### TOOS assay

ATX inhibitory activity *ex vivo* of examined compounds was assessed via the colorimetric TOOS assay.^32^^,^^35^^,^^36^ The assay was done in Lyso-PLD buffer (100 mM Tris-Cl, pH 8, 500 mM NaCl, 5 mM MgCl2, 5 mM CaCl2). A mix of Lyso-PLD buffer and serum (10:1) was prepared. LPC 16:0 was added to this mix at a final concentration of 2 mM. Increasing concentrations of compounds and a choline standard curve (0–100 μM) were prepared using the Lyso-PLD buffer/serum/LPC mix and 100 μL of these were placed in separate wells of a 96-well plate, followed by a 5 h incubation at 37°C. 100 μL of color mix (TOOS 0.3 mM, 4-AAP 0.5 mM, choline oxidase 2 U/mL, HRP 7.95 U/mL in Lyso-PLD buffer) were added to each well. Color was measured at 550 nm using an Optimax spectrophotometer. Background was subtracted from all values to account for the endogenous LPC and choline in the serum. Produced choline levels were calculated from the choline standard curve and indicate the amount of substrate (LPC) that had been consumed in each well corresponding to Lyso-PLD activity.

#### PPARα/γ/δ hybrid reporter gene assays

All well-established assays were performed following straightforward methodology.^49^ Plasmids pFA-CMV-hPPARα-LBD, pFA-CMV-hPPARγ-LBD and pFA-CMV-hPPARδ-LBD code for the hinge region and ligand binding domain of the canonical isoform of the respective nuclear receptor and were used to express the Gal4 hybrid receptors. pFR-Luc (Stratagene, La Jolla, CA, USA) and pRL-SV40 (Promega, Madison, WI, USA) were used as Gal4-responsive reporter and as internal control in the hybrid reporter gene assays, respectively. HEK293T cells were seeded in 96-well plates (3×10^4^ cells/well). Before transfection, the medium was changed to Opti-MEM without supplements, and transient transfection with the plasmids pFR-Luc, pRL-SV40, and one pFA-CMV-NR-LBD clone was carried out using Lipofectamine LTX reagent (Invitrogen, Carlsbad, CA, USA) according to the manufacturer’s protocol. Five hours later, the medium was changed to Opti-MEM supplemented with penicillin (100 U/mL) and streptomycin (100 μg/mL), and additionally containing 0.1% dimethyl sulfoxide (DMSO) and compound **5**, **EL244**, or 0.1% DMSO alone as an untreated control. Following a 16 h incubation, cells were assayed for luciferase activity using the Dual-Glo Luciferase Assay System (Promega) according to the manufacturer’s protocol and in a Tecan Spark luminometer (Tecan Deutschland GmbH, Germany). Normalisation of transfection efficiency and cell growth was performed by dividing firefly luminescence by Renilla luminescence and multiplying the result by 1000, yielding relative light units (RLU). Fold activation was obtained by dividing the mean RLU of the test compound by the mean RLU of the untreated control. Max relative activation refers to fold reporter activity divided by the fold activation of the respective reference agonist (PPARα: 1 μM GW7647; PPARγ: 1 μM pioglitazone; PPARδ: 1 μM L165,041) treated cells. All hybrid assays were validated using dedicated reference agonists, yielding EC50 values consistent with the literature.

#### BLM-induced pulmonary fibrosis

BLM-induced pulmonary fibrosis was induced via the oropharyngeal aspiration of BLM (0.8 U/Kg).^54^^,^^55^^,^^141^ The dose and route of administration have been selected following extensive local testing to minimise lethality while preserving a solid and reproducible fibrotic profile. Readout assays include weight and overall health status, pulmonary oedema and inflammation, collagen levels determination (soluble in BALFs, Q-RT-PCR and histology in lung tissue), lung histology with specialised stainings (Fast green/picrosirius red), as well as the measurement of respiratory functions (flexiVent, SQIREQ); a detailed protocol is publicly available at protocols.io.

#### Drug inhalation

Drug inhalation of compound **5** and **EL244** to conscious, softly restrained mice was performed using the inExpose system (Scireq).^55^ Compound **5** and **EL244** were diluted in Kolliphor (15% and 10%, respectively) to final concentrations of 6.5 and 9.02 mg/mL, respectively. Volumes of 1 mL (for **5**) and 0.6 mL (for **EL244**) were aerosolized over 10 and 15 min, respectively in groups of 6 mice, corresponding to a final estimated dose of 15mg/Kg per mouse. Control groups received an aerosolized solution of Kolliphor (10–15%) in saline.

#### Induction of type-2 diabetes

One-year old male mice were given a High-Fat Diet (D12492, Research Diets) for 10 weeks. During the sixth week of the HFD period, mice received three streptozotocin injections (40mg/kg). Control mice that were consuming a control diet (D12450Ji, Research Diets) received injections with the solvent of STZ, 50 mM sodium citrate buffer, pH 4.5. During the last two weeks of the HFD treatment, a subgroup of prediabetic mice was daily administered EL244 through intraperitoneal injections (50 mg/kg). Disease induction and EL244 efficacy were assessed using an Oral Glucose tolerance test (oGTT), as widely used.^64^^,^^65^^,^^66^ 6-hr-fasted mice were administered a 10% glucose solution at 1 g/kg orally. Blood glucose levels were measured with a saccharometer before glucose administration and at 15, 30, 60, 90, and 120 min thereafter. Insulin resistance was assessed by the Area under curve (AUC) of the oGTT.

#### Differentiation of 3T3-L1 cell line into adipocytes

Adipogenic differentiation was carried out following a well-established protocol.^63^^,^^142^ Upon reaching approximately 70% confluence, cells were induced to differentiate (day 0) by replacing the culture medium with complete DMEM containing 10μg/mL insulin. Experimental groups received either **EL244** (1μM or 10μΜ) or the standard adipogenic induction cocktail consisting of 1μM dexamethasone (DEX) and 0.5mM 3-isobutyl-1-methylxanthine (IBMX), as indicated. On day 3, the medium was replaced with complete DMEM supplemented with 10μg/mL insulin, with continued exposure to **EL244** (1μM or 10μM) in the respective treatment groups. On day 6, the medium was refreshed with complete DMEM containing the corresponding concentrations of **EL244**. By day 8, cells exhibited morphological characteristics consistent with fully differentiated adipocyte-like cells.

#### Oil red O staining

Differentiated adipocytes were fixed with 10% formalin solution for 2 h, washed with 60% isopropanol, and stained with filtered 0.3% (w/v) Oil red O solution for 20 min. Following staining, cells were washed three times with dH2O to remove excess dye and observed using a bright-field microscope. Fow quantification, cells were destained with 100% isopropanol and lipid accumulation levels were quantified by Oil red O absorption at 500nm.

#### Treatment of mPCLS with inhibitors

After an overnight equilibration period, slices were treated with either GLPG-1690 (30μM) or compound **5** (30μM) for 72 h. Culture medium was refreshed every 24 h throughout the treatment period. For downstream gene expression analysis, three slices were pooled per sample for RNA extraction. Control PCLS were generated from saline-treated mice. Immunohistochemistry in mouse PCLS was performed on deparaffinized sections following antigen retrieval (citrate buffer, pH 6.0), blocking (10% goat serum/2% BSA), and overnight incubation with primary antibody against COL1A1 (1:100) at 4^o^C. Fluorophore-conjugated secondary antibody (1:500) was applied for 1 h at room temperature, and nuclei were counterstained with DAPI. Brightfield and fluorescence imaging were performed using the Olympus VS200 slide scanner.

#### Treatment of NHLFs with EL244

For experiments, NHLFs were seeded in 6-well plates at a density of 2 × 10^5^ cells per well. After overnight serum starvation, cells were pre-treated with **EL244** or vehicle (concentrations specified in the corresponding figures) for 1 h, followed by stimulation with TGFβ (10 ng/mL) for 24 h.

#### Mitotracker assay

NHLFs were plated at a density of 1,2 × 10^4^ cells/well of a 96-well plate. The next day TGFβ (10 ng/mL) and **EL244** (25 μM) or vehicle (DMSO) were added to the respective wells and incubated for 24 h. Medium was replaced with DMEM without phenol red that contained LumiTracker Mito Red CMXRos at 25 nM and a 30 min incubation followed. Medium was replaced with fresh DMEM without phenol red and without Lumitracker. Fluorescence was measured (excitation at 580 nm and reading at 600 nm) with a Tecan M200 Fluorimeter and normalized with cell number per well as measured with a Tecan Cyto Spark after cell fixation with PFA at a final concentration of 2%.

#### Viability assessment of hPCLS

Cell viability was assessed the day after the hPCLS isolation using the MTT assay. hPCLS were incubated for 3 h in MTT solution (1 mg/mL) at 37°C, 5% CO_2_. Formazan crystals were then solubilized with 500 μL of isopropanol for 2 h at room temperature or overnight at 4°C, and absorbance was measured at 560 nm using a GloMax Explorer Multimode Microplate Reader.

#### Fibrosis induction and treatments in hPCLS

Fibrosis was induced by a pro-fibrotic cocktail (PFC; 5 ng/mL TGF-β1, 5 μM PDGF-AB, 10 ng/mL TNF-a and 5 μM LPA 18:1), following a well-established protocol.^73^ Individual PCLS were first incubated with the PFC for 48 h. After this initial exposure, the medium was renewed and PCLS were treated for an additional 72 h with either PFC alone or PFC supplemented with **EL244** (25 μM or 50 μM). Untreated PCLS served as controls.

#### Protein extraction and western blotting

Cells were washed twice with ice-cold 1× PBS and lysed in RIPA buffer containing 50 mM Tris-HCl (pH 7.4), 1 mM EDTA, 150 mM NaCl, 0.25% (w/v) sodium deoxycholate, 1% (v/v) Triton X-100, 0.1% (w/v) SDS and 1 mM PMSF. Equal amounts of protein extracts (30 μg) were separated into 10% SDS-PAGE gels and subsequently electro-transferred onto nitrocellulose membranes. Membranes were blocked for 1 h at room temperature in 1X TBS-T containing 5% non-fat dry milk. Blots were incubated overnight at 4°C with primary antibodies against COL1A1 (1:2000), ACTA2 (1:2000), and β-ACTIN (1:2000). After washing, membranes were incubated for 1 h at room temperature with HRP-conjugated anti-rabbit secondary antibody (1:5000). Immunoreactive protein bands were detected using ECL.

#### Isothermal titration calorimetry (ITC)

ITC was performed^49^ on a TA Instruments Affinity ITC (TA Instruments, New Castle, Delaware, USA), using recombinant PPARγ LBD protein dissolved in a buffer containing 25 mM HEPES, 150 mM KF, 5 mM DTT, 10% w/v glycerol and 1% DMSO at pH 7.5. Compound **5** or **EL244** was dissolved to a final concentration of 300 μM in the same buffer, placed into the ITC syringe and titrated to 172 μL of PPARγ LBD protein (64 μM). The titration was performed at 25°C with a stirring rate of 75 rpm. An interval of 300 s was maintained between injections. ITC raw data were analyzed with the NanoAnalyze software package (version 3.7.5). An independent binding model was used to fit the reaction enthalpy (ΔH), binding affinity constant (KD), and stoichiometry (n). Free energy change (ΔG) was calculated with the equation ΔG = −RT ln K and the entropy (ΔS) was calculated with the equation ΔG = ΔH−TΔS.

#### HPLC-MS/MS

Plasma (50μL), BALF (300 μL) or homogenized lung tissue (10–50 mg) were mixed with ice-cold PBS spiked with the internal standard mixture (LPA-d9, Cayman) in a glass tube (total final volume 1mL). 2 mL of ice-cold chloroform and 1 mL of methanol was added in each sample, following by vortexing for 1 min and then centrifugation at 4°C for 5 min at 4000 g. The lower transparent organic phase (chloroform phase-contains the neutral lipids) was collected in a glass tube. The remaining aqueous phase (upper phase) was left on ice for 10 min and acidified (pH 3–4) with Formic Acid 10% in water. Then 1.5 mL chloroform was added followed by thorough mixing for 1 min and centrifugation at 4°C for 5 min at 2000 g. The lower organic phase was collected and neutralized to pH 6–7. The neutralized organic phase from the acid extraction were evaporated to dryness. After evaporation we redissolve in water/methanol (1:9) with 0.1% formic acid and run in the mass spec. All MS quantitation lipid standards were purchased from Cayman unless otherwise mentioned. All LPA species analyzed in this study were quantified using the multiple reaction monitoring (MRM) scanning method on an Waters-QQQ mass spectrometer. All data were acquired and analyzed using the Waters data analysis software. The LC separation was achieved using a Gemini 5U C18 column (Phenomenex, 5 μm, 50 × 4.6 mm) coupled to a Gemini guard column (Phenomenex, 4 × 3 mm). The LC solvents were as follows: buffer A, 85% CAN, 5% MeOH +0.1% formic acid +10% 10 mM ammonium formate buffer pH5; buffer B, 95:5 (v/v) ACN/MeOH +0.1% formic acid. A typical LC-run was 35 min, with the following solvent run sequence post injection: 0.35 mL/min 5% B for 5 min, linear gradient of B from 0 to 95% over 25 min, and re-equilibration with 0.5 mL/min of 5% B for 5 min. All lipid estimations were performed using an electrospray ion (ESI) source, with the following MS parameters: turbo spray ion source, medium collision gas, curtain gas = 20 L/min, ion spray voltage = −5000 V (negative mode), at 350°C. All the endogenous lipid species were quantified by measuring the area under the curve in comparison to the respective internal standard, and then normalized to the total protein content of the liver tissue. The following table presents the MRM used for LPA quantification.

**Table:** 

Species targeted	Precursor Ion Mass	Product Ion Mass	Collision Energy (V)	Ionization Mode
LPA 14	381	153	20	Negative
LPA 16	409	153	20	Negative
LPA 18	437	153	20	Negative
LPA 18:1	435	153	20	Negative
LPA 18:2	433	153	20	Negative
LPA 20:4	457	153	20	Negative
LPA 22:6	481	153	20	Negative

#### RNA sequencing

RNA quality was assessed using an Agilent TapeStation 4150 with the High Sensitivity DNA ScreenTape (Agilent), according to the manufacturer’s protocols. For library preparation, the QuantSeq 3’mRNA-Seq Library Prep Kit (Lexogen) was used according to manufacturer’s instructions. Briefly, 500 ng of RNA from each sample was used for first-strand synthesis, followed by RNA template removal, and second-strand synthesis initiated by random primers. In-line barcodes were introduced at this step, and this was followed by magnetic bead-based purification. The resulting libraries were amplified for 15 cycles and re-purified, with quantity and quality assessed on a Qubit 4 Fluorometer with the Invitrogen Qubit dsDNA HS Assay and an Agilent Tapestation 4150 with the High Sensitivity D1000 assay kit, respectively. The quantified libraries were pooled equimolarly; 50ng of total library mix was used for Adapter Conversion PCR Amplification using the Universal Library Conversion Kit (App-A), Version: V1.0 (MGI Tech Co., Ltd.), according to the manufacturer’s instructions.

The quantity and quality of the purified adapter conversion PCR (AC-PCR) product were evaluated on a Qubit 4 Fluorometer with the Invitrogen Qubit dsDNA HS Assay and on an Agilent Tapestation 4150 with the High Sensitivity D1000 assay kit, respectively. This was followed by DNA denaturation, Single-Strand Circularisation, Enzymatic Digestion, Enzymatic Digestion Product Cleanup and Quality Control steps on 1 pmol of AC-PCR product, according to the Universal Library Conversion Kit (App-A) User Manual (Version: A3).

Finally, 60 fmol of ssCirDNA were used for DNB preparation and sequencing on a DNBSEQ-G400 platform at the BSRC Alexander Fleming Genomics Facility, using a G400 App-A FCS SE100 High-throughput Sequencing Set (MGI Tech Co., Ltd.), according to the manufacturer’s instructions.

Alignment of the FASTQ files was performed against the mouse genome build mm10 using a custom Bash script.^143^ Read counting on the 3′ UTRs and differential expression analysis were conducted using the metaseqR2 bioconductor package.^144^ Genes corresponding to the following biotypes were excluded: polymorphic_pseudogene, processed_transcript, pseudogene, IG_V_pseudogene, misc_RNA, IG_C_gene, IG_J_gene, IG_D_gene, IG_LV_gene, TR_V_gene, TR_V_pseudogene, TEC, and processed_pseudogene. Gene counts were normalized with DESEQ^145^ through metaseqR2. Genes with more than 10 counts in at least 50% of the samples were kept for further processing. For differential expression analysis, the PANDORA algorithm^146^ was used to estimate a meta *p*-value integrating the results from the DESEQ, DESEQ2,^147^ edgeR,^148^ limma,^149^ NBPSeq,^150^ and NOISeq^151^ algorithms. All additional parameters were set to default values.

Pathway enrichment analysis was performed for the Gene Ontology Biological process (BP) and Molecular Function (MF) terms through GeneCodis 4.^152^ The volcano plots, scatterplots, Venn diagrams, and dot plots were generated using the ggplot2 Bioconductor package.

#### HDX-MS

All HDX experiments were performed using a dual head parallel Trajan LEAP automation system (Carrboro, NC, USA).^153^ Briefly, 5μL of reconstituted Autotaxin (1.25mg/mL) with and without EL244, was diluted into 100μL of labeling buffer (10mM PBS, 150mM sodium chloride, pD = 7.4 in D2O). pD was determined using pH measurements with a glass electrode and corrected for the isotopic effect.^154^ Samples were labeled in quadruplicate for each labeling time (30s, 300s and 3000s) at 25°C. For experiments with bounded Autotaxin, both the protein stock and labeling buffer contained 50μM of the ligand to ensure >95% bound protein during labeling.^155^ Afterward, 100 μL of labeled samples were quenched by mixing with equal amounts of precooled quench buffer (100mM glycine, 3M urea, 0.5M TCEP, pH 2.3 in water) at 1°C and held at that temperature for 120s to improve disulfide bond reduction. Non-deuterated controls and initial peptide map samples were prepared identically but using 10mM PBS, 150mM sodium chloride, pH = 7.4 in water as labeling buffer. Immediately after, 198μL of the sample was injected into a temperature-controlled chromatography cabinet connected to two ACQUITY I Class binary pumps. Sample was passed at 200 μL min–1 with 0.1% formic acid in water through a pepsin column (2.1 × 30 mm) immobilized in house kept at room temperature for 210s. Resulting peptic peptides were trapped and desalted in a XBridge C8 (2.1 × 5 mm, 5 μm) VanGuard precolumn (Waters Corporation) and separated using a Waters XBridge peptide BEH C18 analytical column (1.0 × 50 mm, 3.5 μm) with an 10 min linear gradient of 0.1% formic acid in acetonitrile increasing from 13 to 40% at 100 μL min–1. To avoid peptide carryover, the pepsin column was washed two times after each run with 85 μL of pepsin wash (2 M guanidine HCl, 5% acetonitrile, 100 mM phosphate buffer, pH 2.5) and blanks ran every 3 runs. Peptide masses were measured using a Waters SELECT SERIES cyclic IMS QTOF system using positive mode ionization and single pass ion mobility. All HDX experiments were measured using HDMS mode while initial peptide maps used HDMSE with a collision energy ramp of 20-45v.^156^

HDMSE files were processed using ProteinLynxGlobal Server (PLGS, v.3.0.3) (Waters Corporation) and then imported into DynamX v. 3.0.0 (Waters Corporation).^156^ Peptides were manually curated in DynamX and HDX data exported into Deuteros v. 2.0^157^ for statistical analysis and identification of significant peptides.^60^ Significant peptides were mapped into the crystal structure 2XR9.^61^ To allow access to the HDX data of this study, the HDX data summary table (Table S4) and the HDX data table (Table S4B) are included in the supporting information as per consensus guidelines.^158^

### Quantification and statistical analysis

Statistical significance was assessed with the GraphPad software and its built-in recommendations. For analysis of two groups, Student’s *t* test was performed. For analysis of multiple groups, one-way ANOVA was executed. Post hoc Tukey’s test was used in the case of normally distributed data. In the case of unequal SDs, Brown-Forsythe and Welch ANOVA and post-hoc Games-Howell’s multiple comparison test were used. In the case of non-normally distributed data, Kruskal-Wallis test was performed, followed by post hoc Dunn’s test. Data in box and whiskers include the median (line), interquartile range (box), and minimum and maximum range (tails). In panels where bar graphs are shown, means and SEM are depicted. ^∗/∗∗/∗∗∗/∗∗∗∗^ denote *p* < 0.05/0.01/0.001/0.0001, respectively. *n* represents number of plate wells in cell experiments, slices in PCLS experiments or number of animals in animal experiments. Details can be found in the Figure Legends.

#### Image creation

Third-party images were created at bioRender.com under the relevant agreements; CR29DFZVMG (Figure 7C) and ZP29DG08Y0 (Figure S4E).

#### Synthesis

##### Synthesis of compounds 1 and 4

***tert*-butyl (2-(4-formylphenoxy)ethyl)carbamate (i) (****https://doi.org/10.1021/jm0510880****)**. To a solution of 2-(boc-amino)ethanol (1 g, 6.20 mmol), 4-hydroxybenzaldehyde (0.91 g, 7.44 mmol) and triphenyl phosphine (2.44 g, 9.30 mmol) in dry THF (25 mL) was added *di*-isopropyl azodicarboxylate (1.83 mL, 9.30 mmol) dropwise at 0°C. The mixture was then allowed to stir at rt for 2.5 h. The solvent was removed under vacuum, the residue was dissolved in ethyl acetate (50 mL), washed with 1N NaOH (10 mL), water (10 mL) and brine (10 mL), dried (Na_2_SO_4_), filtered and concentrated in vacuum. The residue was purified by flash column chromatography eluted with hexane:ethyl acetate (4:1), furnishing the desired product as a white solid. Yield = 1.64 g (quant.). ^1^H-NMR (CDCl_3_, 400 MHz) *δ* 1.48 (s, 9H), 3.55 (d, *J* = 4.5 Hz, 2H), 4.11 (t, *J* = 5.0 Hz, 2H), 5.13 (brs, 1H), 7.05 (d, *J* = 8.5 Hz, 2H), 7.86 (d, *J* = 9.2 Hz, 2H), 9.91 (s, 1H). MS [ESI+] m/z 266.1 [M + H]^+^.

***tert*-butyl (*E*)-(2-(4-((2,4-dioxothiazolidin-5-ylidene)methyl)phenoxy)ethyl)carbamate (ii) (****https://doi.org/10.1021/jm0510880****)**. To a solution of compound **i** (1.50 g, 5.65 mmol) in dry toluene (15 mL), 2,4-thiazolidinone (0.80 g, 6.79 mmol) was added, followed by piperidine (0.28 mL, 2.83 mmol) and acetic acid (0.162 mL, 0.83 mmol). The mixture was stirred at reflux for 8 h and then it was allowed at rt overnight. The resulting solid precipitated was filtered, washed with toluene (3 mL) and hexane (5 mL), and dried at 45°C overnight to give the desired product as a beige/brownish amorphous solid. Yield = 1.63 g (80%). ^1^H-NMR (DMSO-d_6_, 400 MHz) *δ* 1.38 (s, 9H), 3.31 (m, 2H), 4.06 (m, 2H), 7.01 (s, 1H), 7.10 (d, *J* = 7.4 Hz, 2H), 7.55 (d, *J* = 8.7 Hz, 2H), 7.75 (s, 1H), 12.49 (brs, 1H). MS [ESI+] m/z 365.2 [M + H]^+^.

***tert*-butyl (2-(4-((2,4-dioxothiazolidin-5-yl)methyl)phenoxy)ethyl)carbamate (iii) (****https://doi.org/10.1021/jm0510880****)**. The thiazolidinone derivative **ii** (0.80 g, 2.20 mmol) and dry magnesium turnings (1.07 g, 43.91 mmol) were added in a flask and air was removed in vacuum. Then Argon was placed in the flask and anhydrous methanol (27 mL) was added. The mixture was stirred at rt and under Ar for 4h. The reaction mixture was acidified with 6N HCl to pH 5–6 and extracted with dichloromethane (2 × 25 mL) and the combined organic phase was washed with water (15 mL) and brine (15 mL), dried (Na_2_SO_4_), filtered and concentrated in vacuum. The residue was purified by flash column chromatography eluted with hexane:ethyl acetate (3:2), affording the desired compound as a yellow oil. Yield = 0.37 g (46%). ^1^H-NMR (CDCl_3_, 400 MHz) *δ* 1.50 (s, 9H), 3.13 (dd, *J*_*1*_ = 3.5 Hz, *J*_*2*_ = 14 Hz, 1H), 3.44 (dd, *J*_*1*_ = 3.5 Hz, *J*_*2*_ = 14.0 Hz*,* 1H), 3.58 (brd, *J* = 4 Hz, 2H), 4.06 (t, *J* = 5 Hz, 2H), 4.53 (dd, *J*_*1*_ *=* 3.5 Hz, *J*_*2*_ = 9.5 Hz*,* 1H), 5.03 (brs, 1H), 6.89 (d, *J =* 8.0 Hz, 2H), 7.21 (d, *J* = 8.0 Hz, 2H), 8.99 (brs, 1H). MS [ESI+] m/z 367.1 [M + H]^+^.

**Synthesis of compounds iv and v (****https://doi.org/10.1021/jm0510880****)**. A solution of 4N HCl in dioxane (1.90 mL, 7.61 mmol) was added at once to compounds **ii** and **iii** (0.22 g, 0.61 mmol) and the mixture was stirred at rt for 4h. The solvent was distilled in vacuum, the residue was washed with diethyl ether (15 mL) and dried, providing the desired products as a white solids.

**(*E*)-5-(4-(2-aminoethoxy)benzylidene)thiazolidine-2,4-dione hydrochloride (iv)**. Yield = 0.111 g (quant.). ^1^H-NMR (CH_3_OD, 400 MHz) *δ*. 3.45 (dd, J_1_ = 4.2 Hz, J_2_ = 14.1 Hz*,* 1H), 4.55 (brs, 2H), 6.98 (d, J *=* 8.0 Hz, 2H), 7.23 (d, J = 8.0 Hz, 2H), 7.91 (s, 1H). MS [ESI+] m/z 302.0 [M + H]^+^.

**5-(4-(2-aminoethoxy)benzyl)thiazolidine-2,4-dione hydrochloride (v). Yield = 0.115 g (quant.)**. ^1^H-NMR (CH_3_OD, 400 MHz) *δ* 3.18 (dd, *J*_*1*_ *=* 9.0 Hz, *J*_*2*_ = 14.0 Hz*,* 1H), 3.39 (brs, 2H), 3.44 (dd, *J*_*1*_ = 4.0 Hz, *J*_*2*_ = 14.0 Hz*,* 1H), 4.22 (brs, 2H), 4.75 (dd, *J*_*1*_ = 4.0 Hz, *J*_*2*_ = 9.5 Hz*,* 1H), 6.95 (d, *J =* 8.0 Hz, 2H), 7.26 (d, *J* = 8.0 Hz, 2H). MS [ESI+] m/z 304.1 [M + H]^+^.

**2-amino-4-(4-fluorophenyl)thiazole-5-carbonitrile (vi) (****https://doi.org/10.1021/acs.jmedchem.7b00032****)**. To a solution of 4-fluorobenzoylacetonitrile (0.47 g, 2.89 mmol) in dry ethanol (6 mL), dry pyridine (0.24 mL, 2.89 mmol) was added and the mixture was stirred at 70°C for 20 min and then cooled to rt. A previously stirred suspension of thiourea (0.44 g, 5.79 mmol) and iodine (0.73 g, 2.89 mmol) in dry ethanol (4 mL) was slowly added and the mixture was stirred at rt for 2h. Cold water (40 mL) was added and the resulting precipitate was filtered, washed with water (10 mL) and hexane (15 mL) and dried in vacuum to afford the desired product as a yellow solid. Yield = 0.63 g (quant). ^1^H-NMR (dmso-d_6_, 400 MHz) *δ* 7.37 (t, *J* = 8.9 Hz, 2H), 7.95–8.00 (m, 2H), 8.25 (s, 2H). MS [ESI+] m/z 220.0 [M + H]^+^.

**2-chloro-4-(4-fluorophenyl)thiazole-5-carbonitrile (vii) (****https://doi.org/10.1021/acs.jmedchem.7b00032****)**. To a solution of anhydrous CuCl_2_ (0.47 g, 3.47 mmol) in dry CH_3_CN (6.5 mL) was added dropwise *tert*-butoxy nitrite (0.45 g, 4.34 mmol) and the mixture was stirred at rt for 45 min. Then, compound **vi** (0.63 g, 2.89 mmol) was added in portions and stirring was continued for further 2h. The reaction mixture was carefully quenched with 1N HCl (10 mL) and stirred for 15 min. The organic phase was separated, the aqueous phase was extracted with ethyl acetate (20 mL) and the combined organic phase was washed with brine (10 mL), dried (Na_2_SO_4_), filtered and concentrated in vacuum. The crude product was filtered on a silica plug eluted with dichloromethane. Solvents were distilled in vacuum and the residue was triturated with hexane, filtered and dried. The product is isolated as a bright orange thick solid. Yield = 0.49 g (71%). ^1^H-NMR (CDCl_3_, 400 MHz) *δ* 7.19–7.25 (m, 2H), 8.12–8.17 (m, 2H). MS [ESI+] m/z 240.0 [M + H]^+^.

**Synthesis of compounds 1 and 4.** Compounds **iv** or **v** (0.25 g, 0.83 mmol) and **vii** (0.18 g, 0.76 mmol) were dissolved in dry DMSO (7 mL), DIPEA (0.33 mL, 1.89 mmol) was added and the mixture was stirred at 100°C for 8 h and at rt overnight. Water (15 mL) was added and the mixture was extracted with dichloromethane (2 × 30 mL). The combined organic phase was washed with water (2 × 20 mL) and brine (20 mL), dried (Na_2_SO_4_), filtered and concentrated in vacuum. The residue was purified by flash column chromatography eluted with hexane:ethyl acetate (7:3 to 1:1) furnishing the final products.

**(*E*)-2-((2-(4-((2,4-dioxothiazolidin-5-ylidene)methyl)phenoxy)ethyl)amino)-4-(4-fluorophenyl)thiazole-5-carbonitrile (1)**. Light brown solid. Yield = 0.34 g (95%). ^1^H-NMR (dmso-d_6_, 400 MHz) *δ* 3.80–3.81 (m, 2H), 4.27–4.28 (m, 2H), 7.15 (d, J = 8.2 Hz, 2H), 7.36 (t, J = 8.6 Hz, 2H), 7.56 (d, J = 8.3 Hz, 2H), 7.76 (s, 1H), 7.98–8.02 (m, 2H), 8.99–9.02 (m, 1H), 12.50 (s, 1H). ^13^C-NMR (CDCl_3_, 100 MHz) *δ* 44.1, 66.6, 84.1, 115.6, 115.9, 116.2, 116.4, 121.0, 126.3, 129.5, 129.6, 130.3, 130.4, 132.2, 132.6, 160.0, 160.4, 162.1, 164.6, 167.9, 168.4, 170.2. HRMS (ESI): *m*/*z* 467.0652 [M + H]^+^, [Calc. 467.0648].

**2-((2-(4-((2,4-dioxothiazolidin-5-yl)methyl)phenoxy)ethyl)amino)-4-(4-fluorophenyl)thiazole-5-carbonitrile (4)**. Off-yellow amorphous solid. Yield = 0.20 g (56%). ^1^H-NMR (CDCl_3_, 400 MHz) *δ* 3.14–3.20 (m, 1H), 3.42–3.47 (m, 1H), 3.81–3.85 (m, 2H), 4.21–4.23 (m, 2H), 4.50–4.54 (m, 1H), 6.43 (brs, 1H), 6.89 (d, J = 8.3 Hz, 2H), 7.15–7.20 (m, 4H), 8.05–8.08 (m, 3H). ^13^C-NMR (CDCl_3_, 100 MHz) *δ* 37.6, 45.3, 53.4, 65.8, 77.2, 114.5, 114.8 (2C), 115.9 (2C), 116.1, 128.5, 130.0, 130.1, 130.7 (2C), 157.6, 159.5, 162.1, 169.8, 170.0, 173.6. HRMS (ESI): *m*/*z* 469.0811 [M + H]^+^, [Calc. 469.0804].

#### Synthesis of compounds 2 and 5

**4-(2-bromoethoxy)benzaldehyde (viii) (****https://doi.org/10.1002/anie.202105103****)**^159^. 4-hydroxybenzaldehyde (0.75 g, 6.14 mmol) was dissolved in dry CH_3_CN (45 mL) and 1,2-dibromoethane (5.29 mL, 61.4 mmol) and K_2_CO_3_ (1.55 g, 11.2 mmol) were subsequently added. The mixture was stirred under reflux for 20 h, cooled to rt, water (45 mL) was added and the mixture was extracted with Et_2_O (2 × 30 mL). The combined organic phase was washed with brine (25 mL), dried (Na_2_SO_4_), filtered and concentrated in vacuum. The residue was recrystallized from Et_2_O:hexane to give the product as a white solid. Yield = 0.92 g (65%). ^1^H-NMR (CDCl_3_, 400 MHz) *δ* 3.69 (td, *J*_*1*_ = 1.7 Hz, *J*_*2*_ = 6.2 Hz, 2H), 4.40 (td, *J*_*1*_ = 1.7 Hz, *J*_*2*_ = 6.2 Hz, 2H), 7.04 (dd, *J*_*1*_ = 1.7 Hz, *J*_*2*_ = 8.7 Hz, 2H), 7.87 (dd, *J*_*1*_ = 1.9 Hz, *J*_*2*_ = 8.7 Hz, 2H), 9.92 (s, 1H). MS [ESI+] m/z 229.9 [M + H]^+^.

**1-(*tert*-butyl) 4-(3,5-dichlorobenzyl) piperazine-1,4-dicarboxylate (x) (****https://doi.org/10.1021/acsmedchemlett.7b00312****)**. To a solution of (3,5-dichlorophenyl)methanol (1.50 g, 8.47 mmol) in dry DMF (15 mL) was added CDI (1.92 g, 11.86 mmol) and the reaction was stirred at 45°C for 2h. Then, 1-boc-piperazine (1.97 g, 10.59 mmol) was added and the reaction mixture was stirred at rt overnight. Water (30 mL) was added to the mixture and the precipitate was filtered, washed with water (2 × 10 mL) and hexane (10 mL) and dried. The crude product (white solid) was used immediately in the next step without further purification. Yield = 3.30 g (74%). MS [ESI+] m/z 390.1 [M + H]^+^.

**3,5-Dichlorobenzyl piperazine-1-carboxylate hydrochloride (ix) (****https://doi.org/10.1021/acsmedchemlett.7b00312****)**. 4N HCl in dioxane (16 mL, 63 mmol) was added to 1-(*tert*-butyl) 4-(3,5-dichlorobenzyl) piperazine-1,4-dicarboxylate (**xiii**, 2.44 g, 6.27 mmol) at 0°C and the reaction mixture was stirred at rt for 3h. The solvent was evaporated under reduced pressure and the white solid remaining was used in the next step without further purification. Yield = 2 g (quant.). ^1^H-NMR (CDCl_3_, 400 MHz) *δ* 3.09 (m, 4H), 3.65 (m, 4H), 5.10 (s, 2H), 7.47 (s, 2H), 7.57 (s, 1H), 9.49 (brs, 2H). MS [ESI+] m/z 326.1 [M + H]^+^.

**3,5-Dichlorobenzyl 4-(2-(4-formylphenoxy)ethyl)piperazine-1-carboxylate (xi)**. A mixture of compounds **viii** (0.92 g, 4.02 mmol), **ix** (0.44 g, 4.42 mmol) and NaHCO_3_ (1.35 g, 16.08 mmol) in dry DMF (20 mL) was stirred at 80°C for 24 h and at 55°C for 12 h. Then water (50 mL) was added, and the mixture was extracted with ethyl acetate (3 × 25 mL). The combined organic phase was washed with water (25 mL) and brine (25 mL), dried (Na_2_SO_4_), filtered and concentrated in vacuum. The residue was purified by flash column chromatography eluted with hexane:EtOAc (7:3 to 100% ethyl acetate), affording a yellowish oil which solidifies upon standing in the fridge. Yield = 1.76 g (71%). ^1^H-NMR (dmso-d_6_, 400 MHz) *δ* 2.50–2.52 (m, 3H), 2.78 (t, J = 5.6 Hz, 2H), 2.97 (t, J = 5.6 Hz, 1H), 3.46 (m, 4H), 4.27 (t, J = 5.6 Hz, 2H), 5.11 (s, 2H), 7.12 (d, J = 8.4 Hz, 2H), 7.42 (s, 2H), 7.66 (d, J = 8.1 Hz, 2H), 7.70 (s, 1H), 9.90 (s, 1H). MS [ESI+] m/z 437.4 [M + H]^+^.

**3,5-Dichlorobenzyl (*E*)-4-(2-(4-((2,4-dioxothiazolidin-5-ylidene)methyl)phenoxy)ethyl)piperazine-1-carboxylate (2)**. In an oven-dried round-bottom flask, compound **xi** (1.68 g, 3.85 mmol) and 2,4-thiazolidinedione (0.54 g, 4.62 mmol) were dispersed in dry toluene (16 mL). Then, piperidine (0.20 mL, 1.92 mmol) was added followed by acetic acid (0.11 mL, 1.92 mmol) and the mixture was refluxed overnight. The reaction mixture was left to cool at rt where a brownish solid precipitated. The mixture was filtered, washed with toluene (20 mL) and hexane (20 mL) and dried at 45°C overnight. Off-yellow powder. Yield = 2 g (quant.). ^1^H-NMR (dmso-d_6_, 400 MHz) *δ* 2.50–2.52 (m, 3H), 2.76 (t, *J* = 5.6 Hz, 2H), 2.99 (t, *J* = 5.6 Hz, 1H), 3.42 (brm, 4H), 4.17 (t, *J* = 5.6 Hz, 2H), 5.08 (s, 2H), 7.10 (d, *J* = 8.4 Hz, 2H), 7.42 (s, 2H), 7.53 (s, 1H), 7.56 (d, *J* = 8.1 Hz, 2H), 7.70 (s, 1H). ^13^C-NMR (dmso-d_6_, 100 MHz) *δ* 44.0 (2C), 53.2 (2C), 56.9, 65.2, 66.0, 115.3 (2C), 122.5, 126.6 (2C), 127.9, 128.9, 131.0 (2C), 134.2, 134.5 (2C), 141.8, 154.5, 158.6, 176.2, 183.5. HRMS (ESI): *m*/*z* 536.0815 [M + H]^+^, [Calc. 536.0814].

**3,5-dichlorobenzyl4-(2-(4-((2,4-dioxothiazolidin-5-yl)methyl)phenoxy)ethyl)piperazine-1-carboxylate (5)**. The thiazolidinone derivative **2** (0.45 g, 0.84 mmol) was mixed with water (25 mL), to which 5 drops of 0.5M aqueous solution of NaOH was added until pH 11. Then, a mixture of THF:DMF 2:1 (20 mL) was added followed by CoCl_2_^.^6H_2_O (0.128 g, 0.537 mmol), dimethylglyoxime (0.129 g, 1.107 mmol) and sodium borohydride (0.374 g, 9.88 mmol). The reaction mixture was stirred at room temperature for 24h, where a partial conversion of the starting material to the desired product was observed by TLC and MS. Then, more CoCl_2_^.^6H_2_O (0.128 g, 0.537 mmol), dimethylglyoxime (0.129 g, 1.107 mmol) and sodium borohydride (0.374 g, 9.88 mmol) were added and the mixture was stirred overnight. The pH of the reaction was adjusted to 3 with 6N HCl and then to 7–8 by adding 1N NaOH. It was extracted with ethyl acetate (2 × 50 mL), the combined organic phase was washed with water (30 mL) and brine (30 mL), dried (Na_2_SO_4_), filtered and concentrated in vacuum. The residue was purified by flash column chromatography eluted with ethyl acetate, providing the desired product as a slightly yellow semisolid. Yield = 0.226 g (50%). ^1^H-NMR (CDCl_3_, 400 MHz) *δ* 2.64 (brm, 4H), 2.87–289 (m, 2H), 3.09–3.14 (m, 1H), 3.40–3.45 (dd, *J*_*1*_ = 3.8 Hz, *J*_*2*_ = 14.2 Hz, 1H), 3.57–3.60 (m, 4H), 4.10–4.15 (m, 2H), 4.45 (dd, *J*_*1*_ = 3.9 Hz, *J*_*2*_ = 9.2 Hz, 1H), 5.09 (s, 2H), 6.85 (d, *J* = 8.6 Hz, 2H), 7.16 (d, *J* = 8.6 Hz, 2H), 7.24 (s, 2H), 7.28 (s, 1H), 7.33 (s, 1H). ^13^C-NMR (CDCl_3_, 100 MHz) *δ* 37.7, 53.0, 53.7 (2C), 57.1, 60.4, 65.4, 65.6 (2C), 114.8 (2C), 126.1 (2C), 128.1, 128.2, 130.5 (2C), 135.1 (2C), 140.0, 154.7, 157.8, 170.5, 174.3. HRMS (ESI): *m*/*z* 538.0973 [M + H]^+^, [Calc. 538.0970].

#### Synthesis of compound 3

**2-(hydroxymethyl)-2,5,7,8-tetramethylchroman-6-ol (xii) (****https://doi.org/10.1248/cpb.48.272****)**. Lithium aluminum hydride (LiAlH_4_, 0.194 g, 5.12 mmol) was suspended in dry THF (4 mL), and a solution of trolox (0.40 g, 1.60 mmol) in dry THF (6 mL) was added dropwise at room temperature under Argon. The mixture was stirred for 2.5 h and carefully quenched with cold water (10 mL). The aqueous layer was extracted with ethyl acetate (2 × 40 mL), the organic phase was washed with brine (25 mL), dried, filtered and concentrated in vacuum to give the desired product as a white solid. Yield = 0.24 g (63%).^1^H-NMR (CDCl_3_, 400 MHz) *δ* 1.22 (s, 3H), 1.73 (ddd, *J*_*1*_ = 4.7 Hz, *J*_*2*_ = 9.6 Hz, *J*_*3*_ = 16.5 Hz, 1H), 1.99 (ddd, *J*_*1*_ = 7.3 Hz, *J*_*2*_ = 9.4 Hz, *J*_*3*_ = 17.1 Hz, 1H), 2.12 (s, 3H), 2.14 (s, 3H), 2.21 (s, 3H), 2.62–2.71 (m, 2H), 3.62 (d, *J* = 11.1 Hz, 1H), 3.67 (d, *J* = 11.3 Hz, 1H).

**(6-((*tert*-butyldimethylsilyl)oxy)-2,5,7,8-tetramethylchroman-2-yl)methanol (xiii)**. A suspension of sodium hydride (0.085 g of 60% oil dispersion, 2.133 mmol) in dry tetrahydrofuran (1.50 mL) was cooled at 0°C, and then a solution of the diol **xii** (0.24 g, 1.016 mmol) in dry THF (1 mL) was added dropwise under Argon. The mixture was stirred at the same temperature for 45 min, and then a solution of *tert*-butyldimethylsilyl chloride (0.199 g, 1.321 mmol) in dry THF (1 mL) was added dropwise at the same temperature. The reaction mixture was slowly warmed to room temperature and stirred for 3h. Upon completion, the mixture was quenched with cold water (5 mL) and extracted with hexane (2 × 20 mL). The combined organic phase was washed with brine (10 mL), dried, filtered and concentrated in vacuum. The residue was purified by flash column chromatography eluted hexane:EtOAc (97:3) to furnish the desired intermediate as a pale-yellow oil. Yield = 0.179 g (50%). ^1^H-NMR (CDCl_3_, 400 MHz) *δ* 0.15 (s, 6H), 1.07 (s, 9H), 1.25 (s, 3H), 1.70–1.79 (m, 1H), 1.88 (brs, 1H), 1.97–2.11 (m, 1H), 2.11 (s, 3H), 2.13 (s, 3H), 2.15 (s, 3H), 2.62–2.68 (m, 2H), 3.63 (d, *J* = 11.5 Hz, 2H).

**4-((6-Hydroxy-2,5,7,8-tetramethylchroman-2-yl)methoxy)benzaldehyde (xiv)**. To a suspension of potassium *tert*-butoxide (*t*-BuOK, 0.063 g, 0.562 mmol) in dry DMF (0.5 mL), a solution of the alcohol **xiii** (0.179 g, 0.511 mmol) in dry DMF (0.5 mL) was added dropwise at room temperature and under Argon. After stirring for 1 h, 4-fluorobenzaldehyde was added and the mixture was heated at 80°C for 6 h and at room temperature overnight. The reaction mixture was quenched with water (15 mL) and extracted with ethyl acetate (2 × 20 mL). The combined organic extracts were washed with water (15 mL) and brine (15 mL), dried, filtered and concentrated in vacuum. The residue was purified by flash column chromatography eluted with hexane:EtOAc (3:1) to furnish the desired (unprotected) intermediate as a pale-yellow oil, which was used directly in the next step. Yield = 0.055 g (32%). MS [ESI+] m/z 340.43 [M + H]^+^.

**3-(4-Fluorobenzyl)thiazolidine-2,4-dione (xv) (****https://doi.org/10.1021/acs.jmedchem.8b00935****)**. A solution of thiazolidine-2,4-dione (1.17 g, 9.99 mmol) in DMF (5 mL), under Ar, was cooled to 0°C, and sodium hydride (60% mineral oil dispersion, 0.36 g, 8.99 mmol) and a solution of 4-fluorobenzyl chloride (0.793 mL, 0.963 g, 6.66 mmol) in dry DMF (3 mL) were added. The reaction mixture was allowed to warm to room temperature over 5 h. The reaction mixture was then poured over crushed ice (50 mL), hexane (25 mL) was then added, and the product was allowed to crystallize overnight at 4°C. The desired intermediate was collected as colourless needles via vacuum filtration and dried. Yield = 0.952 g (63%). ^1^H-NMR (CDCl_3_, 400 MHz) *δ* 3.92 (s, 2H), 4.75 (s, 2H), 7.00–7.05 (m, 2H), 7.40–7.41 (m, 2H).

**(*E*)-3-(4-fluorobenzyl)-5-(4-((6-hydroxy-2,5,7,8-tetramethylchroman-2-yl)methoxy)benzylidene)thiazolidine-2,4-dione (3)**. In an oven-dried microwave vial employed with a magnetic stirrer, the aldehyde **xiv** (0.051 g, 0.150 mmol) and the 2,4-thiazolidinedione derivative **xv** (0.054 g, 0.240 mmol) were placed followed by dry toluene (1 mL), piperidine (14.8 μL, 0.013 g, 0.023 mmol) and acetic acid (1.33 μL, 1.4 mg, 0.023 mmol). The mixture was stirred under reflux (111°C) for 72h. The mixture was cooled to room temperature overnight, where a yellow solid precipitated. The solid was filtered, washed with toluene and hexane and dried at 40°C overnight. Yellow semisolid. Yield = 11 mg (13.3%).^1^H-NMR (CDCl_3_, 400 MHz) *δ* 1.48 (s, 3H), 1.99 (s, 3H), 2.02 (s, 3H), 2.14 (s, 3H), 2.68–2.72 (m, 2H), 3.64–3.73 (m, 2H), 4.30–4.37 (m, 1H), 4.88 (s, 2H), 5.32 (s, 1H), 6.86 (d, J = 8.8 Hz, 2H), 7.01–7.05 (m, 3H), 7.42–7.47 (m, 4H), 7.87 (s, 1H). HRMS (ESI): *m*/*z* 547.1838 [M + H]^+^, [Calc. 547.1829].

#### Synthesis of EL244

***tert*-butyl 4-(2-bromoethyl)piperidine-1-carboxylate (xvi) (****https://doi.org/10.1039/C6RA03841G****)**. In an oven-dried microwave vial employed with magnetic stirrer, *N*-Boc-4-piperidinethanol (0.521 g, 2.274 mmol) is dissolved in dichloromethane (10 mL). Then triphenylphosphine (0.835 g, 3.184 mmol) and carbon tetrabromide (1.207 g, 3.640 mmol) were added in portions, and the reaction mixture was stirred at room temperature for 72h. Upon completion, the solvent was evaporated and the residue was purified by silica gel flash column chromatography eluted with hexane:ethyl acetate (100% hexane to 5% ethyl acetate in hexane). Colourless oil. Yield: = 0.530 g (79%). ^1^H-NMR (CDCl_3_, 400 MHz) *δ* 1.05–1.17 (m, 3H), 1.47 (s, 9H), 1.65–1.71 (m, 3H), 1.75–1.80 (m, 2H), 2.62–2.71 (m, 2H), 3.47 (t, *J* = 7.0 Hz, 1H), 4.00–4.14 (m, 2H). MS [ESI+] m/z 292.22 [M + H]^+^.

***tert*-butyl 4-(2-(4-formylphenoxy)ethyl)piperidine-1-carboxylate (xvii)**. In a round-bottom flask with magnetic stirrer, *tert*-butyl 4-(2-bromoethyl)piperidine-1-carboxylate (**xvi**) (0.530 g, 1.814 mmol), 4-hydroxybenzaldehyde (0.277 g, 2.268 mmol), cesium carbonate (1.478 g, 4.535 mmol) and dry DMF (3 mL) were consecutively added and the reaction mixture was stirred at 70°C for 6h and at room temperature overnight. Water (10 mL) was added and the mixture was extracted with ethyl acetate (3 × 20 mL). The combined extracts were washed with water (2 × 15 mL), saturated aqueous solution of sodium carbonate (2 × 10 mL) and brine (15 mL), dried (Na_2_SO_4_), filtered and concentrated in vacuum. The product was purified by silica gel flash column chromatography eluted with hexane:ethyl acetate (EtOAc 0–20%). Off-white/yellowish solid. Yield = 0.535 g (88%).^1^H-NMR (CDCl_3_, 400 MHz) *δ* 1.17–1.28 (m, 2H), 1.48 (s, 9H), 1.73–1.82 (m, 5H), 2,74 (t, *J* = 11.8 Hz, 2H), 4.12 (t, *J* = 6.2 Hz, 4H), 7.01 (d, *J* = 8.7 Hz, 2H), 7.86 (d, *J* = 8.8 Hz, 2H), 9.91 (s, 1H). MS [ESI+] m/z 333.43 [M + H]^+^.

**4-(2-(piperidin-4-yl)ethoxy)benzaldehyde (xviii)**. *Tert*-butyl 4-(2-(4-formylphenoxy)ethyl)piperidine-1-carboxylate (**xvii**) (0.531 g, 1.593 mmol) was dissolved in dry dichloromethane (4 mL) in a round-bottom flask and trifluoroacetic acid (3.50 mL, 5.447 g, 47.776 mmol) was subsequently added. The reaction mixture was stirred at room temperature for 2h, the solvent was evaporated in vacuum, saturated sodium bicarbonate aqueous solution (5 mL) was added and the product was extracted with ethyl acetate (3 × 15 mL). The combined organic phase was washed with brine (15 mL), dried (Na_2_SO_4_), filtered and concentrated in vacuum. Yellowish semisolid. Yield = 0.300 g (81%).^1^H-NMR (dmso-d_6_, 400 MHz) *δ* 1.35–1.43 (m, 2H), 1.74–1.85 (m, 5H), 2.74 (t, *J* = 13.7 Hz, 2H), 3.23 (d, *J* = 12.3 Hz, 2H), 4.11 (t, *J* = 6.0 Hz, 2H), 5.20 (brs, 1H), 7.00 (d, *J* = 8.5 Hz, 2H), 7.85 (d, *J* = 8.7 Hz, 2H), 9.90 (s, 1H). MS [ESI+] m/z 233.32 [M + H]^+^.

**3,5-Dichlorobenzyl 4-(2-(4-formylphenoxy)ethyl)piperidine-1-carboxylate (xix)**. To a solution of 3,5-dichlorobenzyl alcohol (0.284 g, 1.607 mmol) in dry DMF (2.3 mL), carbonyl-*di*-imidazole (CDI, 0.365 g, 2.251 mmol) was added and the mixture was stirred at 45°C for 3h. Then, 4-(2-(piperidin-4-yl)ethoxy)benzaldehyde (**xviii**) (0.300 g, 1.286 mmol) was dissolved in dry DMF (2 mL) and added dropwise to the reaction mixture which was then stirred at 45°C for 3h and at room temperature overnight. Water (15 mL) was added and the mixture was extracted with diethyl ether (3 × 10 mL). The combined organic phase was washed with water (12 mL) and brine (12 mL), dried (Na_2_SO_4_), filtered and concentrated in vacuum. The product was purified by silica gel flash column chromatography eluted with hexane-ethyl acetate (85:15 to 70:30). Off-white semisolid. Yield = 0.533 g (95%). ^1^H-NMR (CDCl_3_, 400 MHz) *δ* 1.24–1.31 (m, 2H), 1.79–1.81 (m, 5H), 2.78–2.93 (brm, 2H), 4.11–4.20 (m, 4H), 5.09 (s, 2H), 7.01 (d, *J* = 8.4 Hz, 2H), 7.25 (s, 2H), 7.32 (s, 1H), 7.86 (d, *J* = 8.5 Hz, 2H), 9.91 (s, 1H). MS [ESI+] m/z 436.33 [M + H]^+^.

**3,5-Dichlorobenzyl (*E*)-4-(2-(4-((2,4-dioxothiazolidin-5-ylidene)methyl)phenoxy)ethyl)piperidine-1-carboxylate (xx)**. In an oven-dried microwave vial employed with a magnetic stirrer, 3,5-dichlorobenzyl 4-(2-(4-formylphenoxy)ethyl)piperidine-1-carboxylate (**xix**) (0.521 g, 1.194 mmol) and 2,4-thiazolidinedione (0.168 g, 1.433 mmol) were placed followed by dry toluene (5 mL), piperidine (59.2 μL, 0.051 g, 0.597 mmol) and acetic acid (34.2 μL, 0.036 g, 0.597 mmol). The mixture was stirred under reflux (111°C) overnight. The mixture was cooled to room temperature, where a yellow solid precipitated. The solid was filtered, washed with toluene and hexane and dried at 45°C overnight. Yellow powder. Yield = 0.500 g (78%).^1^H-NMR (dmso-d_6_, 400 MHz) *δ* 1.65–1.75 (m, 6H), 2.71–2.86 (brm, 2H), 3.00 (t, *J* = 5.7 Hz, 1H), 4.00 (d, *J* = 13.6 Hz, 2H), 4.10 (t, *J* = 5.8 Hz, 2H), 5.07 (s, 2H), 7.10 (d, *J* = 8.8 Hz, 2H), 7.41 (s, 2H), 7.54–7.56 (m, 3H), 7.75 (s, 1H), 12.51 (brs, 1H). MS [ESI+] m/z 536.42 [M + H]^+^.

#### 3,5-dichlorobenzyl 4-(2-(4-((2,4-dioxothiazolidin-5-yl)methyl)phenoxy)ethyl)piperidine-1-carboxylate (EL244)

##### Catalyst

9 mg (0.038 mmol) of CoCl_2_^.^6H_2_O and 49 mg (0.413 mmol) of dimethylglyoxime were dissolved in 0.55 mL DMF under stirring, yielding a clear blue-green solution. It was maintained under stirring at rt until fully consumed.

##### Reducing agent

0.177 g (4.670 mmol) of NaBH_4_ were dissolved in 1.50 mL H_2_O+ 0.5 mL of 0.1 M solution of NaOH under cooling in ice (at 0^o^C). It was maintained in ice until fully consumed.

##### Reaction

23.4 mg of NaOH (0.585 mmol)and subsequently 0.250 g (0.467 mmol) of 3,5-dichlorobenzyl 4-(2-(4-((2,4-dioxothiazolidin-5-yl)methyl)phenoxy)ethyl)piperidine-1-carboxylate (**xx**) were added in 5 mL of H_2_O and the obtained suspension was stirred and heated at 55°C until a solution was formed. Part of the catalyst was added (0.15 mL of CoCl_2_-DMG in DMF) during 1 min into the solution, followed by part of the reducing agent (0.50 mL of NaBH_4_ in H_2_O) during 2 min, and the mixture was stirred at 55°C for 1 h. The same procedure was repeated 3 more times (each time, addition of 1/4 of the catalyst followed by 1/4 of the reducing agent, followed by 1 h stirring at 55°C, i.e., 1 addition every 1 h). On completion of the additions, the mixture was stirred at 45°C overnight. The following day an aqueous solution of 6 N HCl (10 mL) was added. The aqueous phase was extracted with EtOAc (2 × 20 mL). The combined organic phase was washed with water (20 mL) and brine (20 mL), dried, filtered and concentrated in vacuum. The product was purified via silica gel flash column chromatography eluted with hexane:EtOAc 3:2. White crystalline solid. Yield = 0.110 g (44%). ^1^H-NMR (dmso-d_6_, 400 MHz) *δ* 1.05–1.10 (m, 2H), 1.64–1.74 (m, 5H), 2.73–2.85 (brm, 2H), 3.03–3.09 (m, 1H), 3.28 (d, *J* = 4.4 Hz, 1H), 3.97–4.01 (m, 4H), 4.87 (dd, *J*_*1*_ = 9.0 Hz, *J*_*2*_ = 4.3 Hz, 1H), 5.07 (s, 2H), 6.87 (d, *J* = 8.2 Hz, 2H), 7.14 (d, *J* = 8.3 Hz, 2H), 7.41 (s, 2H), 7.56 (s, 1H), 12.01 (brs, 1H). ^13^C-NMR (dmso-d_6_, 100 MHz) *δ* 32.0, 32.6 (2C), 35.6, 36.7, 44.2 (2C), 53.5, 65.0, 65.5, 114.8 (2C), 126.6 (2C), 127.9, 128.9, 130.8 (2C), 134.5 (2C), 141.9, 154.5, 158.1, 172.2, 176.2. HRMS (ESI): *m*/*z* 537.1019 [M + H]^+^, [Calc. 537.1018].

## Supplemental information

Document S1. Figures S1–S9 and Tables S1–S6

Data S1. HDX raw data, related to Figure 5

Data S2. Differentially expressed genes (DEGs) in the lung tissue post-BLM administration (BLM vs. SAL), related to Figure S9

Data S3. Differentially expressed genes (DEGs) in the lung tissue of BLM-treated mice post-EL244 administration (BLM/EL244 vs. BLM), related to Figure S9

Data S4. The expression of BLM DEGs was reversed by EL244, related to Figure S9

Data S5. Venn diagram gene lists showing shared and unique genes across groups, related to Figure S9

Document S2. Article plus supplemental information
