# Supplementary material for: An aerosolized dual-action autotaxin inhibitor-PPARγ agonist for the treatment of pulmonary fibrosis
Source: Cell Rep Med. 2026 Apr 30;7(5):102778. doi: 10.1016/j.xcrm.2026.102778 (PMC13198257; doi:10.1016/j.xcrm.2026.102778)
Supplement: Document S1. Figures S1–S9 and Tables S1–S6 [file mmc1.pdf]

Cell Reports Medicine, Volume *7*

Supplemental information

An aerosolized dual-action autotaxin inhibitor-PPARγ agonist for the treatment of pulmonary fibrosis

Alexios N. Matralis, Elli-Anna Stylianaki, Eleni M. Ladopoulou, Paraskevi Kanellopoulou, Stefanos Smyrniotis, Christiana Magkrioti, Konstantinos D. Papavasileiou, Sabine Willems, Juan Pablo Rincon Pabon, Dimitris Nastos, Alexandros Galaras, Céline Moro, Skarlatos G. Dedos, Eleanna Kaffe, Pantelis Hatzis, Hanan Osman-Ponchet, Daniel Merk, Argyris Politis, Antreas Afantitis, Ioulia Tseti, Katerina M. Antoniou, Athol U. Wells, and Vassilis Aidinis

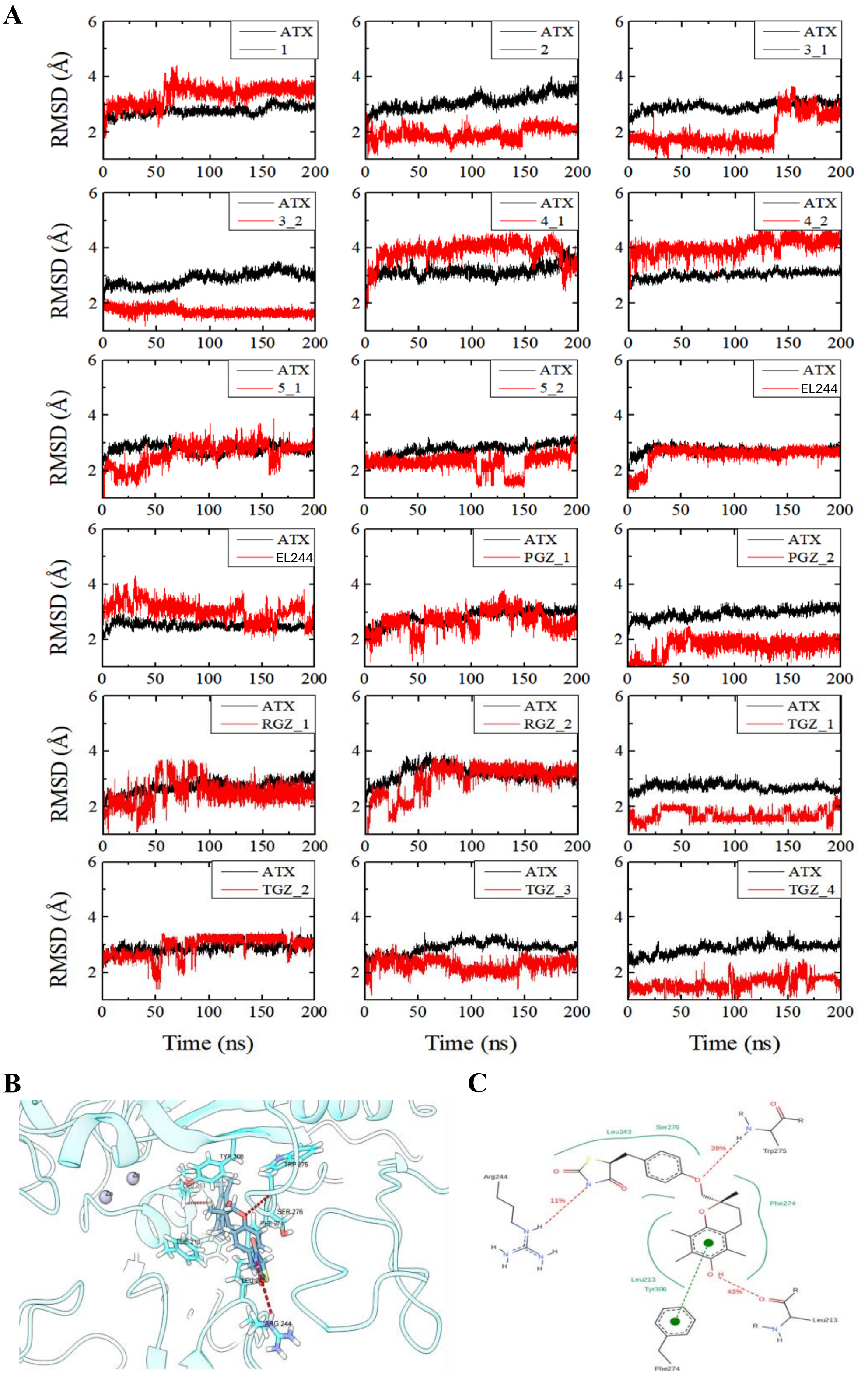

Figure S1. Molecular Dynamics simulations of Autotaxin (ATX) and Troglitazone (TGZ) interactions. Related to Figure 1

(A) Root mean square deviation (RMSD) plots for the ATX protein systems analysed in their complex bound states with TGZ diastereoisomers.

(B) Three- and (C) two- dimensional representations of isomer TGZ_4 in complex with ATX.

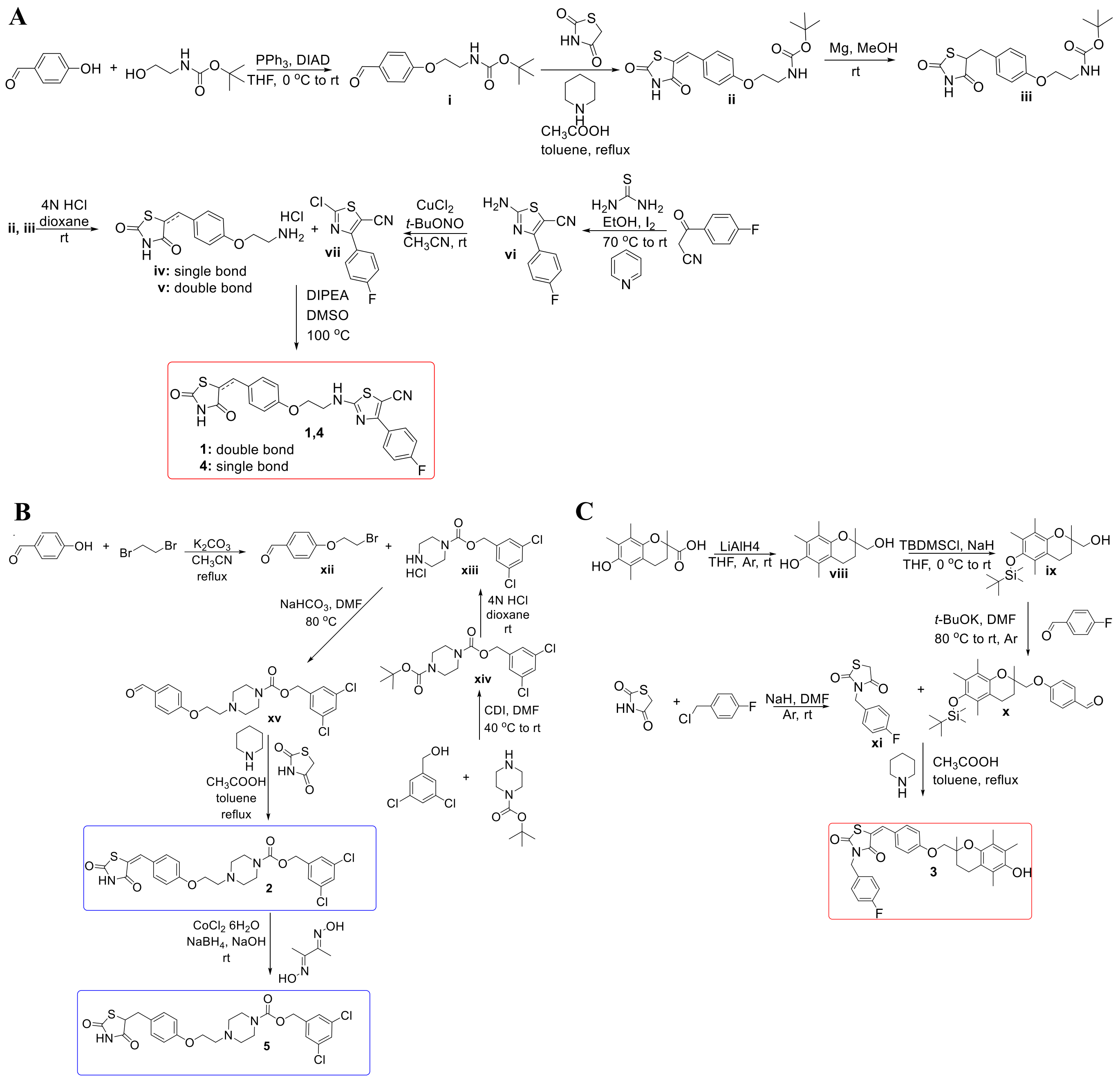

Figure S2. Synthetic route for compounds 1-5. Related to Figure 2

Synthetic routes followed for the synthesis of compounds:

(A) 1 and 4
(B) 2 and 5
(C) 3

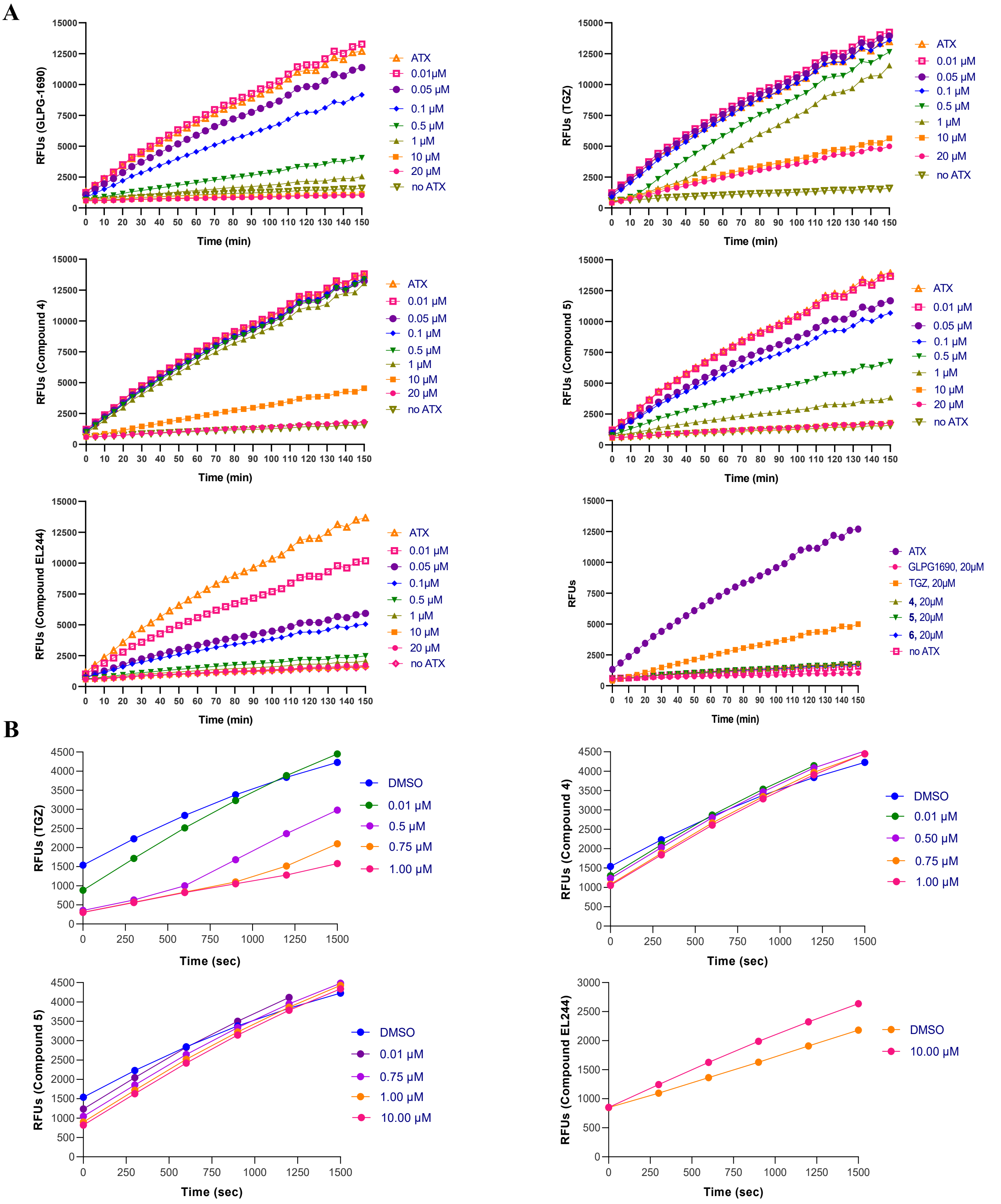

Figure S3. Kinetic analysis of ATX inhibition. Related to Figures 3 and 4
(A) Kinetic graphs of the inhibition of ATX by compounds **4**, **5** and **EL244**, compared with the reference compounds GLPG-1690 and TGZ.
(B) Kinetics for the second (oxidation of choline catalysed by choline oxidase) and third reaction (the conversion of the Amplex substrate to the fluorescent resorufin catalysed by horseradish peroxidase, HRP) of the Amplex Red Lyso-phospholipase D assay for representative compounds (TGZ, **4**, **5** and **EL244**). The first reaction includes the conversion of LPC to LPA catalysed by ATX. Compounds **4**, **5** and **EL244** are not involved at all in the second and third reaction, therefore targeting selectively ATX.
Kinetic graphs are indicative of three technical replicates.

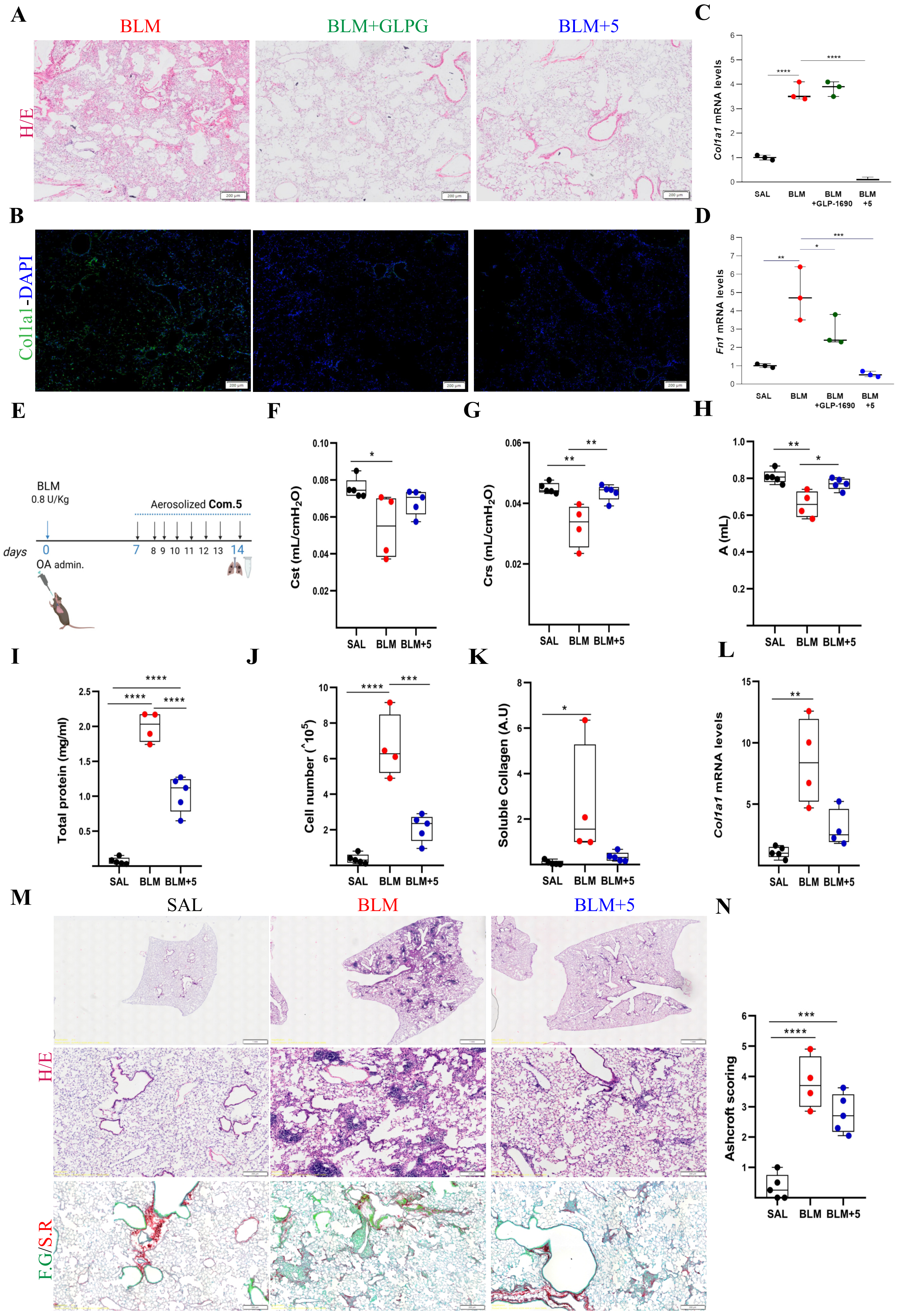

A
BLM
BLM+GLPG
BLM+5
H/E
B
Col1a1-DAPI
C
Col1a1 mRNA levels
SAL
BLM
BLM +GLP-1690
BLM +5
D
Fn1 mRNA levels
E
BLM
0.8 U/Kg
Aerosolized Com.5
days
0
7
8 9 10 11 12 13
14
OA admin.
F
Cst (mL/cmH2O)
G
Crs (mL/cmH2O)
H
A (mL)
I
Total protein (mg/ml)
J
Cell number (^10^5)
K
Soluble Collagen (A.U)
L
Col1a1 mRNA levels
M
SAL
BLM
BLM+5
H/E
F.G/S.R
N
Ashcroft scoring

Figure S4.

Figure S4. Compound 5 attenuates BLM-induced fibrosis. Related to Figure 3
(A-D) Fibrotic precision-cut lung slices (PCLS) were treated with compounds (30 μM) for 72h.
(A) H&E staining in fibrotic slices treated with GLPG1690 and compound 5; scale bars 200 μm.
(B) Immunostaining for COL1a1 (green) and DAPI (blue); scale bars 200 μm.
(C) *Col1a1* and (D) *Fn1* mRNA levels were quantified with Q-RT-PCR; each sample is a pool of three slices (n=3). Values were normalized over the expression of *B2m* housekeeping gene and presented as fold change over control.
(E) Schematic representation of the locally employed BLM model and drug administration; Compound 5 was administered via inhalation at 15 mg/kg, twice daily, from day 7 to day 14 post-BLM administration (day 0). The compound was dissolved in 10% Kolliphor in saline. The vehicle (10% Kolliphor in saline) was administered to both the SAL and BLM control groups.
(F, G, H) Respiratory mechanics assessed with FlexiVent system. Parameters include mean static lung compliance (F), mean respiratory system compliance (G) and mean total lung capacity (H).
(I) Total protein concentration in BALFs, as determined with the Bradford assay (n=5,4,5).
(J) Inflammatory cell numbers in BALFs, as counted with a hematocytometer (n=5,4,5).
(K) Soluble collagen levels in the BALFs were detected with the direct red assay (n=5,4,5).
(L) *Col1a1* mRNA expression was interrogated with Q-RT-PCR (n=5,4,4); values were normalized to the expression of *B2m* and presented as fold change over control.
(M) Representative images of lung sections from mice of the indicated treatment groups, stained with hematoxylin and eosin (H&E) and Fast Green/Sirius Red (F.G./S.R.; green/red); scale bars 1mm ,200μm. (N) Quantification of fibrosis severity in H&E-stained lung sections via Ashcroft scoring (n=5,4,5).
Data in box and whiskers include the median (line), interquartile range (box), and minimum and maximum range (tails); each dot represents a biological replicate. Following normality testing, statistical significance was assessed with one-way ANOVA and Tukey's post-hoc test (C, D, G, H, I, J, K, N) or Welch ANOVA and post-hoc Games-Howell's test (L) or with Kruskal Wallis test and post-hoc Dunn's test (F). $^{*/**/***/****}$ denote $p<0.05/0.01/0.001/0.0001$, respectively.

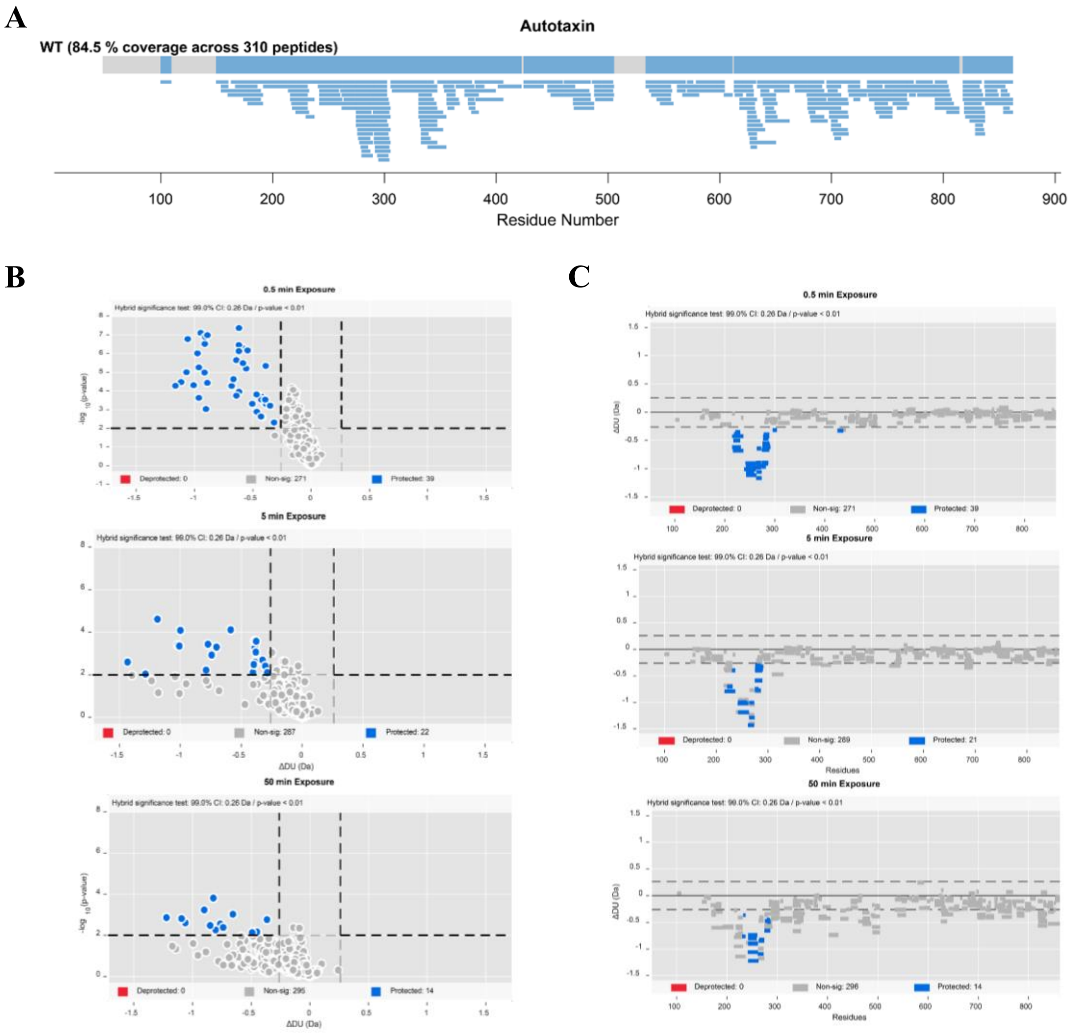

Figure S5. HDX-MS statistical analysis. Related to Figure 5

(A) ATX peptic peptide coverage map.

(B) Volcano plots for the identification of significant differences using $\alpha=0.01$ (see Methods for details). Dots represent differential HDX differences, with blue indicating significant protection and light grey indicating non-significant differences.

(C) Woodsplot representation with statistically significant peptides at each labelling time point. Peptides are represented as rectangles, with blue indicating significant protection and light gray indicating no differences. The x-axis denotes protein residues, while the y-axis shows the magnitude of the differences.

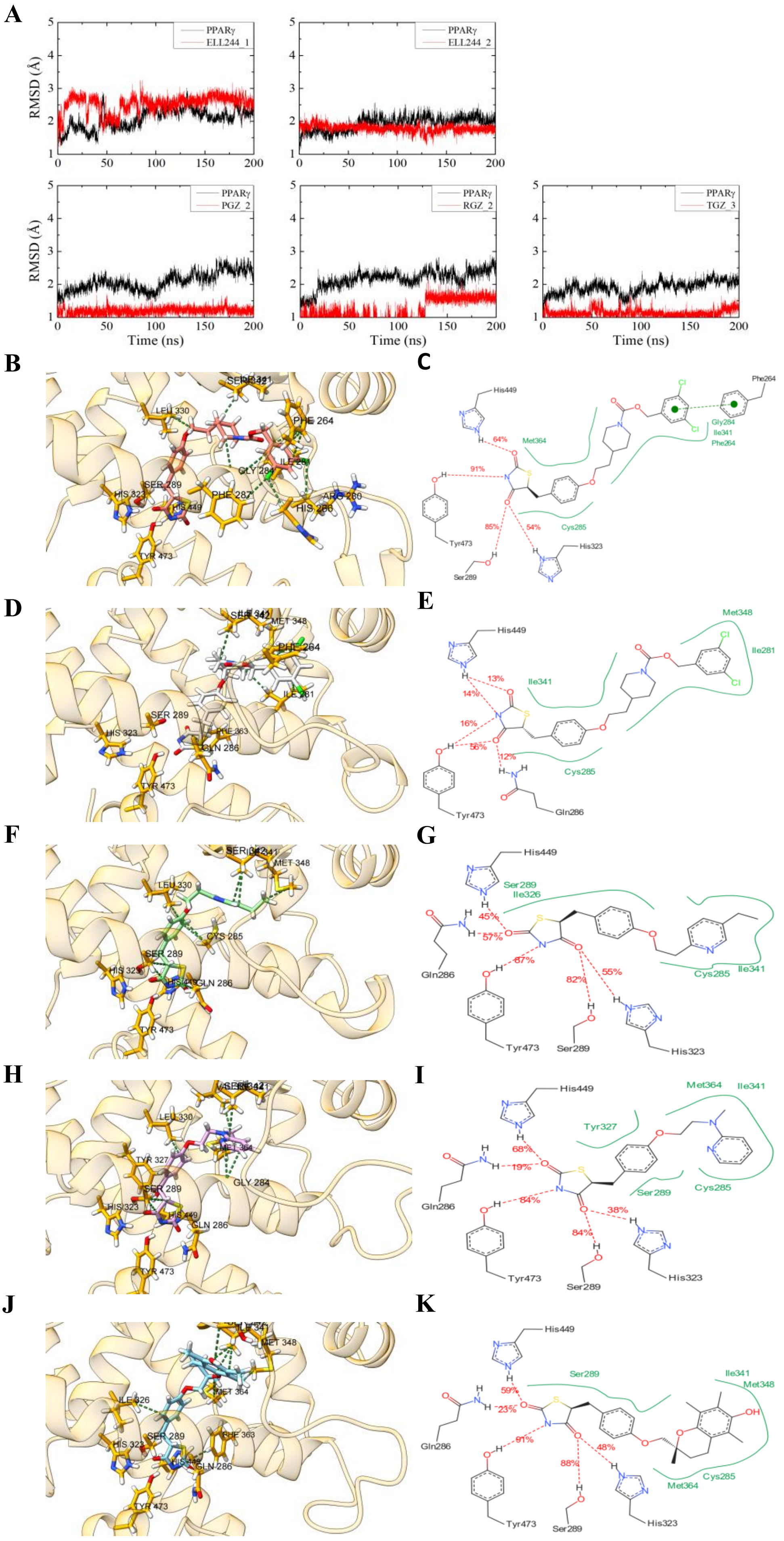
A
RMSD (Å)
Time (ns)
PPARγ
ELL244_1
ELL244_2
PGZ_2
RGZ_2
TGZ_3
B
C
D
E
F
G
H
I
J
K

Figure S6

Figure S6. RMSD and molecular dynamics (MD) simulation analysis of EL244, PGZ, RGZ, and TGZ isomers in complex with PPARγ. Related to Figure 6

(A) RMSD plots of the PPARγ protein systems investigated in their complex bound states with EL 244 and TGZ

(B) Three- and (C) two- dimensional MD simulation representations of the EL244_2-PPARγ complex centroid conformation.

(D) Three- and (E) two- dimensional MD simulation representations of the EL 244_1-PPARγ complex centroid conformation.

(F) Three- and (G) two- dimensional MD simulation representations of the PGZ_2-PPARγ complex centroid conformation.

(H) Three- and (I) two- dimensional MD simulation representations of the RGZ_2-PPARγ complex centroid conformation.

(J) Three- and (K) two- dimensional MD simulation representations of the TGZ_3-PPARγ complex centroid conformation.

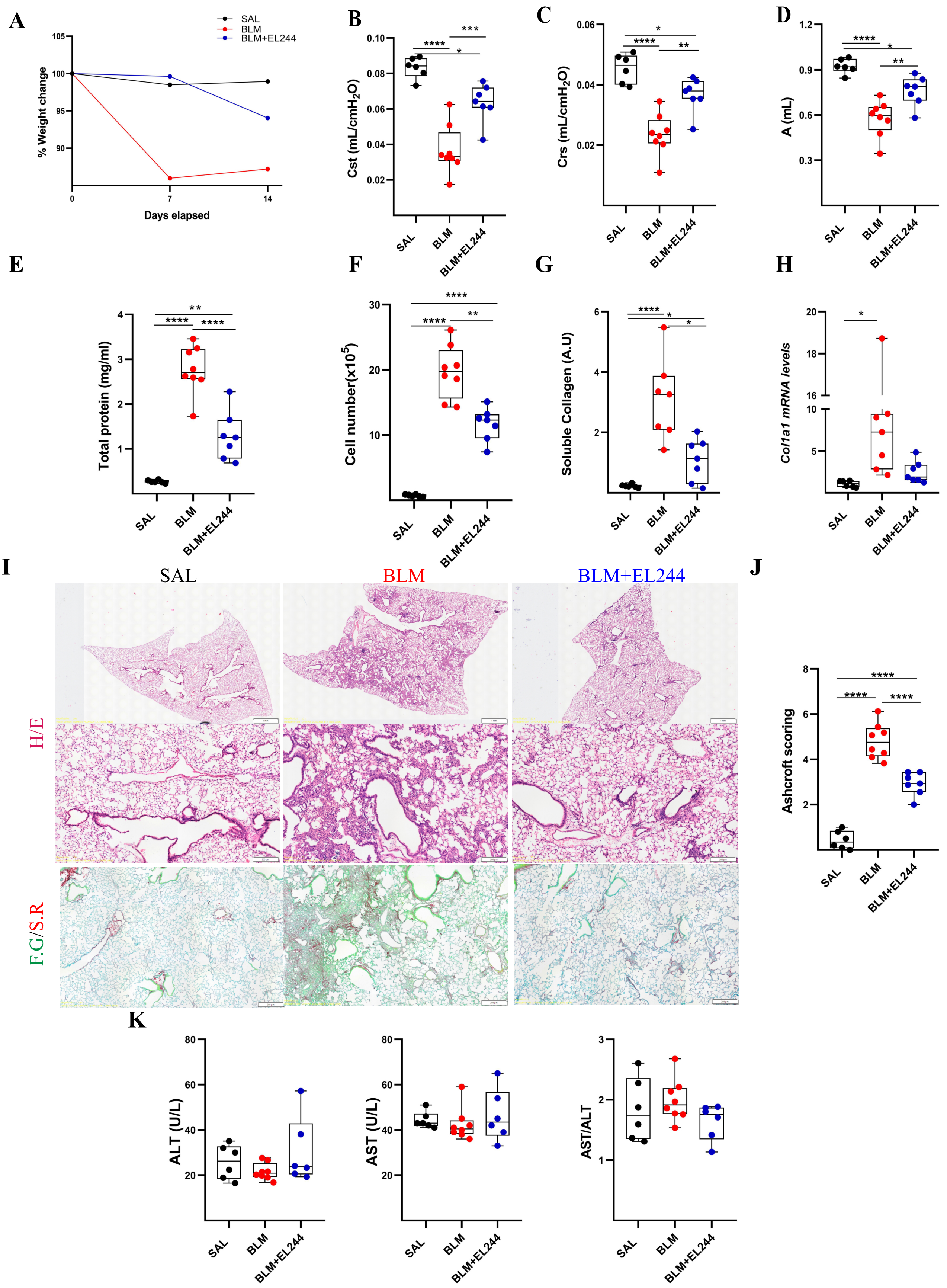
A
SAL
BLM
BLM+EL244
% Weight change
Days elapsed
B
Cst (mL/cmH2O)
C
Crs (mL/cmH2O)
D
A (mL)
E
Total protein (mg/ml)
F
Cell number(x10^5)
G
Soluble Collagen (A.U)
H
Col1a1 mRNA levels
I
H/E
F.G/S.R
J
Ashcroft scoring
K
ALT (U/L)
AST (U/L)
AST/ALT

Figure S7.

Figure S7. Inhaled prophylactic EL244 administration attenuates BLM-induced pulmonary fibrosis. Related to Figure 7

EL244 was administered via inhalation at 15 mg/kg, once daily, from one day before to 14 days after bleomycin (BLM) administration (day 0). The compound was dissolved in 15% Kolliphor in saline. The vehicle (15% Kolliphor in saline) was administered to both the SAL and BLM control groups.

(A) Weight change in the different mouse groups during the 2 weeks of the pulmonary fibrosis experiment.

(B-D) Respiratory mechanics assessed using the FlexiVent system (n=6,8,7). Parameters include mean static lung compliance (B), mean respiratory system compliance (C) and mean total lung capacity (D).

(E) Total protein concentration in BALFs, as determined with the Bradford assay (n=6,8,7).

(F) Inflammatory cell numbers in BALFs, as counted with a hematocytometer (n=6,8,7).

(G) Soluble collagen levels in the BALFs were detected with the direct red assay (n=6,8,7).

(H) *Col1a1* mRNA expression was interrogated with Q-RT-PCR (n=6,7,7); values were normalized to the expression of *B2m* and presented as fold change over control.

(I) Representative images of lung sections from mice of the indicated treatment groups, stained with hematoxylin and eosin (H&E) and Fast Green/Sirius Red (F.G/S.R; green/red); Scale bars 1 mm, 200 μm.

(J) Quantification of fibrosis severity in H&E-stained lung sections via Ashcroft scoring (n=6,8,7).

(K) Serum concentrations of alanine aminotransferase (ALT), aspartate aminotransferase (AST) and the AST/ALT ratio (n=6,8,6). Data in box and whiskers include the median (line), interquartile range (box), and minimum and maximum range (tails); each dot represents a biological replicate.

Following normality testing, statistical significance was assessed with one-way ANOVA and post-hoc Tukey's test (B, C, D, E, K/ALT, K/ALT/AST) or Welch ANOVA and post-hoc Games-Howell test (F, G, H, J) or Kruskal Wallis and post-hoc Dunn's test (K/AST); $^{*/**/***/****}$ denote $p<0.05/0.01/0.001/0.0001$, respectively.

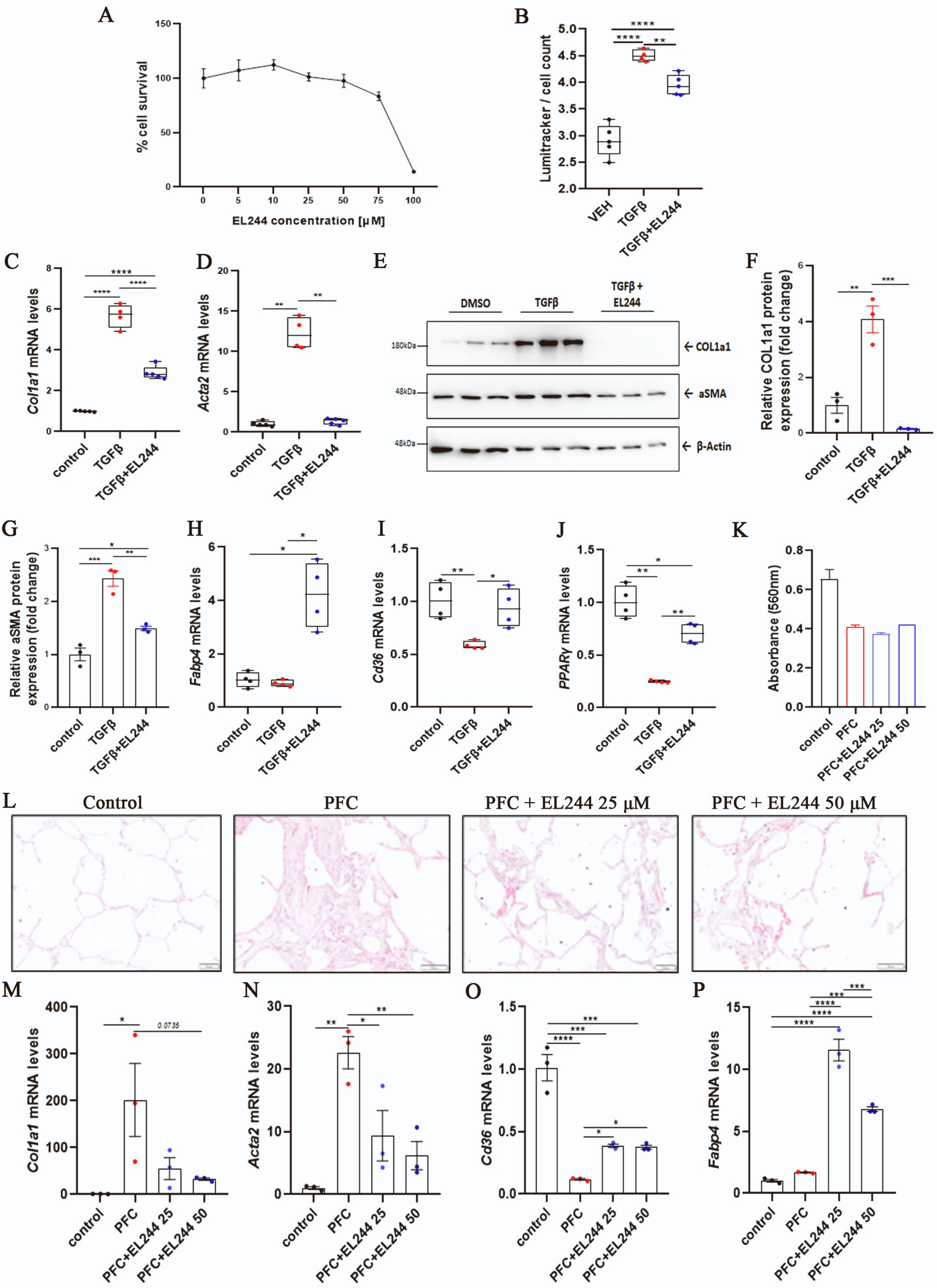

A
% cell survival
EL244 concentration [μM]
B
Lumitracker / cell count
VEH
TGFβ
TGFβ+EL244
C
Col1a1 mRNA levels
D
Acta2 mRNA levels
E
DMSO
TGFβ
TGFβ + EL244
180kDa
48kDa
48kDa
COL1a1
aSMA
β-Actin
F
Relative COL1a1 protein expression (fold change)
G
Relative aSMA protein expression (fold change)
H
Fabp4 mRNA levels
I
Cd36 mRNA levels
J
PPARγ mRNA levels
K
Absorbance (560nm)
control
PFC
PFC+EL244 25
PFC+EL244 50
L
Control
PFC
PFC + EL244 25 μM
PFC + EL244 50 μM
M
Col1a1 mRNA levels
0.0735
N
Acta2 mRNA levels
O
Cd36 mRNA levels
P
Fabp4 mRNA levels

Figure S8

Figure S8. EL244 suppresses fibrotic markers and induces PPARγ target genes in human cells and Precision-Cut Lung Slices (PCLS). Related to Figure 7

(A) MTT in primary normal human lung fibroblasts (NHLFs) with increasing concentrations of **EL244**.
(B) Mitotracker assay in NHLFs. Mitochondria of cells treated with TGFβ (10 ng/ml) and **EL244** (25 μM) for 24 h were labelled with Lumitracker (25 nM). The ratio of fluorescence intensity of Lumitracker to cell number is shown.
(C-J) NHLFs were treated with TGFβ 10ng/ml and **EL244** 75 μM or vehicle.
(C) *Col1a1* mRNA levels in NHLFs in the presence of TGFβ 10ng/ml and **EL244** 75 μM or vehicle as interrogated with Q-RT-PCR; values were normalized to the expression of *Hprt1* and presented as fold change over control (*n*=5,4,5).
(D) *Acta2* mRNA levels in NHLFs in the presence of TGFβ 10ng/ml and **EL244** 75 μM or vehicle as interrogated with Q-RT-PCR; values were normalized to the expression of *Hprt1* and presented as fold change over control (*n*=5,4,5).
(E) Western blots (WB) of COL1a1, aSMA and Actin from NHLFs treated with TGFβ 10ng/ml and **EL244** 75 μM or vehicle.
(F) COL1a1 protein levels quantification from WB (*n*=3).
(G) aSMA protein levels quantification from WB (*n*=3).
(H-J) mRNA expression of selected genes in NHLFs in the presence of TGFβ 10ng/ml and **EL244** 75 μM or vehicle as interrogated with Q-RT-PCR; values were normalized to the expression of *Hprt1* and presented as fold change over control (*n*=4). (H) *Fabp4* mRNA levels, (I) *Cd36* mRNA levels, (J) *Pparγ* mRNA levels.
(K) MTT in human PCLS with or without profibrotic cocktail (PFC) in the presence or absence of **EL244**.
(L-P) Human PCLS were treated with PFC and two concentrations of **EL244** (25 and 50 μM).
(L) H&E staining of slices from human PCLS, scale bars 100 μM.
(M-P) mRNA expression of selected genes in human PCLS as interrogated with Q-RT-PCR; values were normalized to the expression of *Rplp0* and presented as fold change over control (*n*=3). (M) *Col1a1* mRNA levels, (N) *Acta2* mRNA levels, (O) *Cd36* mRNA, (P) *Fabp4* mRNA levels from human PCLS as interrogated with Q-RT-PCR (*n*=3).
Data in box and whiskers include the median (line), interquartile range (box), and minimum and maximum range (tails). Data in bar graphs are presented as means ± SEM. Each dot represents a biological replicate. Following normality testing, statistical significance was assessed with one-way ANOVA and Tukey's post-hoc test (B, C, F, G, I, M, N, O, P) or Welch ANOVA and post-hoc Games-Howell's test (D, H, J). */**/***/**** denote $p<0.05/0.01/0.001/0.0001$, respectively.

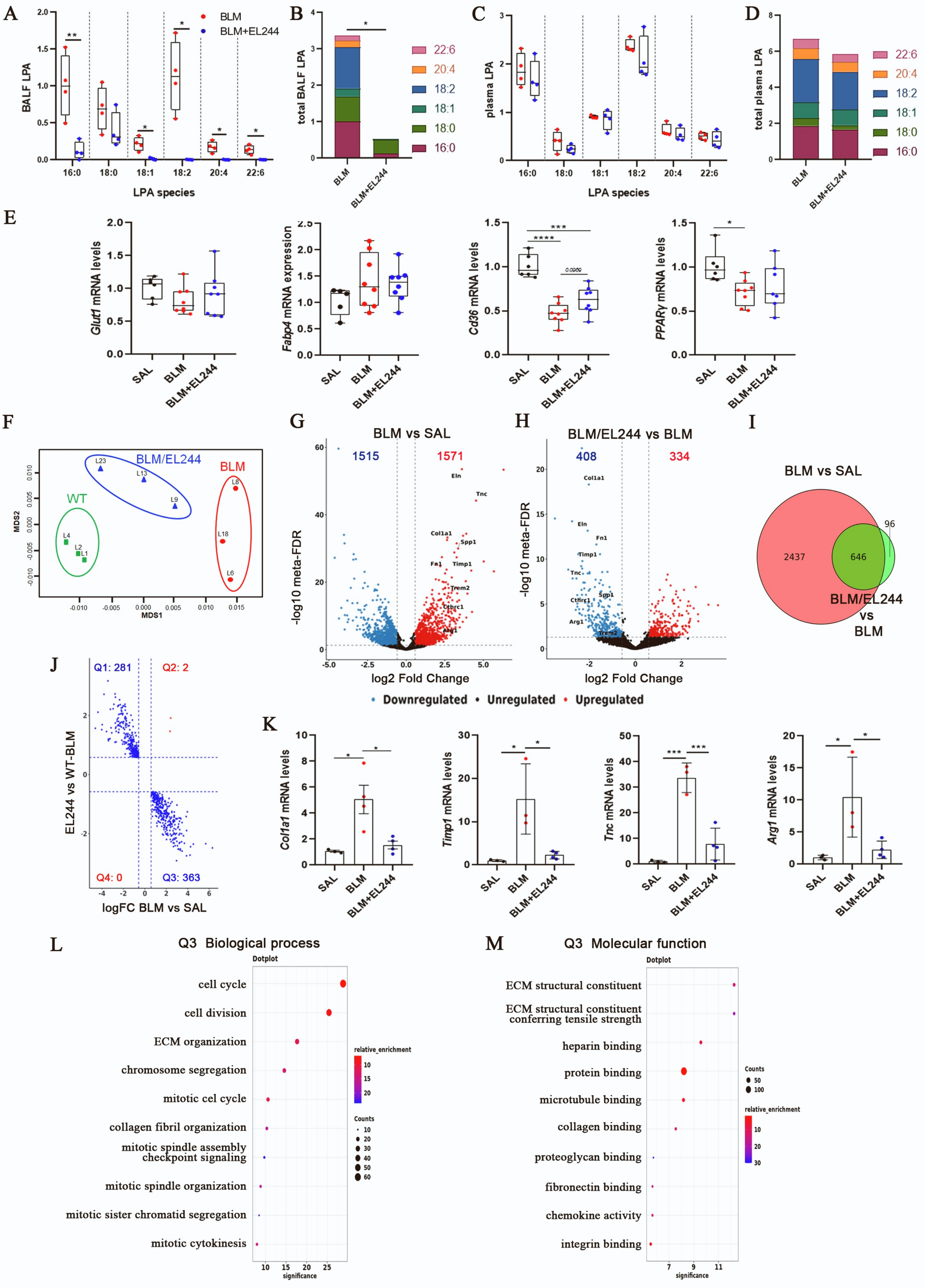
A
BALF LPA
BLM
BLM+EL244
LPA species
16:0
18:0
18:1
18:2
20:4
22:6
B
total BALF LPA
C
plasma LPA
D
total plasma LPA
E
Glut1 mRNA levels
Fabp4 mRNA expression
Cd36 mRNA levels
PPARγ mRNA levels
SAL
F
BLM/EL244
WT
MDS1
MDS2
G
BLM vs SAL
1515
1571
H
BLM/EL244 vs BLM
408
334
-log10 meta-FDR
log2 Fold Change
Downregulated
Unregulated
Upregulated
I
2437
646
96
J
Q1: 281
Q2: 2
Q4: 0
Q3: 363
EL244 vs WT-BLM
logFC BLM vs SAL
K
Col1a1 mRNA levels
Timp1 mRNA levels
Tnc mRNA levels
Arg1 mRNA levels
L
Q3 Biological process
Dotplot
cell cycle
cell division
ECM organization
chromosome segregation
mitotic cel cycle
collagen fibril organization
mitotic spindle assembly checkpoint signaling
mitotic spindle organization
mitotic sister chromatid segregation
mitotic cytokinesis
relative_enrichment
Counts
significance
M
Q3 Molecular function
ECM structural constituent
ECM structural constituent conferring tensile strength
heparin binding
protein binding
microtubule binding
collagen binding
proteoglycan binding
fibronectin binding
chemokine activity
integrin binding

Figure S9

Fig. S9. Mode of action of inhaled EL244 in BLM-induced pulmonary fibrosis. Related to Figure 7

(A) BALF LPA levels as measured with MS/MS.
(B) Total LPA levels in BALFs.
(C) Plasma LPA levels as measured with MS/MS.
(D) Total LPA levels in plasma.
(E) PPARγ target genes and *PPARγ* mRNA levels in the lungs of mice upon **EL244** treatment in the BLM- induced pulmonary fibrosis as interrogated with Q-RT-PCR; values were normalized to the expression of *B2m* and presented as fold change over control. (*n*= 6,8,8 for *Glut1* and *Cd36*, *n*=5,8,8 for *Fabp4* and *n*=6,8,7 for *PPARγ*).
(F) Principal Component Analysis plot for the samples used in the RNA-SEQ.
(G) Volcano plot depicting the differentially expressed genes (DEGs) upon BLM treatment (compared to SAL).
(H) Volcano plot depicting the DEGs upon **EL244** treatment of BLM-treated mice (compared to BLM).
(I) Venn diagram comparing the lists of DEGs following BLM (in red) and **EL244** (in green) treatment.
(J) Scatter plot showing the regulation patterns of the 646 common DEGs across the four indicated quadrants.
(K) Verification of selected genes' expression in the samples used in the RNA-SEQ, as interrogated with Q-RT-PCR; values were normalized to the expression of *B2m* and presented as fold change over control (*n*=3,4,4 for *Col1a1* and *n*=3,3,4 for *Timp1*, *Tnc* and *Arg1*).
(L-M). Dot plots showing the top 10 statistically enriched GO terms associated with the DEGs in Q3 (downregulated by **EL244** and upregulated by BLM).
Data in box and whiskers include the median (line), interquartile range (box), and minimum and maximum range (tails). Data in bar graphs are presented as means ± SEM. Each dot represents a biological replicate. Following normality testing, statistical significance was assessed with an unpaired t-test separately for each LPA species (A-D) or with one-way ANOVA and post-hoc Tukey's test (E, K). */**/***/**** denote $p<0.05/0.01/0.001/0.0001$, respectively.

Table S1. Top-ranked candidate ATX inhibitors. Related to Figure 1

Binding affinities are in kcal mol^{-1} units. % inhibition at 100 μM.

a/a	Compound (Prestw #)	Docking Score	% inhibition (IC50 μM)	a/a	Compound (Prestw #)	Docking Score	% inhibition (IC50 μM)
1	1503	-36.38	63.53 (14.73)	26	627	-25.43	-4.13 (>100)
2	425	-31.95	99.21 (1.60)	27	470	-25.38	70.90 (35.09)
3	400	-31.06	78.24 (2.75)	28	1217	-25.26	26.23 (>100)
4	1726	-30.35	66.92 (53.05)	29	814	-25.20	9.41 (>100)
5	437	-30.16	5.32 (>100)	30	340	-25.15	22.27 (>100)
6	1743	-30.10	59.50 (57.06)	31	539	-25.06	8.74 (>100)
7	656	-29.16	13.38 (>100)	32	1239	-25.04	3.37 (>100)
8	1190	-28.88	26.69 (>100)	33	1350	-24.79	69.33 (62.76)
9	327	-28.73	13.87 (>100)	34	1467	-24.70	99.17 (0.53)
10	991	-28.72	74.27 (>39.38)	35	1796	-24.44	37.06 (>100)
11	862	-28.69	88.64 (3.51)	36	482	-24.34	31.18 (>100)
12	316	-28.50	48.82 (~100)	37	131	-24.19	5.40 (>100)
13	1290	-27.96	46.70 (~100)	38	1737	-24.11	26.64 (>100)
14	939	-27.72	-5.03 (>100)	39	819	-24.00	35.57 (>100)
15	473	-27.19	18.19 (>100)	40	389	-23.73	41.89 (>100)
16	1174	-27.05	33.56 (>100)	41	104	-23.54	6.88 (>100)
17	150	-26.96	26.79 (>100)	42	1270	-23.34	6.72 (>100)
18	143	-26.19	31.78 (>72.5)	43	1798	-23.31	19.95 (>100)
19	1188	-26.06	29.81 (>100)	44	587	-22.97	74.79 (>51.92)
20	1770	-26.01	43.57 (>100)	45	1793	-22.91	-7.94 (>100)
21	869	-25.81	>100	46	1189	-22.64	80.58 (>51.52)
22	1494	-25.79	23.20 (>100)	47	285	-22.55	46.28 (~100)
23	149	-25.62	-7.37 (>100)	48	1292	-22.46	5.90 (>100)
24	1794	-25.55	19.33 (>100)	49	378	-22.20	13.22 (>100)
25	1364	-25.53	65.18 (>12.97)				

Table S2. Per-residue MM–GBSA energy decomposition in the ATX complexes with tested compounds. Related to Figure 1

Only selected residues are shown. Uncertainties denote standard error of the mean (units in kcal mol-1) and are included in parentheses.

Label	Residue	van der Waals		Electrostatic		Polar Solvation		Total energy	
ATX-5_1	Tyr82	-1.82	(0.02)	-0.64	(0.01)	1.32	(0.01)	-1.43	(0.02)
	Phe210	-1.82	(0.01)	-0.04	(0.01)	0.22	(0.00)	-1.85	(0.01)
	Leu213	-0.91	(0.01)	0.92	(0.00)	-0.86	(0.00)	-0.91	(0.01)
	Tyr214	-1.87	(0.01)	0.46	(0.01)	-0.03	(0.00)	-1.62	(0.01)
	Phe249	-2.46	(0.02)	0.01	(0.01)	0.57	(0.01)	-2.19	(0.01)
	Trp254	-3.86	(0.02)	0.33	(0.01)	0.45	(0.01)	-3.57	(0.02)
	Pro258	-0.92	(0.00)	-1.30	(0.01)	1.03	(0.01)	-1.31	(0.01)
	Phe273	-1.81	(0.01)	-1.12	(0.01)	1.49	(0.01)	-1.68	(0.01)
	Phe274	-1.89	(0.01)	-0.18	(0.01)	0.32	(0.01)	-2.02	(0.01)
ATX-5_2	Phe210	-2.82	(0.01)	0.24	(0.01)	0.07	(0.01)	-2.82	(0.01)
	Leu213	-2.04	(0.01)	1.54	(0.00)	-1.68	(0.00)	-2.39	(0.01)
	Ala217	-1.15	(0.01)	0.79	(0.01)	-0.77	(0.00)	-1.23	(0.01)
	Lys248	-1.22	(0.01)	2.77	(0.02)	-3.08	(0.02)	-1.71	(0.01)
	Phe249	-1.88	(0.01)	0.16	(0.00)	0.08	(0.00)	-1.85	(0.01)
	Pro258	-0.58	(0.00)	-2.69	(0.01)	1.60	(0.00)	-1.68	(0.01)
	Leu259	-0.84	(0.00)	-3.81	(0.01)	2.56	(0.01)	-2.12	(0.01)
	Trp260	-1.02	(0.00)	-2.52	(0.01)	1.78	(0.01)	-1.84	(0.01)
	Phe273	-2.51	(0.01)	-1.70	(0.01)	2.56	(0.01)	-1.82	(0.01)
	Phe274	-4.99	(0.01)	-0.50	(0.01)	1.79	(0.01)	-4.29	(0.01)
	Trp275	-1.18	(0.01)	-4.53	(0.02)	4.20	(0.01)	-1.59	(0.01)
	Tyr306	-2.33	(0.01)	-1.88	(0.02)	1.12	(0.01)	-3.31	(0.02)
ATX-EL244_1	Thr209	-1.27	(0.00)	-0.72	(0.01)	1.02	(0.01)	-1.14	(0.00)
	Phe210	-1.61	(0.00)	-0.39	(0.00)	0.69	(0.00)	-1.46	(0.00)
	Leu213	-2.53	(0.00)	-0.90	(0.00)	0.83	(0.00)	-2.86	(0.01)
	Leu243	-0.98	(0.00)	-0.47	(0.00)	0.57	(0.00)	-1.14	(0.00)
	Phe273	-1.62	(0.00)	-0.37	(0.00)	0.70	(0.00)	-1.44	(0.00)
	Phe274	-2.60	(0.01)	-2.49	(0.01)	1.61	(0.00)	-3.74	(0.01)
	Trp275	-1.29	(0.00)	-1.32	(0.01)	0.74	(0.00)	-1.97	(0.00)
	Tyr306	-3.66	(0.01)	-1.10	(0.00)	1.59	(0.00)	-3.46	(0.01)
ATX-EL244_2	Phe210	-1.46	(0.01)	0.21	(0.01)	0.16	(0.01)	-1.31	(0.00)
	Leu213	-1.13	(0.01)	-0.11	(0.01)	0.15	(0.01)	-1.24	(0.01)
	Phe249	-1.92	(0.01)	-0.09	(0.01)	0.63	(0.01)	-1.59	(0.01)
	Trp254	-3.60	(0.01)	-0.18	(0.01)	1.08	(0.01)	-3.08	(0.01)
	Trp260	-1.02	(0.01)	-1.11	(0.01)	1.33	(0.01)	-0.94	(0.01)
	Ile261	-0.58	(0.01)	-0.48	(0.00)	0.51	(0.00)	-0.67	(0.01)
	Phe274	-3.79	(0.01)	-3.43	(0.03)	3.35	(0.02)	-4.53	(0.02)
	Tyr306	-1.92	(0.01)	0.85	(0.01)	-0.52	(0.01)	-1.82	(0.01)
ATX-TGZ_1	Lys208	-1.18	(0.01)	-31.52	(0.12)	29.56	(0.11)	-3.24	(0.02)
	Thr209	-1.64	(0.01)	-7.50	(0.04)	5.59	(0.03)	-3.74	(0.02)
	Phe210	-2.48	(0.01)	-4.77	(0.03)	3.39	(0.01)	-4.14	(0.01)
	Leu213	-2.77	(0.01)	-3.35	(0.01)	2.70	(0.01)	-3.75	(0.01)
	Leu243	-0.92	(0.01)	-0.06	(0.01)	0.16	(0.01)	-1.00	(0.01)
	Phe273	-1.50	(0.01)	0.10	(0.01)	0.44	(0.00)	-1.12	(0.01)
	Phe274	-2.59	(0.01)	-0.78	(0.00)	1.16	(0.00)	-2.46	(0.01)
	Tyr306	-1.91	(0.01)	-0.45	(0.00)	0.99	(0.00)	-1.62	(0.01)

**ATX-TGZ_2**	Leu78	-1.07	(0.00)	-0.60	(0.00)	0.67	(0.00)	-1.18	(0.00)
	Phe210	-2.18	(0.01)	-0.45	(0.00)	0.78	(0.00)	-2.12	(0.01)
	Tyr214	-1.59	(0.01)	0.21	(0.00)	0.05	(0.00)	-1.45	(0.01)
	Lys248	-1.61	(0.01)	-16.12	(0.02)	16.30	(0.02)	-1.60	(0.01)
	Phe249	-2.33	(0.01)	1.22	(0.01)	-0.55	(0.01)	-1.94	(0.01)
	His251	-0.49	(0.01)	-4.95	(0.04)	4.47	(0.03)	-1.08	(0.01)
	Trp254	-2.27	(0.01)	-0.58	(0.01)	1.33	(0.01)	-1.81	(0.01)
	Pro258	-1.08	(0.00)	-0.67	(0.00)	0.59	(0.00)	-1.26	(0.01)
	Trp260	-2.30	(0.01)	-0.08	(0.01)	0.59	(0.01)	-2.10	(0.01)
	Phe274	-3.23	(0.01)	0.21	(0.01)	0.51	(0.00)	-3.00	(0.01)
**ATX-TGZ_3**	Leu78	-1.04	(0.01)	-0.01	(0.00)	0.11	(0.00)	-1.09	(0.01)
	Phe210	-1.69	(0.01)	-0.05	(0.00)	0.34	(0.00)	-1.69	(0.01)
	Leu243	-2.29	(0.01)	1.42	(0.01)	-0.97	(0.01)	-2.08	(0.01)
	Arg244	-1.68	(0.01)	-10.80	(0.02)	11.25	(0.03)	-1.52	(0.01)
	Lys248	-2.54	(0.01)	-23.77	(0.05)	23.95	(0.04)	-2.66	(0.01)
	Phe249	-1.86	(0.01)	0.85	(0.02)	-0.51	(0.01)	-1.73	(0.01)
	Trp254	-1.90	(0.01)	0.16	(0.01)	0.40	(0.01)	-1.54	(0.01)
	Phe274	-1.84	(0.01)	-0.53	(0.01)	0.88	(0.01)	-1.71	(0.01)
**ATX-TGZ_4**	Phe210	-1.90	(0.01)	-0.50	(0.01)	0.85	(0.01)	-1.86	(0.01)
	Leu213	-2.40	(0.01)	-2.55	(0.01)	1.81	(0.01)	-3.44	(0.01)
	Tyr214	-1.10	(0.00)	-0.38	(0.00)	0.48	(0.00)	-1.06	(0.00)
	Leu243	-1.19	(0.01)	1.53	(0.02)	-1.29	(0.02)	-1.10	(0.01)
	Arg244	-0.99	(0.01)	-29.39	(0.15)	27.64	(0.12)	-2.92	(0.03)
	Lys248	-0.41	(0.01)	-32.00	(0.15)	30.49	(0.13)	-2.05	(0.03)
	Phe273	-1.41	(0.01)	0.01	(0.00)	0.42	(0.00)	-1.12	(0.01)
	Phe274	-3.24	(0.01)	-1.92	(0.01)	1.90	(0.00)	-3.58	(0.01)
	Tyr306	-2.04	(0.01)	0.12	(0.00)	0.24	(0.00)	-1.91	(0.01)

Table S3. Structures and total binding energies (ΔG_{bind}) with ATX and PPAR$_\gamma$ complexes of tested compounds, as calculated with the MM–GBSA method. Related to Figures 1, 3, 4 and 5

Standard error of the mean is shown in parentheses. The suggested (by MD simulations) type of inhibition of the compounds against ATX is also included.

Compound	Label	Structure	ΔG_{bind} (kcal mol^{-1}) / ATX	ΔG_{bind} (kcal mol–1) / PPARγ	ATX inhibitor type
**Troglitazone (TGZ)**	TGZ_1	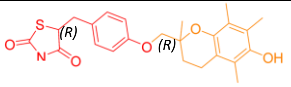	-54.21 (0.52)		Type II
	TGZ_2	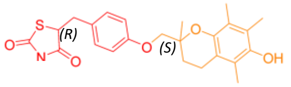	-40.51 (0.29)		
	TGZ_3	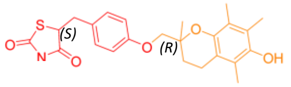	-35.61 (0.41)	-58.01 (0.21)	
	TGZ_4	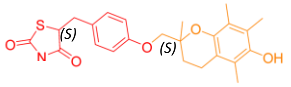	-53.59 (0.43)		Type II
**5**	5_1	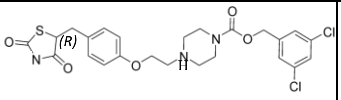	-40.84 (0.23)	-63.40 (0.17)	Type III
	5_2	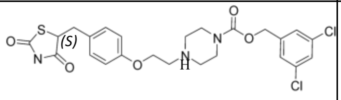	-60.86 (0.22)	-60.17 (0.20)	Type IV
**EL244**	EL244_1	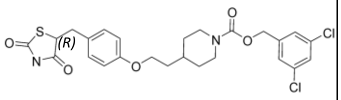	-76.62 (0.23)	-66.23 (0.32)	Type I
	EL244_2	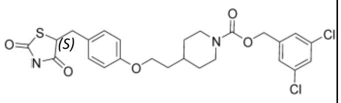	-46.01 (0.26)	-71.01 (0.16)	Type IV
**Rosiglitazone (RGZ)**	RGZ_1	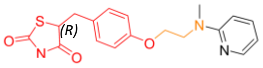			
	RGZ_2	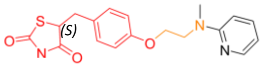			

				-47.23 (0.16)	
**Pioglitazone (PGZ)**	PGZ_1	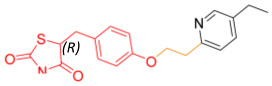			
	PGZ_2	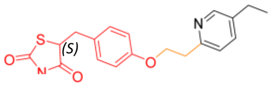		-49.76 (0.18)	

Table S4. Critical HDX-MS experimental information. Related to Figure 5

** Average deuterium recovery calculated using RRPYIL, DRVYIHPF and RPKPQQFFGLM-NH2 as model peptides.*

**Data Set**	**ATX**	**ATX + EL244**
HDX reaction details	10mM PBS, 150mM NaCl, pD=7.4 at 25°C	10mM PBS, 150mM NaCl, pD=7.4 at 25°C
HDX time course (s)	30, 300, 3000	30, 300, 3000
HDX control samples	none	none
Deuterium recovery (mean)	70%*	
# of Peptides	310	310
Sequence coverage	84.54%	84.54%
Average peptide length / Redundancy	12.48/5.62	12.48/5.62
Replicates (biological or technical)	4 (Technical)	4 (Technical)
Repeatability	0.0482 (average SD)	0.0629 (average SD)
Significant differences in HDX (delta HDX > X D)	0.26Da	0.26Da

Table S5. Calculated occurrence of water bridges observed in the ATX complexes examined. Related to Figures 1, 5 and S1

Only compounds where water bridges occur higher than 25% of the simulation time are shown.

Compound	ATX residues involved	Occurrence (%)
EL244_1	Asp311	46
EL244_2	Trp275	53
TGZ_1	–	–
TGZ_2	Glu67	60
	Trp260	59
	Arg74	27
TGZ_3	Thr272	37
TGZ_4	–	–

Table S6. Per-residue MM–GBSA energy decomposition in the PPAR$_\gamma$ complexes examined. Related to Figure S6

Only selected residues are shown. Uncertainties denote standard error of the mean (units in kcal mol^{-1}) and are included in parentheses.

Label	Residue	van der Waals		Electrostatic		Polar Solvation		Total energy	
PPARγ-5_1	Cys285	-1.61	(0.01)	-0.18	(0.01)	0.57	(0.01)	-1.38	(0.01)
	Gln286	-2.02	(0.01)	-7.91	(0.02)	7.71	(0.02)	-2.46	(0.01)
	Arg288	-1.28	(0.00)	2.10	(0.01)	-2.10	(0.01)	-1.49	(0.00)
	Ser289	-2.07	(0.01)	-1.04	(0.01)	1.52	(0.01)	-1.78	(0.01)
	His323	-2.28	(0.01)	-7.97	(0.02)	7.96	(0.01)	-2.56	(0.01)
	Ile326	-2.87	(0.01)	-0.04	(0.01)	0.02	(0.01)	-3.19	(0.01)
	Tyr327	-1.75	(0.01)	-1.21	(0.01)	1.22	(0.01)	-1.89	(0.01)
	Leu330	-1.42	(0.00)	0.36	(0.00)	-0.37	(0.00)	-1.58	(0.00)
	Phe363	-1.76	(0.01)	-2.15	(0.02)	2.26	(0.02)	-1.79	(0.01)
	Met364	-1.51	(0.01)	-0.39	(0.01)	0.39	(0.01)	-1.64	(0.01)
	Leu465	-1.47	(0.00)	-3.73	(0.01)	2.85	(0.00)	-2.42	(0.01)
	His466	-1.08	(0.01)	-0.52	(0.01)	-0.43	(0.01)	-2.11	(0.01)
	Leu469	-1.38	(0.00)	0.11	(0.01)	0.35	(0.00)	-1.00	(0.01)
	Gln470	-1.22	(0.00)	-1.84	(0.01)	1.73	(0.01)	-1.37	(0.00)
PPARγ-5_2	Cys285	-3.12	(0.01)	-2.66	(0.01)	3.02	(0.01)	-3.21	(0.01)
	Gln286	-1.42	(0.00)	-1.46	(0.03)	1.35	(0.02)	-1.58	(0.01)
	Arg288	-1.96	(0.01)	10.74	(0.02)	-10.19	(0.02)	-1.69	(0.01)
	Ser289	-0.46	(0.01)	-7.34	(0.02)	6.49	(0.01)	-1.37	(0.01)
	His323	-0.61	(0.01)	-6.91	(0.01)	4.94	(0.01)	-2.63	(0.01)
	Ile326	-1.52	(0.00)	-1.79	(0.00)	1.81	(0.00)	-1.74	(0.01)
	Leu330	-1.83	(0.01)	-0.59	(0.00)	0.64	(0.00)	-2.08	(0.01)
	Val339	-1.27	(0.01)	0.75	(0.00)	-0.67	(0.00)	-1.30	(0.01)
	Ile341	-1.71	(0.00)	1.02	(0.01)	-0.51	(0.00)	-1.42	(0.00)
	Phe363	-1.11	(0.01)	-0.36	(0.00)	0.52	(0.00)	-1.03	(0.00)
	Met364	-2.32	(0.01)	-0.78	(0.00)	1.10	(0.00)	-2.20	(0.01)
	Lys367	-0.96	(0.00)	-5.72	(0.01)	5.70	(0.01)	-1.03	(0.00)
	His449	-0.66	(0.01)	-6.18	(0.02)	4.24	(0.01)	-2.65	(0.01)
	Tyr473	-0.21	(0.01)	-6.35	(0.01)	5.66	(0.01)	-0.95	(0.01)
PPARγ-EL244_1	Arg280	-1.09	(0.00)	-10.7	(0.01)	10.38	(0.01)	-1.49	(0.00)
	Ile281	-2.83	(0.01)	0.38	(0.00)	-0.06	(0.00)	-2.75	(0.01)
	Phe282	-2.62	(0.01)	0.61	(0.01)	-0.25	(0.00)	-2.38	(0.01)
	Cys285	-2.74	(0.01)	-0.65	(0.01)	1.35	(0.01)	-2.53	(0.01)
	Gln286	-1.17	(0.01)	-5.91	(0.02)	4.40	(0.01)	-2.78	(0.01)
	Ile341	-2.21	(0.01)	-0.72	(0.00)	0.77	(0.00)	-2.45	(0.01)
	Met348	-1.37	(0.01)	-0.12	(0.00)	0.22	(0.00)	-1.38	(0.01)
	Phe363	-1.31	(0.01)	0.27	(0.01)	-0.12	(0.01)	-1.26	(0.01)
	Met364	-1.29	(0.00)	-0.08	(0.00)	0.14	(0.00)	-1.36	(0.00)
	Lys367	-1.06	(0.01)	-32.5	(0.11)	30.05	(0.09)	-3.68	(0.03)
	His449	-0.44	(0.01)	-5.66	(0.04)	3.92	(0.03)	-2.26	(0.02)
	Tyr473	-0.05	(0.01)	-5.46	(0.04)	5.01	(0.03)	-0.55	(0.01)
	Phe264	-2.67	(0.00)	-0.60	(0.00)	0.81	(0.00)	-2.70	(0.00)
	Arg280	-1.42	(0.00)	-10.6	(0.01)	10.79	(0.01)	-1.40	(0.00)
	Ile281	-2.38	(0.01)	0.40	(0.00)	-0.25	(0.00)	-2.39	(0.01)
	Gly284	-1.41	(0.01)	-0.39	(0.00)	0.60	(0.00)	-1.42	(0.01)
	Cys285	-3.30	(0.01)	-2.33	(0.01)	2.16	(0.01)	-3.77	(0.01)
	Gln286	-1.63	(0.00)	-2.13	(0.03)	1.90	(0.02)	-1.91	(0.01)

**PPARγ-EL244_2**	Arg288	-1.12	(0.01)	-12.8	(0.01)	12.91	(0.01)	-1.29	(0.01)
	Ser289	-0.30	(0.01)	-7.63	(0.02)	6.85	(0.01)	-1.15	(0.01)
	His323	-0.39	(0.01)	-7.37	(0.02)	5.05	(0.01)	-2.74	(0.01)
	Leu330	-1.15	(0.00)	-0.73	(0.00)	0.77	(0.00)	-1.29	(0.00)
	Ile341	-1.91	(0.01)	-0.11	(0.00)	0.28	(0.00)	-2.01	(0.01)
	Met348	-1.10	(0.00)	-0.66	(0.01)	0.55	(0.00)	-1.28	(0.00)
	Met364	-1.16	(0.00)	0.10	(0.01)	0.07	(0.01)	-1.11	(0.00)
	His449	-0.92	(0.01)	-7.10	(0.01)	5.02	(0.01)	-3.06	(0.01)
	Tyr473	-0.11	(0.01)	-7.28	(0.01)	6.29	(0.01)	-1.14	(0.01)
**PPARγ-PGZ_2**	Cys285	-3.49	(0.01)	-1.70	(0.01)	1.93	(0.01)	-3.59	(0.01)
	Gln286	-1.76	(0.01)	-5.55	(0.03)	4.12	(0.02)	-3.24	(0.01)
	Arg288	-1.34	(0.00)	-15.2	(0.01)	15.42	(0.01)	-1.43	(0.00)
	Ser289	-0.46	(0.01)	-7.84	(0.02)	7.05	(0.01)	-1.32	(0.01)
	His323	-0.37	(0.01)	-7.77	(0.02)	5.26	(0.01)	-2.92	(0.01)
	Leu330	-1.30	(0.00)	-0.73	(0.00)	0.76	(0.00)	-1.46	(0.00)
	Ile341	-1.82	(0.01)	-0.98	(0.00)	0.94	(0.00)	-2.11	(0.01)
	His449	-0.62	(0.01)	-6.38	(0.01)	4.72	(0.01)	-2.31	(0.00)
	Tyr473	-0.12	(0.01)	-7.09	(0.01)	6.22	(0.01)	-1.03	(0.01)
**PPARγ-RGZ_2**	Gly284	-0.86	(0.00)	-0.15	(0.01)	-0.03	(0.00)	-1.16	(0.00)
	Cys285	-3.56	(0.01)	-2.09	(0.01)	2.18	(0.01)	-3.79	(0.01)
	Gln286	-1.59	(0.00)	-2.83	(0.04)	2.30	(0.02)	-2.16	(0.02)
	Arg288	-1.33	(0.00)	-15.2	(0.01)	15.25	(0.01)	-1.54	(0.01)
	Ser289	-0.32	(0.01)	-7.71	(0.01)	6.91	(0.01)	-1.18	(0.01)
	His323	-0.48	(0.01)	-7.18	(0.01)	5.06	(0.01)	-2.62	(0.01)
	Ile326	-1.09	(0.00)	-0.82	(0.00)	0.85	(0.00)	-1.19	(0.00)
	Ile341	-1.61	(0.01)	-0.85	(0.00)	0.82	(0.00)	-1.86	(0.01)
	His449	-0.60	(0.01)	-7.16	(0.01)	4.94	(0.01)	-2.86	(0.01)
	Tyr473	-0.05	(0.01)	-7.32	(0.01)	6.41	(0.01)	-1.01	(0.01)
**PPARγ-TGZ_3**	Gly284	-1.10	(0.01)	-0.16	(0.01)	0.21	(0.01)	-1.20	(0.01)
	Cys285	-3.54	(0.01)	-1.89	(0.01)	1.89	(0.01)	-3.86	(0.01)
	Gln286	-1.62	(0.01)	-3.38	(0.04)	2.76	(0.02)	-2.28	(0.02)
	Arg288	-2.03	(0.01)	-13.8	(0.01)	14.17	(0.01)	-2.08	(0.01)
	Ser289	-0.38	(0.01)	-7.48	(0.01)	6.81	(0.01)	-1.12	(0.01)
	His323	-0.47	(0.01)	-7.10	(0.01)	4.97	(0.01)	-2.62	(0.01)
	Ile326	-1.07	(0.00)	-0.81	(0.00)	0.85	(0.00)	-1.16	(0.00)
	Leu330	-1.17	(0.00)	-0.68	(0.00)	0.72	(0.00)	-1.31	(0.00)
	Ile341	-2.61	(0.01)	-0.89	(0.00)	0.98	(0.00)	-2.77	(0.01)
	His449	-0.61	(0.01)	-7.03	(0.01)	4.87	(0.01)	-2.81	(0.01)
	Tyr473	-0.10	(0.01)	-7.40	(0.01)	6.38	(0.01)	-1.16	(0.01)
